# Systematic STR analysis of old post-vasectomy seminal fluid stains to examine evidence stored for 16 years

**DOI:** 10.1038/s41598-021-87937-x

**Published:** 2021-04-26

**Authors:** Julianna Kesselring Romero, Eloisa Auler Bittencourt, José Arnaldo Soares-Vieira, Ana Claudia Pacheco, Alexandre Learth Soares, Edna Sadayo Miazato Iwamura

**Affiliations:** 1 Laboratório de Patologia Molecular, Departamento de Patologia - Escola Paulista de Medicina/Universidade Federal de São Paulo (EPM/UNIFESP), Rua Botucatu 740, Edifício Lemos Torres. Vila Clementino, São Paulo, SP CEP 04023-62 Brazil; 2 Academia de Polícia de São Paulo (ACADEPOL), São Paulo, Brazil; 3 Departamento de Medicina Legal, Ética Médica, Medicina Social e do Trabalho- Faculdade de Medicina da Universidade São Paulo (USP), São Paulo, Brazil; 4 Instituto de Criminalística-Superintendência da Polícia Técnico-Científica do Estado de São Paulo (SPTC SP), São Paulo, Brazil

**Keywords:** Genetic techniques, Genotyping and haplotyping, PCR-based techniques, Biological techniques, Biotechnology

## Abstract

To understand stored evidence and the insertion in genetic databases is important in forensic investigations. Blood, pre- and post-vasectomy semen from 90 fertile male individuals, aged 24 to 45, were donated for research after informed consent. The semen samples were stored in the form of 30 µL stains on cotton fabric, for 16 years at room temperature in the laboratory. As well as the seminal fluid post vasectomy stains, which were performed after microscopy analyzes and certainty of the absence of spermatozoon. The pre vasectomy stains contained mainly haploid spermatozoon and the post vasectomy stains diploid epithelial cells and leukocytes. DNA extraction was performed with magnetic resin, followed by quantification and analysis of degradation of DNA. In this study we analyze these genetic profiles of DNA from stains on cotton fabric, using two Short Tandem Repeat multiplex systems, the PowerPlex Fusion 6C and Y23. Electrophoresis was performed on a 3500xL and analyzed using the Gene Mapper ID-X software. The genetic profiles of the 90 individuals were fully amplified in pre-vasectomy and partially in post-vasectomy stain samples, using the both multiplex systems. The results provide information about 0.25 cm^2^ semen stains on cotton fabric from 90 individuals, correlating concentration, degradation, and allele analysis. It also provides an understanding of the cells present in semen stains and the implications of individual factors. In the stains of post-vasectomy samples the small quantity of DNA was one of the limiting factors, in addition to degradation. Considering that all evaluations were carried out in a laboratory that has a quality control certificate and audited for being part of the national genetic profile database, the results were very consistent. Many aspects of the semen samples stored in the form of stains on cotton fabric have been clarified. The performance and sensitivity of the amplification systems used in the genotyping of azoospermic individuals were assessed. Conclusions: Genetic profiles were satisfactorily amplified in pre-vasectomy stain samples, and partially amplified in post-vasectomy stain samples, stored for almost two decades at room temperature in a tropical country. The small amount of DNA was one of the limitations in post-vasectomy stain samples, in addition to degradation and fragmentation. There are no publications in the literature on PowerPlex Fusion 6C and Y23 analyses using blood, sperm, and seminal fluids of the same individual, much less in the form of stains. This study can serve as a benchmark for the tracking analyses of stored samples. In addition, it anticipates a few social issues related to the analysis of post-vasectomy samples in forensic cases, most notably sex crimes.

## Introduction

In cases of sex crimes without a named suspect, it is possible to determine a genetic profile, using biological material (blood, semen, sweat, saliva) found on the victim, or in the form of stains, for later comparison with the profiles of any person suspected of other crimes. In this context, it is possible to locate the individual in a Database of Genetic Profiles. Many countries use autosomal Short Tandem Repeats (STR) profiles in DNA databases with different realities^1–3^. Y-STRs are valuable in the investigation of sexual assault cases in which autosomal STR genotype interpretation is challenging^4–7^. For the other hand, commercial kits with high resolution and high sensitivity are currently available for the widespread application of Y-STR analyses^8–13^.

In forensic cases, there is a need to work with biological samples containing increasingly scarce amounts of DNA^14–17^. In addition, there are cases of inconclusive or unsolved traces that are reopened for analysis. Notably, semen samples in the form of stains are frequent in sex crimes^18,19^. Technologies evolve and social behaviors change. Vasectomy, which is a simple procedure, has been offered free of charge by the Unified Health System (SUS) since 2008 in Brazil. In recent years, there has been an increase in vasectomies, due to either personal, economic or social reasons, especially in the State of São Paulo^20^.

Systematic studies on semen stains with known individuals are lacking, most of which are publications of unknown forensic samples or case reports^21–23^. To analyse the value of current kits such as PowerPlex Y23 and PowerPlex Fusion 6C systems, is also necessary.

The aim of this study was to evaluate the DNA extracted from stains, stored for 16 years in cotton fabric, containing semen and genitourinary cells present in the seminal fluid of vasectomized individuals. Three biological sources (blood and pre- and post-vasectomy semen), from fertile males aged between 24 and 45, were donated for research after informed consent.

## Methods

### Ethical approval

This study was approved by the Ethics Committee of the EPM/UNIFESP and Plataforma Brasil, nº CAEE 54983614.4.0000.5505. Informed consent was obtained from all individual participants and data were anonymized for this study. All methods were performed in accordance with the relevant guidelines and regulations.

### Biological samples (blood, pre- and post-vasectomy semen stains), procedures and characterization of DNA samples

#### Sample attributes

In 2004, a group of male individuals volunteered for vasectomy surgery at the Urology Department of the Faculty of Medicine of the University of São Paulo, having donated biological samples before and after the procedure (semen and blood). Samples were donated pre-vasectomy and after 20 ejaculations or at 3 months following vasectomy. To confirm the success of vasectomy the microscopic evaluation was performed. The vasectomy control procedure was performed according to World Health Organization’s (WHO) protocols^24^. A drop of pre-vasectomy semen or post-vasectomy ejaculate was scanned under a microscope, using a slide and a coverslip. In the absence of spermatozoa, the post-vasectomy ejaculate was centrifuged and a drop of sediment was scanned under the microscope for the presence of spermatozoa. A large number of epithelial cells and few leukocytes were observed in the latter, overall. Informed consents have been signed by all donors of the samples. The Ethics Committee for Research Project Analysis approved the study^25^.

#### Present study

Semen stains (pre- and post-vasectomy) of 90 individuals were produced on cotton fabric and stored for 16 years (from 2004 to 2019), inside cardboard boxes at room temperature (16-22ºC). We used three biological samples of the same individual: blood, *in natura*, as control, and 30 µL pre- and post-vasectomy semen stains (Supplementary Fig. S1). The 30 µL of ejaculate produced stains of 2 cm in diameter. The stains were delimited by a circle drawn with a ballpoint pen, for better visualization. In all samples, yellowish stains were observed on the cotton fabric.

#### DNA extraction

The extraction of DNA from blood, *in natura,* was performed using the salting-out method and served as control in all analyses^26^. The stains on the cotton fabric were delimited by a circle drawn with a ballpoint pen at the time they were made, for better visualization. Considering that in forensic analysis, stains are collected in a variety of sizes and conditions, we used the smallest possible quantities to simulate real forensic cases. The central area was cut out from each delimited circle, using a scalpel on the cotton fabric, resulting in 0.25 cm^2^ cuttings and the material was placed in a 1.5 mL tube. The DNA of the semen stains on cotton fabric, pre- and post-vasectomy, was obtained using the DNA IQ System (Promega, Madison, USA), as recommended^27^.

#### DNA quantification

The quantity of DNA extracted from both samples (pre- and post-vasectomy stains) and blood was determined by real-time PCR using the Quantifiler Trio DNA Quantification Kit (Applied Biosystems, USA) according to the manufacturer's instructions^28^. The reactions were run on an ABI PRISM 7500 Real-Time PCR system (Applied Biosystems, USA). The data were analyzed using the HID Real-Time PCR Analysis Software v 1.2 (Applied Biosystems, USA).

#### Analysis of the degradation of the samples

In addition to DNA concentration, the Quantifiler^Ⓡ^ Human DNA Quantification Kit (Applied Biosystems, CA, USA) demonstrates sample degradation levels through the large DNA/small DNA ratio. Values less than 1.5 indicate that the sample is not degraded, while values greater than 1.5 indicate that the material is degraded^28^.

#### PCR amplification

At least 270 samples (from blood and pre- and post-vasectomy semen stains) were quantified (Table S1). Volumes were adjusted according to the defined protocol. Amplification was performed using the PowerPlex Y23 System^29^, which amplifies 22 Y chromosome short tandem repeat *loci* and Amelogenin, as well as the PowerPlex Fusion 6C System^30^, which amplifies all *loci* on the expanded CODIS core *loci*, including 23 autosomal STR, 3 Y-STR and Amelogenin (both systems, Promega, Madison, USA). Reaction set up and thermal cycling were performed according to the User´s Manual^31^.

#### DNA Typing and evaluation of profiling data

PCR products of the PowerPlex Y23 and PowerPlex Fusion 6C Systems were separated and detected by capillary electrophoresis on a 3500 xL Genetic Analyzer (Applied Biosystems, USA). Data collection was conducted using the 3500 Series Data Collection Software 2 (Applied Biosystems, USA), and data analysis was performed using the GeneMapper ID-X Software v.1.4 (Applied Biosystems, CA, USA). The peak amplitude threshold was set at 50 RFU. Capillary electrophoresis was performed with run parameters as outlined in the PowerPlex Y23 and PowerPlex Fusion 6C Systems Manual^29,31^.

#### Statistics and analysis

The correlation between the 90 pairs of measurements collected pre- and post- vasectomy was calculated using the Spearman’s correlation coefficient ρ^32^, a nonparametric alternative, considered due to the distribution of data (not normally distributed). This measure is calculated based on the measurement stations. The calculations presented in this study were performed using the R 3.6.0 software^33^, graphs were drawn using the ggplot2^34^ and ggpubr ^35^ packages.

## Results

### DNA quantification and amplification

In summary, not all post- vasectomy samples contained sufficient quantities for the genetic profiles to be amplified. When values were insufficient for amplification, the process was repeated with increased volume. The volume of DNA extracted was adjusted when possible and diluted if necessary, as shown in Table S1. In the PowerPlex Fusion 6C System^30,31^ the sample volumes for DNA amplification obtained from pre-vasectomy stains were: 72 samples with dilution and application of 1–2 µl; the other samples without dilution with 0.5–2 µl application. For blood DNA that has been previously extracted and frozen, there were, except 3 samples, all 87 with dilution and application of 0.5–2 µl. For DNA extracted from stains containing post-vasectomy seminal fluid there were 55 samples with 10 and 15 µl application and 33 samples with 0.5–5 µl application. Similar results were obtained for the PowerPlex 23Y System.

### Analysis of DNA concentration and degradation index

The samples extracted from blood, pre-vasectomy semen and post-vasectomy seminal fluid stains were analysed using the Quantifiler Trio Kit. In addition to DNA concentration, the kit demonstrates degradation levels in samples through the large DNA/ small DNA ratio (degradation index). Degradation index (DI) values less than 1.5 indicate that the sample is not degraded, while DI values greater than 4 indicate that the material is degraded^36^. In the present study, for the DNA extracted from pre-vasectomy stains, there were 2 samples with values below 1.0; 88 samples with values between 1 and 5, and no samples with values greater than 5.0. For blood samples, there were 3 samples with values below 1.0; 84 samples with values between 1 and 5 and no samples with values greater than 5.0. For the DNA extracted from post-vasectomy stains, there were no samples with values below 1.0; 80 samples with values from 1 to 5; 6 samples with values from 5 to 10 and 4 samples with values above 10.

**Graph 1:**
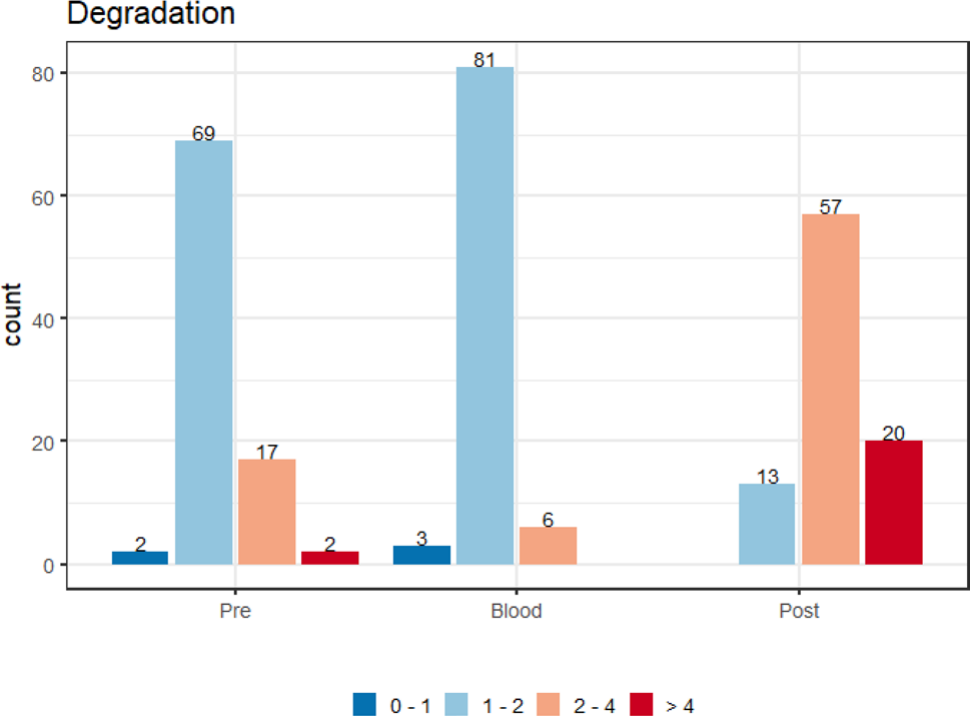
Absolute frequency per DNA degradation index ranges, from 0–1, 1–2, 2–4 and > 4, of blood, pré- and post-vasectomy seminal fluid stains samples (n = 90 individuals).

The graph showing the DI ranges for each sample was produced based on the degradation measurements of three sources: pre-vasectomy semen stains, blood, and post-vasectomy seminal fluid stains. Graph 1 shows DI ranges from 0 to 1, from 1 to 2, from 2 to 4 and greater than 4, which were used for better visualization. The absolute frequency per DI range after DNA extraction was analyzed according to the Quantifiler Trio kit protocol^28,36^.

### Correlation between DNA concentration in µg/µl and PowerPlex Y23 and PowerPlex Fusion 6C Systems profiles

The PowerPlex Y23 Systems (*loci* DYS576, DYS389I, DYS448, DYS389II, DYS1, DYS391, DYS481, DYS549, DYS533, DYS438, DYS437, DYS570, DYS635, DYS390, DYS439, DYS392, DYS643; DYS393, DYS458, DYS385a/b, DYS456, Y-GATA-H4) and the PowerPlex Fusion 6C Systems (*loci* D3S1358, D1S1656, D2S441, D10S1248, D13S317, Penta E, D16S539, D18S51, D2S1338, CSF1PO, Penta D, TH01, vWA, D21S11, D7S820, D5S818,TPOX, D8S1179, D12S391, D19S433, SE33, D22S1045, DYS391, FGA, DYS576 and DYS570) alleles typing are shown in Table S2.

The profiles on the electropherogram were obtained from Table S3. Of these: those with no results were labeled as 0 (zero). Cases where at least one of the alleles or doubtful profile were labeled as 0.5. Cases with the results of profile for heterozygotes or for homozygotes, were labeled as 1. Thus, for each individual, this count totaled 23 *loci* (PowerPlex Y23) and 27 *loci* (PowerPlex Fusion 6C) for each sample from the three sources.

The correlation between allele profile (Table S2) and the DNA concentration (μg/ml) was performed using data from the article by Soares-Vieira et al.^25^. The samples were identified with two different codes (codes 1 to 90 for the study by Soares Vieira et al., from where we obtained the DNA concentration of fresh samples in natura and 2364 to 2657 in the present study for the profiles of the PowerPlex Fusion 6C and PowerPlex Y23). Thus, among the 90 individuals, comparing the minimal haplotypes (7 Y-STR *loci*), we identified 78 individuals with the same genetic profile.

Table 1 shows Spearman's correlation between the number of markers with complete profile and the corresponding DNA concentration. We found a positive correlation for the 4 cases (although low). That is, in general, in higher concentrations there is a greater number of markers (*loci*) that are possible to obtain the genetic profiles. However, in pre-vasectomy stain samples and 27 *loci*, the correlation is not significant (p-value = 0.402).

**Table 1:** Spearman correlation (ρ) between measures of DNA concentration and count of markers of PowerPlex Y23 and PowerPlex Fusion 6C Systems from pre- and post-vasectomy stain samples (90 individuals).

Semen stain samples	Number of genetic markers PowerPlex Y23 and PowerPlex Fusion 6C Systems	ρ	p-valor
Pre vasectomy	23	0.23	0.047
	27	0.10	0.402
Post vasectomy	23	0.25	0.029
	27	0.29	0.009

### Correlation between degradation index and the count of genetic markers (*loci*) with the amplification of the 23 *loci* of PowerPlex Y23 and the 27 *loci* of PowerPlex Fusion 6C System

**Graph 2:**
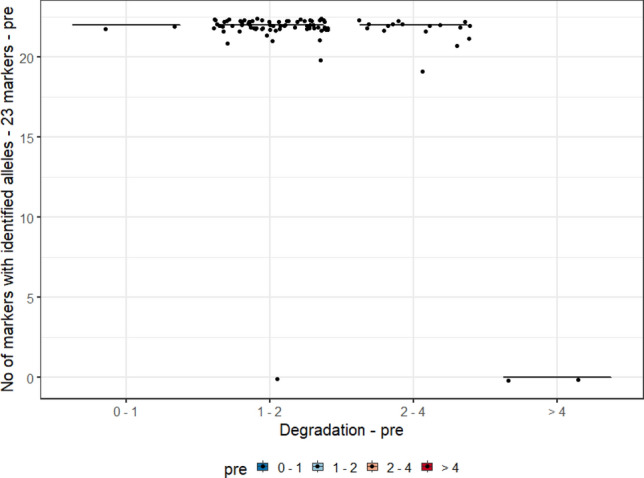
Boxplots of the degradation index range and genetic profile of PowerPlex Y23 System of the pre- vasectomy samples (n = 90 individuals).

**Graph 3:**
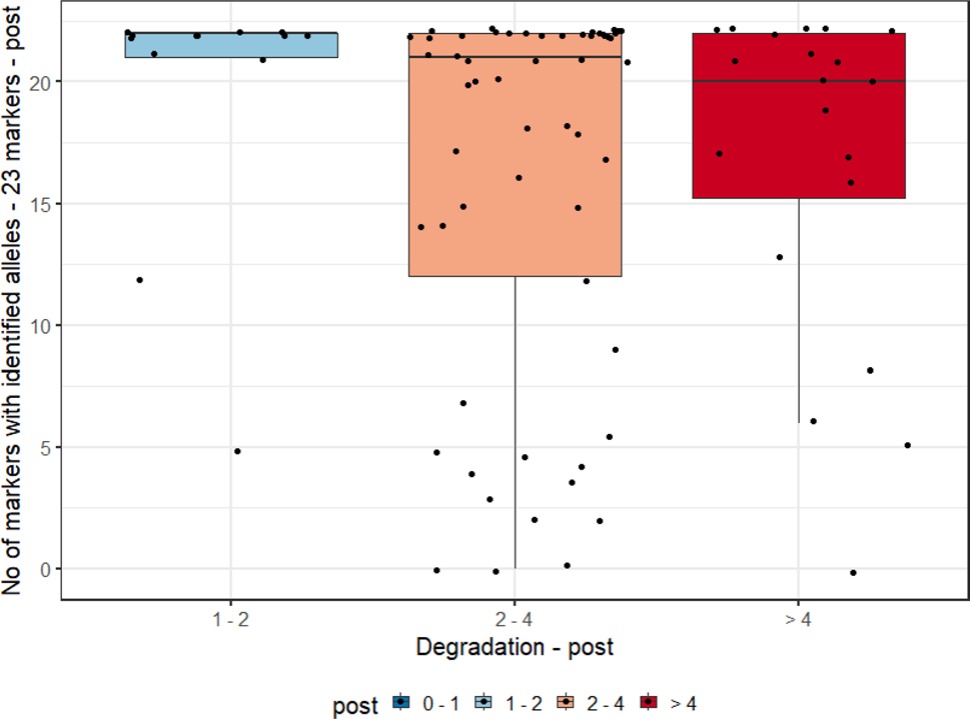
Boxplots of the degradation index range and genetic profile of the PowerPlex Y23 System of the post- vasectomy samples (n = 90 individuals).

**Graph 4:**
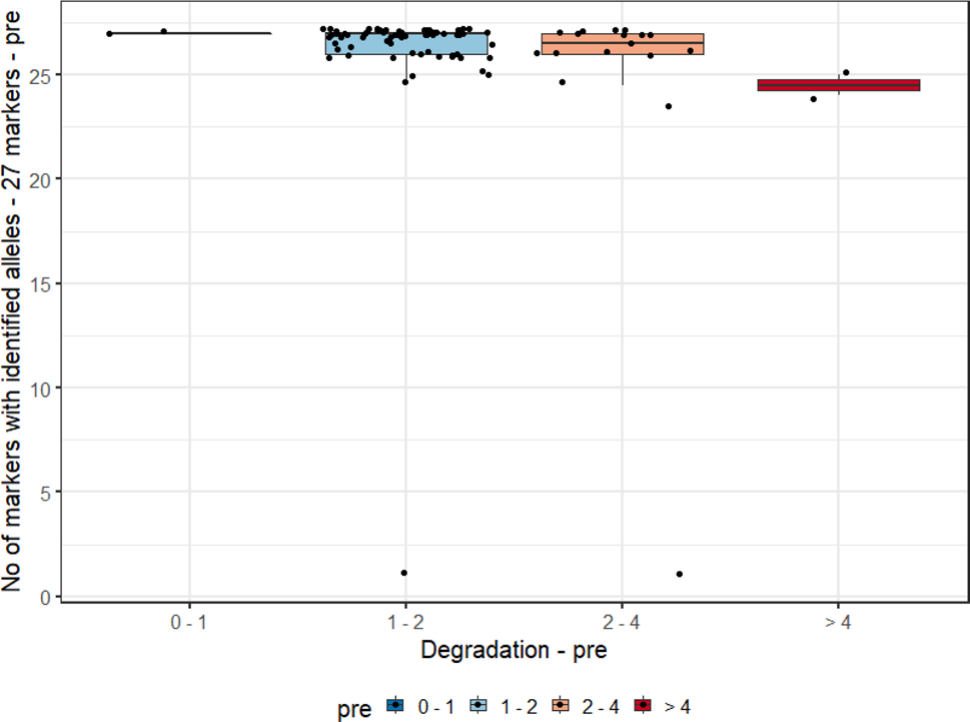
Boxplots of the degradation index range and genetic profile of PowerPlex Fusion 6C System of pre- vasectomy samples (n = 90 individuals).

**Graph 5:**
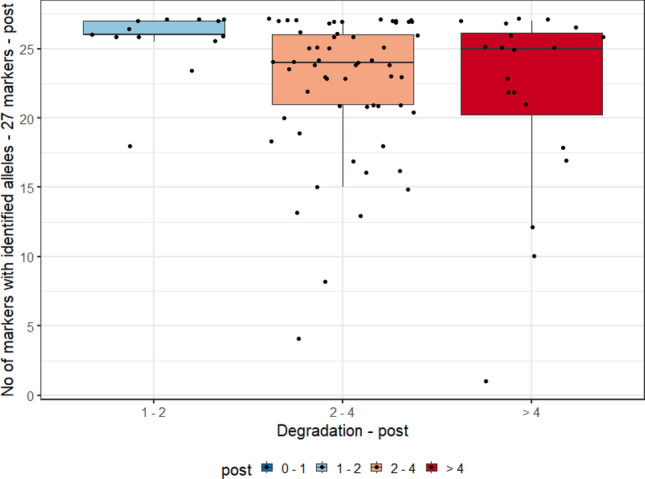
Boxplots of the degradation index range and genetic profile of PowerPlex Fusion 6C System of post- vasectomy samples (n = 90 individuals).

For this analysis, we evaluated the degradation index (DI) range measurements of the three biological samples (blood, and pre- and post-vasectomy semen stains) and the allele count, of 90 individuals typed by the PowerPlex Y23 and PowerPlex Fusion 6 C Systems. In Graphs 2, 3, 4 and 5, they were evaluated as DI ranges and not as the DI itself, as, in many cases, values are too close to each other but with a wide range of measurements (for post-vasectomy measurements, we have a DI of 100), which makes it difficult to view the data on the graph.

Correlations between the DI and the genetic profile were calculated with the PowerPlex Y23 and PowerPlex Fusion 6C systems, with typing 23 or 27 *loci* respectively (Table 2). It is important to note that all correlations are negative, which means that there is a tendency that the higher the degradation index, the lower the allele count. All correlations are relevant, but weak. The strongest correlation (ρ = − 0.33) observed was between the measurement of pre-vasectomy semen stains and the count of the 27 markers of PowerPlex Fusion 6C.

**Table 2:** Spearman correlation (ρ) between measures of degradation index and count of markers of the PowerPlex Y23 and PowerPlex Fusion 6C Systems, from pre and post vasectomy samples (90 individuals).

Semen samples	Number of genetic markers PowerPlex Y23 and PowerPlex Fusion 6C Systems	ρ	p-valor
Pre vasectomy	23	− 0.22	0.038
	27	− 0.33	0.002
Post vasectomy	23	− 0.30	0.004
	27	− 0.27	0.009

Graphs 2 and 4 show, respectively, the boxplots of the degradation index range and genetic profile of PowerPlex Y23 and Fusion 6 C Systems of the pre- vasectomy samples (n = 90 individuals). Among post-vasectomy seminal fluid measurements, there was a greater number of cases with incomplete profiles, with greater variation (Graphs 3 and 5).

### Comparison between the genetic profiles of pre- and post-vasectomy and blood samples with amplification using PowerPlex Y23 and PowerPlex Fusion 6C Systems

The analyses were performed by two experienced independent investigators and repeated in the pre-vasectomy stains samples, if the genetic profiles were not consistent. Table S4 presents data from DNA of blood and stains of pre vasectomy semen. In general, deletions and null alleles seem not to occur between the genetic profiles obtained from DNA extracted from blood and semen pre-vasectomy stains, of the same 90 individuals, using the PowerPlex Y23 and PowerPlex Fusion 6C Systems. However, more studies are necessary to answer if the post vasectomy samples presented the same profile, as it was only possible to analyze 43/90 samples, as repetition was not possible.

As for post-vasectomy seminal fluid stains, we obtained partial results, comparisons were made between the genetic profiles of PowerPlex Y23 (22 STR-Y) and PowerPlex Fusion 6C Systems, obtained from semen pre vasectomy stains and blood (Table S2).

The results of the genetic profiles of blood and pre-vasectomy semen stains of 90 individuals were obtained in their entirety using PowerPlex Y23. The Y-haplotypes are available in the R63 of the YHRD (YA004685) and Table S3, as well as the frequency in Table S4.

### DNA concentration pre- and post-vasectomy

**Graph 6:**
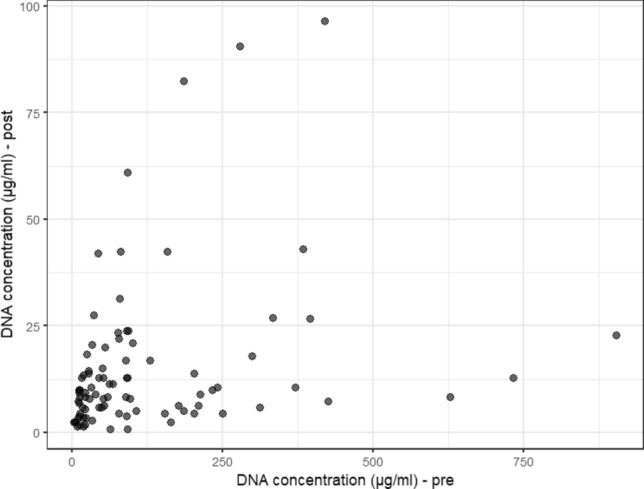
Relationship between semen DNA concentrations (μg/ml) pre-and post-vasectomy (n = 90 individuals).

**Graph 7:**
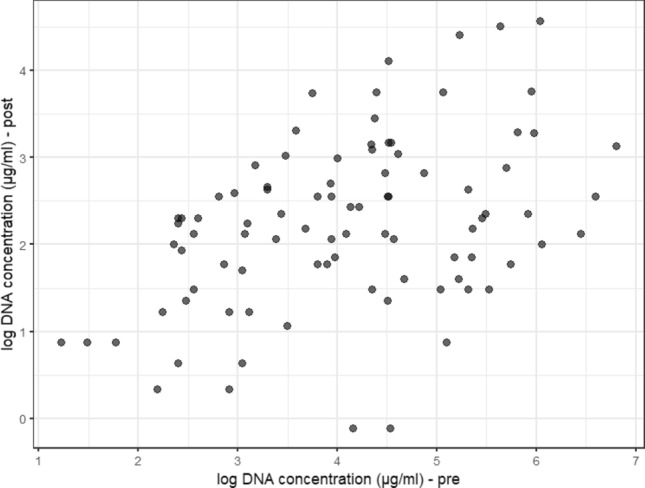
Relationship between the logarithms of DNA concentrations (μg/ml) pre- and post-vasectomy.

Scatterplots showing DNA concentration pre- and post-vasectomy were generated in two different ways: with the original concentration (μg/ml) (Graph 6) and the concentration in logarithmic scale—log (μg/ml) (Graph 7). Due to the asymmetric distribution, the calculation of the logarithm facilitates the visualization of data, which were originally very condensed in the lower values. It can be seen that for the same post-vasectomy, we have different pre-vasectomy concentration measurements. The correlation was calculated using Spearman's coefficient ρ^32^, a non-parametric alternative, and considered due to the distribution of data (not normally distributed). The Spearman’s correlation between the 90 pairs of measurements collected pre- and post- vasectomy was ρ = 0.38 (p-value < 0.001).

### Analysis of the profiles of the São Paulo City population sample (Brazilian samples) using the PowerPlex Y23 and PowerPlex Fusion 6C Systems

The Genetic profiles and frequency data using the PowerPlex Y23 and PowerPlex Fusion 6C Systems are shown in Tables S4 and S5.

## Discussion

In our study, it is known that there were no female cells, only male cells. Thus, DNA was extracted directly and a differential extraction procedure was not necessary.

In the study of Soares-Vieira et al. the patients returned for the control exam and certification of the success of the vasectomy surgery. Of a total of 90 patients, only 1 individual presented post-vasectomy recanalization and was excluded^25^. In a few cases it was possible to find sperm cells in the ejaculate, sometimes without motility, that is, dead. Thus, some patients had to return for confirmatory analysis. The ejaculate samples were transformed into stains after the certainty that there were no spermatozoon visualized under the microscope.

To discuss the factors that may lead to degradation, we explain that all samples were stored for 16 years, under the same conditions of humidity and temperature. As for the age of sample donors, most were between 30 and 40. According to reports, the sexual abstinence period varied between 12 h and 30 days, which are not very reliable data. If these individuals were undergoing infertility treatment, this information would surely be more reliable, but that was not the case.

We are familiar with the difficulties inherent in obtaining enough cellular material for analysis at crime scenes. The general perception is that the presence of a large number of spermatozoa in semen samples, even when samples are small, allows for a satisfactory analysis. However, this is not always true. In the study by Soares-Vieira et al., a great variation in the DNA concentration was observed in pre-vasectomy semen samples from fertile individuals. And this variability was widely discussed^25^. In the study by Mautoni et al., in some samples (25%), the concentration of DNA extracted from the post-vasectomy ejaculate was equal to or greater than the amount obtained from the pre-vasectomy punch^37^.

Dispersion graphs of DNA concentration measurements pre- and post-vasectomy were constructed in two ways: with the original measurements (μg/ml) (Graph 6) and with the logarithms of the measurements—log (μg/ml) (Graph 7). Due to the asymmetric distribution, the calculation of the logarithm facilitates the visualization of the data, which were originally well condensed in the lower values. It can be seen that for the same concentration post-, we have different pre-vasectomy concentrations. In Graphs 6 and 7, we present the analysis of DNA concentration in pre- and post-vasectomy semen samples, *in natura*, in other words in fresh samples. Graphs S1a and S1b show the analysis of DNA concentration in stains of pre- and post-vasectomy semen samples in ng/ml. These concentrations do not present a significant correlation, unlike the fresh, *in natura* analysis, where the samples are more representative (μg/ml).

The present study presents a great improvement compared to the previous study^37^, in many aspects: (a) we used 0.25 cm^2^ of cotton fabric for DNA extraction, based on samples and traces received by crime laboratories, which are often the smallest possible sample. In the previous study, three samples of 1.5 cm in diameter were used; (b) As for DNA extraction, the paramagnetic resin DNA IQ (Promega, USA) and Quantifiler Trio systems were used to quantify and assess degradation. In the previous study, the QIAamp DNA Mini Kit (Qiagen, Hilden Germany) was used for DNA extraction, followed by quantification using NanoDrop; (c) pre- and post-vasectomy stains from 90 individuals were analyzed using the PowerPlex Y23 and PowerPlex Fusion 6C Systems. The positive peak height threshold was set at > 50 relative fluorescent units (RFUs). In the previous study, the genetic profiles of 28 individuals were obtained using the PowerPlex ESI 17 Pro System (Promega, USA) with the threshold set at > 150 RFUs.

In pre-vasectomy stain samples, 62/90 of DNA extracted had to be diluted and 1 or 2 ul used for amplification. On the other hand, part of the post-vasectomy semen stain samples could not be analyzed, using 15 µL in 43/90 of samples and 2 to 10 µL in 33/90 samples of DNA extracted, likely due to the degradation and fragmentation of the nuclei of the cells present in the stains. This could also be observed in electropherogram peaks, with a gradual decrease in RFU intensity as the amplicon size increases. One of the reasons for the failure in reading and typing the profile after amplification with PowerPlex Y23 and PowerPlex Fusion 6C Systems is the condition of the shedding epithelial cells. These cells detach from the lining epithelium due to their terminal development cycle and, in these cases, the nuclei may be fragmented or even more compacted, compared to a cell with the capacity to synthetize. According to van Oorschot et al. review, understanding the variables impacting DNA transfer, persistence, prevalence and recovery has become increasingly relevant in investigations of criminal activities to provide opinion on how the DNA of a person of interest became present within the sample collected^38^.

The values for the amplification of DNA extracted from the stains were adjusted after quantification. Thus, the initial concentration of pre- and post-vasectomy semen *in natura* (Soares- Vieira et al.) was used in the correlation analyses^25^. In addition, the Y profiles from PowerPlex Y23 were paired with the Y profiles (minimal haplotypes) from the previous study, as they refer to the same individuals. However, in case of disagreement between 1 or 2 alleles, these were not considered. In the study by Soares-Vieira et al., the multiplex system was standardized in house and the analyses were performed without the aid of software tools. In this sense, there may have been estimated reads (genetic profiles) based on the size of the fragments. Therefore, 12 samples were not included in this present analysis.

The profiles on the electropherogram were obtained from Table S3. Of these: those marked as OL (off-ladder), *, written entirely in red or left blank (that is, inconsistent or with no results) were labeled as 0 (zero). Cases where one of the profiles is in red font and the other is in black font, those that had the exact same profile for at least one of the alleles or doubtful profile (examples: 31 | 37? 15? OL, 15 | 19 | 19 | 24? 20 | OL?) were labeled as 0.5. For example: sample 2476 (PowerPlex Fusion 6C, D21S11 Blood 28/29.1/32.2? pre-vasectomy semen stains 28/32.2 and post-vasectomy semen stains *). Another example: sample 2514 (PowerPlex Fusion 6C, D21S11 Blood 30.1/31/32.1/32.2, pre-vasectomy semen stains 31/32.2 and post-vasectomy semen stains 31/32.2). Cases with the profile in black font, for example 11 | 12 for heterozygotes and 11 for homozygotes, were labeled as 1. Thus, for each individual, this count totaled 23 *loci* (PowerPlex Y23) and 27 *loci* (PowerPlex Fusion 6C) for each sample from the three sources (Table S3). Tables 1 and 2 were respectively between measures of DNA concentration and count of markers of PowerPlex Y23 and PowerPlex Fusion 6C Systems, as well as degradation index and count of markers of Y23 and Fusion 6C Systems from pre- and post-vasectomy stain samples (90 individuals). The amplification reactions were repeated in DNA extracted from blood and semen stains pre-vasectomy. Dilutions and adjustments were made to the DNA volume. Thus, we were able to evaluate the profiles of both systems from these two cell types (diploid cells from blood and haploid cells from semen pre-vasectomy). To safely state whether inconsistencies such as mutations, deletions and null alleles among the Y STR genetic profiles of blood and semen pre-vasectomy, from the same individuals, still need further studies. In the stains of post- vasectomy samples, volumes of up to 15µL of extracted DNA were used in many cases. The small quantity of DNA was one of the limiting factors, in addition to degradation.

Exceptionally, we have samples without alleles for both systems for DNA of semen pre vasectomy stains: for example, sample 2553, *locus* D2251045 allele 16/17 in blood; sample 2554, *locus* DYS437 allele 15 (S3). For the other hand, sample 2548 presented for the three types of sample, blood, pre- and post- vasectomy stains at *locus* D125391 alleles 20/OL (out of ladder). Indicating a new allele, demonstrating its specificity in haploid and diploid cells (S2).

Many of the aspects addressed in this study would be applied to stored samples that await insertion in the National Database of Genetic Profiles. In addition to demonstrating the analysis of post-vasectomy seminal fluid in the form of stains, in acceptable conditions according to the quality assurance standards of the Integrated Network of Genetic Profile Database (Rede Integrada de Perfis Genéticos -RIBPG), we addressed the problem in the context of the situation in Brazil^3^. The Unified Health System (Sistema Único de Saude—SUS) performs a series of elective surgeries (non-emergency), free of charge, including vasectomy. The prerequisites for vasectomy are: men must be over 25 years of age and have at least 2 children. Vasectomies are much faster and simpler than tubal ligation (tying of the fallopian tubes, which requires hospitalization and offers more risks to women). There has been an increasing acceptance of and demand for vasectomy in Brazil, confirmed by the increase in the number of procedures reported by the Unified Health System^20^. Science seeks to anticipate potential problems. In this sense, this study is of unique importance.

There are no data indicating an increase in the number of vasectomized individuals involved in sexual violence cases. However, it is known that domestic violence and sexual violence cases often go underreported. In cases where information is available, it can be observed that 58.9% of femicides occur in a private residence and that, in 89.9% of cases, the perpetrator is a partner or ex-partner of the victim. Data from the 2020 Brazilian Public Security Yearbook show that, in Brazil, there is at least one rape every 8 min; 66,123 reports of rape in vulnerable populations were registered at police stations. However, these numbers account only for the visible side of sex crimes, which are those reported to the police. There is a massive underreporting of these cases, due to fear, feelings of guilt and shame on the part of the victim; fear of the aggressor and even discouragement by the authorities. Rape is the only crime in which the victim is the one to feel guilt and shame. According to current estimates, this number can be up to ten times greater, but there is not enough studies and research on the issue^39^.

This is a pioneering study on this type of analysis. When assessing forensic samples of semen stains and case reports^21–23^, it is often difficult to obtain information about individuals, and the samples often contain mixtures and inhibitors. The present study can be used as a reference for forensic analysis in cases of stored semen stains.

The present study clarifies, in a systematic way, the analysis of semen samples in the form of stains. The DNA profile obtained from blood and semen stains samples of the same individuals, using the PowerPlex Y23 and PowerPlex Fusion 6C systems, is highlighted. The results provide information about 0.25 cm^2^ semen stains on cotton fabric from 90 individuals, correlating concentration, degradation, and allele analysis. It also provides an understanding of the cells present in semen stains and the implications of individual factors, such as variability conditioned to several factors, time of sexual abstinence, habits of life, as well as vasectomy or oligospermia.

## Conclusions

This study analyzed the most recent multiplexes capable of analyzing DNA with more *loci* until now. We present the analysis of autosomal and Y-STR multiplex systems from blood diploid and semen haploid cells pre-vasectomy DNA, recovered from stored stains. The small amount and degradation of cells were limitations for the non-amplification in part of the post-vasectomy samples. That would be mainly due to the terminal cycle of the cells that cover the genitourinary tract, which are shed from the epithelium and eliminated. Considering that all evaluations were carried out in a laboratory that has a quality control certificate, in addition to being audited for being part of the Integrated Network of Genetic Profile Database (RIBPG), the DNA extracted from the 90 individuals exhibited a satisfactory profile in all pre-vasectomy semen stains stored for 16 years at room temperature in tropical climate. Finally, we evaluated the performance and sensitivity of the amplification systems used in the genotyping of azoospermic individuals, aiming at obtaining adequate profiles that can be inserted in the Genetic Profile Database. The present study can be used as a benchmark for forensic analysis in cases of stored trace samples. Also, it has anticipated some social issues concerning the analysis of samples from vasectomized individuals in forensic casework.

## Supplementary Information

Supplementary Information 1.

Supplementary Information 2.

Supplementary Information 3.

Supplementary Information 4.

Supplementary Information 5.

Supplementary Information 6.

Supplementary Information 7.
