# Supplementary material for: Systematic STR analysis of old post-vasectomy seminal fluid stains to examine evidence stored for 16 years
Source: Sci Rep. 2021 Apr 26;11:8918. doi: 10.1038/s41598-021-87937-x (PMC8076208; doi:10.1038/s41598-021-87937-x)

**Supplementary information**

**Systematic STR analysis of old post-vasectomy seminal fluid stains to examine evidence stored for 16 years**

Julianna Kesselring Romero^1^, Eloisa Auler Bittencourt^1,2^, José Arnaldo Soares-Vieira^3^, Ana Claudia Pacheco^4^, Alexandre Learth Soares^4^, Edna Sadayo Miazato Iwamura ^1^*

^1^ Laboratório de Patologia Molecular, Departamento de Patologia - Escola Paulista de Medicina /Universidade Federal de São Paulo (EPM/UNIFESP), SP, Brazil; ^2^ Academia de Polícia de São Paulo (ACADEPOL), SP, Brazil; ^3^ Departamento de Medicina Legal, Ética Médica, Medicina Social e do Trabalho- Faculdade de Medicina da Universidade São Paulo (USP), SP, Brazil; ^4^  Instituto de Criminalística-Superintendência da Polícia Técnico-Científica do Estado de São Paulo (SPTC SP), SP, Brazil

* Corresponding author : Edna Sadayo Miazato Iwamura, phone +55 11 5576 4848 ext 1386, e-mail: edna.iwamura@unifesp.com

Departamento de Patologia, Escola Paulista de Medicina/Universidade Federal de São Paulo (EPM/UNIFESP), Rua Botucatu 740, Edifício Lemos Torres. Vila Clementino- CEP 04023-62, São Paulo, SP- Brazil.

**Figure 1.** The cotton fabrics containing the ejaculate stains were stored for 16 years (from 2004 until 2019) at room temperature (16-22ºC), inside cardboard boxes. The 30µL of ejaculate produced 2 cm stains in diameter. The stains were delimited by a circle, made with a ballpoint pen, for better visualization. From each circle delimited with a ballpoint pen, the central area was cut out 0.5 cm in diameter (arrow), resulting in 0.25 cm^2^ of cotton fabric fragment, which was placed in 1.5mL eppendorf tubes for DNA extraction. All cotton fabric presented yellowish dots along the entire length of the cotton fabric.


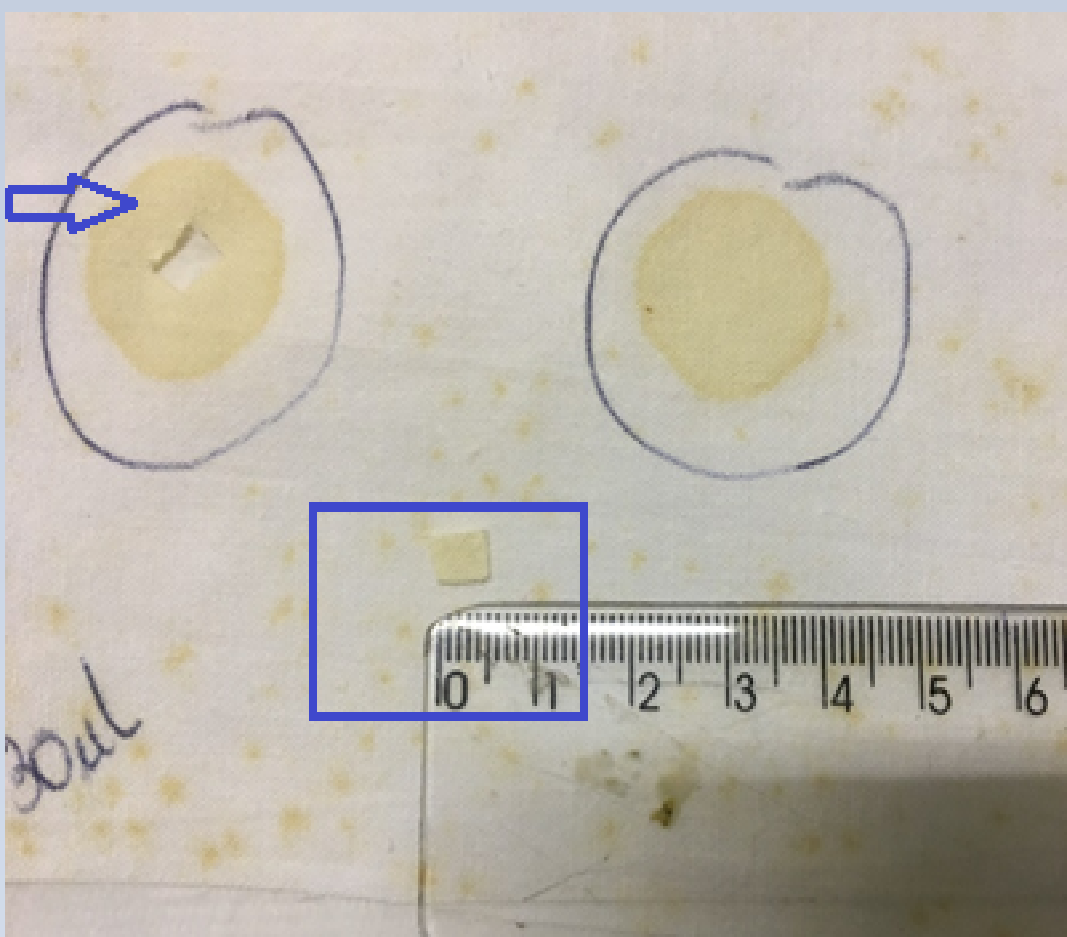

Supplement: Supplementary file 1 — Supplementary Information 1. [file 41598_2021_87937_MOESM1_ESM.docx]
