# Supplementary material for: Systematic STR analysis of old post-vasectomy seminal fluid stains to examine evidence stored for 16 years
Source: Sci Rep. 2021 Apr 26;11:8918. doi: 10.1038/s41598-021-87937-x (PMC8076208; doi:10.1038/s41598-021-87937-x)
Supplement: Supplementary file 2 — Supplementary Information 2. [file 41598_2021_87937_MOESM2_ESM.docx]

**Supplementary information**

**Systematic STR analysis of old post-vasectomy seminal fluid stains to examine evidence stored for 16 years**

Julianna Kesselring Romero^1^, Eloisa Auler Bittencourt^1,2^, José Arnaldo Soares-Vieira^3^, Ana Claudia Pacheco^4^, Alexandre Learth Soares^4^, Edna Sadayo Miazato Iwamura ^1^*

^1^ Laboratório de Patologia Molecular, Departamento de Patologia - Escola Paulista de Medicina /Universidade Federal de São Paulo (EPM/UNIFESP), SP, Brazil; ^2^ Academia de Polícia de São Paulo (ACADEPOL), SP, Brazil; ^3^ Departamento de Medicina Legal, Ética Médica, Medicina Social e do Trabalho- Faculdade de Medicina da Universidade São Paulo (USP), SP, Brazil; ^4^  Instituto de Criminalística-Superintendência da Polícia Técnico-Científica do Estado de São Paulo (SPTC SP), SP, Brazil

* Corresponding author: Edna Sadayo Miazato Iwamura, phone +55 11 5576 4848 ext 1386, e-mail: edna.iwamura@unifesp.com

Departamento de Patologia, Escola Paulista de Medicina/Universidade Federal de São Paulo (EPM/UNIFESP), Rua Botucatu 740, Edifício Lemos Torres. Vila Clementino- CEP 04023-62, São Paulo, SP- Brazil.

**Supplementary Graphs-** Correlation between DNA concentration pre- and post- vasectomy (stains on cotton fabric)

The DNA concentration measurements, obtained from the stored cotton fabric stains, were obtained from the supplementary table (Supplementary table 1 Quantification and Degradation).

Dispersion plots of DNA measurements pre- and post vasectomy were constructed in two ways: with the original measurements (ng / µL) (Graph 8a) and with the logarithms of the measurements - log (ng / µL) (Graph 8b) for facilitating viewing.

The correlation was calculated using Spearman's correlation coefficient ρ (Conover, 1999), as in the previous case (Graphs 6 and 7). The Spearman correlation between the 90 pairs of measurements collected before and after vasectomy is ρ = -0.05 (p-value = 0.658), that is, the measurements do not present a significant correlation, unlike the measurements of the “fresh, *in natura*” samples.


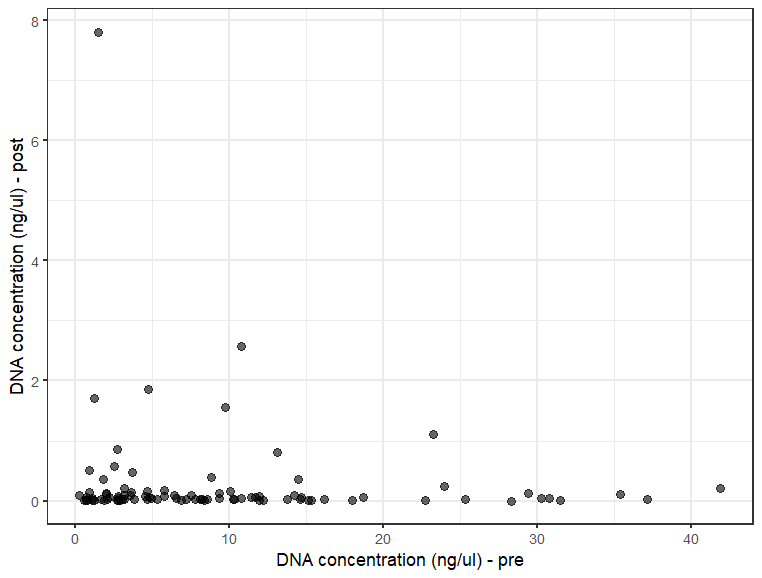


**Graph S1a**: Relationship between semen DNA concentrations (ng / µL) pre- and post-vasectomy (measure Y).


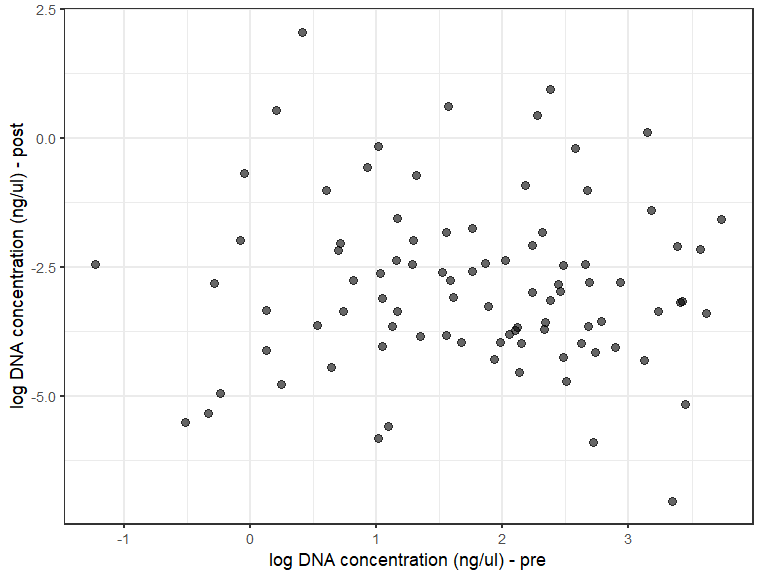


**Graph S1b**: Relationship between the logarithms of DNA concentrations (ng / µL) pre- and post- vasectomy (measure Y).

## 
