# Supplementary material for: Systematic STR analysis of old post-vasectomy seminal fluid stains to examine evidence stored for 16 years
Source: Sci Rep. 2021 Apr 26;11:8918. doi: 10.1038/s41598-021-87937-x (PMC8076208; doi:10.1038/s41598-021-87937-x)
Supplement: Supplementary file 3 — Supplementary Information 3. [file 41598_2021_87937_MOESM3_ESM.docx]

**Supplementary information**

**Systematic STR analysis of old post-vasectomy seminal fluid stains to examine evidence stored for 16 years**

Julianna Kesselring Romero^1^, Eloisa Auler Bittencourt^1,2^, José Arnaldo Soares-Vieira^3^, Ana Claudia Pacheco^4^, Alexandre Learth Soares^4^, Edna Sadayo Miazato Iwamura ^1^*

^1^ Laboratório de Patologia Molecular, Departamento de Patologia - Escola Paulista de Medicina /Universidade Federal de São Paulo (EPM/UNIFESP), SP, Brazil; ^2^ Academia de Polícia de São Paulo (ACADEPOL), SP, Brazil; ^3^ Departamento de Medicina Legal, Ética Médica, Medicina Social e do Trabalho- Faculdade de Medicina da Universidade São Paulo (USP), SP, Brazil; ^4^  Instituto de Criminalística-Superintendência da Polícia Técnico-Científica do Estado de São Paulo (SPTC SP), SP, Brazil

* Corresponding author : Edna Sadayo Miazato Iwamura, phone +55 11 5576 4848 ext 1386, e-mail: edna.iwamura@unifesp.com

Departamento de Patologia, Escola Paulista de Medicina/Universidade Federal de São Paulo (EPM/UNIFESP), Rua Botucatu 740, Edifício Lemos Torres. Vila Clementino- CEP 04023-62, São Paulo, SP- Brazil.

**Supplementary table S1**. Results of quantification and degradation index (Small/Large) evaluation of pre-vasectomy semen and post-vasectomy seminal fluid stain samples of 90 individuals. Blood samples DNA were extracted in natura as control. Analysis of the volume (μl) used in each of the amplification systems (Fusion 6C and Powerplex Y23) after dilution. In yellow. the different volumes used in the two systems.

| sample | pre-vasectomy semen | Blood | post-vasectomy seminal fluid |
| --- | --- | --- | --- |
|  | | | |
| 2364 | | | |
| Large | 0.3008004 | 458.8491 | 0.000915 |
| Small | 1.408127 | 1561.864 | 0.010016 |
| Y | 1.897953 | 1274.321 | 0.011681 |
| Degradation index | 4.681216 | 3.403872 | 10.94259 |
| FUSION 6C dilution | * | 1:1000 | * |
| Volume | 0.5 | 0.5 | 15.0 |
| PPY dilution | * | 1:1000 | * |
| Volume | 0.5 | 0.5 | 15.0 |
|  | | | |
| 2365 | | | |
| Large | 0.383272 | 247.7594 | 0.801411 |
| Small | 1.391476 | 429.8826 | 5.549853 |
| Y | 1.512199 | 430.7607 | 7.803166 |
| Degradation index | 3.630516 | 1.735081 | 6.925105 |
| FUSION 6C dilution | * | 1:1000 | * |
| Volume | 0.5 | 1.0 | 15.0 |
| PPY dilution | * | 1:1000 | 1:10 |
| Volume | 0.5 | 1.0 | 1.0 |
|  | | | |
| 2367 | | | |
| Large | 2.820908 | 353.942 | 0.01398 |
| Small | 6.203443 | 703.7764 | 0.058774 |
| Y | 7.5468 | 737.1506 | 0.093462 |
| Degradation index | 2.199094 | 1.988394 | 4.204216 |
| FUSION 6C dilution | 1:10 | 1:1000 | * |
| Volume | 1.0 | 1.0 | 10.0 |
| PPY dilution | 1:10 | 1:1000 | * |
| Volume | 1.0 | 1.0 | 5.0 |
|  | | | |
| 2368 | | | |
| Large | 3.442957 | 261.0581 | 0.015691 |
| Small | 9.738446 | 416.8874 | 0.13052 |
| Y | 10.08399 | 393.7225 | 0.16056 |
| Degradation index | 2.828512 | 1.596914 | 8.318149 |
| FUSION 6C dilution | 1:20 | 1:1000 | * |
| Volume | 1.0 | 1.0 | 4 |
| PPY dilution | 1:20 | 1:1000 | * |
| Volume | 1.0 | 1.0 | 4 |
|  | | | |
| 2369 | | | |
| Large | 10.49682 | 317.8262 | 0.006538 |
| Small | 17.5845 | 485.031 | 0.01352 |
| Y | 15.35994 | 381.5282 | 0.015648 |
| Degradation index | 1.675221 | 1.526089 | 2.067916 |
| FUSION 6C dilution | 1:20 | 1:1000 | * |
| Volume | 1.0 | 1.0 | 15.0 |
| PPY dilution | 1:20 | 1:1000 | * |
| Volume | 1.0 | 1.0 | 15.0 |
|  | | | |
| 2405 | | | |
| Large | 1.355955 | 304.461 | 0.006467 |
| Small | 3.020021 | 433.8332 | 0.022843 |
| Y | 3.202495 | 498.1188 | 0.03479 |
| Degradation index | 2.227228 | 1.439102 | 3.532275 |
| FUSION 6C dilution | 1:10 | 1:1000 | * |
| Volume | 2.0 | 1.0 | 15.0 |
| PPY dilution | 1:10 | 1:1000 | * |
| Volume | 2.0 | 1.0 | 15.0 |
|  | | | |
| 2406 | | | |
| Large | 6.17909 | 251.1211 | 0.007006 |

| Small | 10.84086 | 404.0522 | 0.024284 |
| --- | --- | --- | --- |
| Y | 10.29672 | 348.2942 | 0.024794 |
| Degradation index | 1.754443 | 1.608994 | 3.466107 |
| FUSION 6C dilution | 1:20 | 1:1000 | * |
| Volume | 1.0 | 1.0 | 15.0 |
| PPY dilution | 1:20 | 1:1000 | * |
| Volume | 1.0 | 1.0 | 15.0 |
|  | | | |
| 2407 | | | |
| Large | 2.354486 | 231.5976 | 0.0077 |
| Small | 4.155243 | 348.739 | 0.023073 |
| Y | 3.83687 | 358.8153 | 0.021695 |
| Degradation index | 1.76482 | 1.505797 | 2.996578 |
| FUSION 6C dilution | 1:10 | 1:500 | * |
| Volume | 1.0 | 1.0 | 15.0 |
| PPY dilution | 1:10 | 1:500 | * |
| Volume | 1.0 | 1.0 | 15.0 |
|  | | | |
| 2414 | | | |
| Large | 4.180526 | 383.8899 | 0.003704 |
| Small | 5.629316 | 542.6386 | 0.011561 |
| Y | 6.902949 | 754.1929 | 0.013699 |
| Degradation index | 1.346557 | 1.413527 | 3.120901 |
| FUSION 6C dilution | 1:10 | 1:1000 | * |
| Volume | 1.0 | 1.0 | 15.0 |
| PPY dilution | 1:10 | 1:1000 | * |
| Volume | 1.0 | 1.0 | 15.0 |
|  | | | |
| 2415 | | | |
| Large | 14.03855 | 192.4537 | 0.01044 |
| Small | 27.36546 | 251.0183 | 0.037653 |
| Y | 30.28772 | 286.0445 | 0.041307 |
| Degradation index | 1.949308 | 1.304305 | 3.6067 |
| FUSION 6C dilution | 1:40 | 1:1000 | * |
| Volume | 1.0 | 2.0 | 15.0 |
| PPY dilution | 1:40 | 1:1000 | * |
| Volume | 1.0 | 2.0 | 15.0 |
|  | | | |
| 2421 | | | |
| Large | 7.089983 | 540.1299 | 0.094214 |
| Small | 13.05529 | 1242.719 | 0.349489 |
| Y | 14.47153 | 1546.844 | 0.365048 |
| Degradation index | 1.841372 | 2.300777 | 3.709533 |
| FUSION 6C dilution | 1:20 | 1:1000 | * |
| Volume | 1.0 | 0.5 | 2.0 |
| PPY dilution | 1:20 | 1:1000 | * |
| Volume | 1.0 | 0.5 | 2.0 |
|  | | | |
| 2423 | | | |
| Large | 16.30426 | 363.1234 | 0.029364 |
| Small | 29.40909 | 624.4808 | 0.132992 |
| Y | 29.44355 | 635.531 | 0.121896 |
| Degradation index | 1.803768 | 1.857891 | 4.529027 |
| FUSION 6C dilution | 1:40 | 1:1000 | * |
| Volume | 1.0 | 1.0 | 4.0 |
| PPY dilution | 1:40 | 1:1000 | * |
| Volume | 1.0 | 1.0 | 4.0 |
|  | | | |
| 2424 | | | |
| Large | 7.746307 | 163.7818 | 0.000831 |
| Small | 21.34444 | 322.8303 | 0.008801 |
| Y | 28.34117 | 299.9661 | 0.000885 |
| Degradation index | 2.755434 | 1.9711 | 10.59278 |
| FUSION 6C dilution | 1:30 | 1:500 | * |
| Volume | 1.0 | 1.0 | 15.0 |
| PPY dilution | 1:30 | 1:500 | * |
| Volume | 1.0 | 1.0 | 15.0 |
|  | | | |
| 2425 | | | |
| Large | 10.17962 | 428.6889 | 0.005804 |
| Small | 15.13065 | 725.5923 | 0.01711 |
| Y | 18.0251 | 663.8185 | 0.017291 |

| Degradation index | 1.486366 | 1.692585 | 2.948061 |
| --- | --- | --- | --- |
| FUSION 6C dilution | 1:20 | 1:1000 | * |
| Volume | 1.0 | 1.0 | 15.0 |
| PPY dilution | 1:20 | 1:1000 | * |
| Volume | 1.0 | 1.0 | 15.0 |
|  | | | |
| 2442 | | | |
| Large | 1.151878 | 577.2828 | 0.163814 |
| Small | 1.375261 | 804.0312 | 0.411456 |
| Y | 1.828315 | 989.2693 | 0.36691 |
| Degradation index | 1.19393 | 1.392786 | 2.511733 |
| FUSION 6C dilution | * | 1:1000 | * |
| Volume | 0.5 | 1.0 | 1.0 |
| PPY dilution | * | 1:1000 | * |
| Volume | 0.5 | 1.0 | 2.0 |
|  | | | |
| 2445 | | | |
| Large | 3.505577 | 276.2287 | 0.050851 |
| Small | 5.010527 | 446.0568 | 0.157982 |
| Y | 5.807222 | 545.6002 | 0.175971 |
| Degradation index | 1.429302 | 1.61481 | 3.106751 |
| FUSION 6C dilution | 1:10 | 1:1000 | * |
| Volume | 1.0 | 1.0 | 4.0 |
| PPY dilution | 1:10 | 1:1000 | * |
| Volume | 1.0 | 1.0 | 4.0 |
|  | | | |
| 2446 | | | |
| Large | 2.15182 | 346.2787 | 0.036424 |
| Small | 2.864622 | 504.6118 | 0.096104 |
| Y | 2.807547 | 490.6862 | 0.072838 |
| Degradation index | 1.331255 | 1.457242 | 2.63851 |
| FUSION 6C dilution | 1:10 | 1:1000 | * |
| Volume | 2.0 | 1.0 | 5.0 |
| PPY dilution | 1:10 | 1:1000 | * |
| Volume | 2.0 | 1.0 | 6.0 |
|  | | | |
| 2447 | | | |
| Large | 4.621156 | 334.3868 | 0.017145 |
| Small | 8.898209 | 510.2497 | 0.056812 |
| Y | 9.357647 | 531.5092 | 0.050155 |
| Degradation index | 1.925537 | 1.525926 | 3.313703 |
| FUSION 6C dilution | 1:20 | 1:1000 | * |
| Volume | 1.0 | 1.0 | 10.0 |
| PPY dilution | 1:20 | 1:1000 | * |
| Volume | 1.0 |  | 10.0 |
|  | | | |
| 2450 | | | |
| Large | 1.207147 | 297.642 | 0.011282 |
| Small | 1.354944 | 381.7089 | 0.020504 |
| Y | 1.691994 | 501.2368 | 0.02659 |
| Degradation index | 1.122434 | 1.282443 | 1.81714 |
| FUSION 6C dilution | * | 1:500 | * |
| Volume | 0.5 | 1.0 | 15.0 |
| PPY dilution | * | 1:500 | * |
| Volume | 0.5 | 1.0 | 15.0 |
|  | | | |
| 2451 | | | |
| Large | 17.50217 | 301.5192 | 0.041122 |
| Small | 25.79692 | 403.4706 | 0.112347 |
| Y | 35.39189 | 496.9131 | 0.115754 |
| Degradation index | 1.473927 | 1.338126 | 2.73206 |
| FUSION 6C dilution | 1:40 | 1:1000 | * |
| Volume | 1.0 | 1.0 | 4.0 |
| PPY dilution | 1:40 | 1:1000 | * |
| Volume | 1.0 | 1.0 | 4.0 |
|  | | | |
| 2452 | | | |
| Large | 4.74805 | 266.2347 | 0.005835 |
| Small | 7.309695 | 412.7843 | 0.018864 |
| Y | 8.571043 | 476.3606 | 0.018927 |
| Degradation index | 1.539515 | 1.550453 | 3.232973 |
| FUSION 6C dilution | 1:10 | 1:1000 | * |
| Volume | 1.0 | 1.0 | 15.0 |
| PPY dilution | 1:20 | 1:1000 | * |
| Volume | 1.0 | 1.0 | 15.0 |
|  | | | |

| 2453 | | | |
| --- | --- | --- | --- |
| Large | 2.815542 | 396.2544 | 0.005176 |
| Small | 4.888629 | 726.1306 | 0.019156 |
| Y | 4.731526 | 506.7456 | 0.021776 |
| Degradation index | 1.736301 | 1.832486 | 3.701261 |
| FUSION 6C dilution | 1:10 | 1:1000 | * |
| Volume | 1.0 | 1.0 | 15.0 |
| PPY dilution | 1:10 | 1:1000 | * |
| Volume | 1.0 | 1.0 | 15.0 |
|  | | | |
| 2454 | | | |
| Large | 27.69556 | 324.7637 | 0.012464 |
| Small | 38.23151 | 510.5429 | 0.034216 |
| Y | 37.15071 | 495.9763 | 0.033529 |
| Degradation index | 1.38042 | 1.572044 | 2.745166 |
| FUSION 6C dilution | 1:40 | 1:1000 | * |
| Volume | 1.0 | 1.0 | 15.0 |
| PPY dilution | 1:40 | 1:1000 | * |
| Volume | 1.0 | 1.0 | 15.0 |
|  | | | |
| 2455 | | | |
| Large | 6.641873 | 170.1213 | 0.710403 |
| Small | 9.029415 | 244.6472 | 1.545599 |
| Y | 9.750607 | 240.8806 | 1.563578 |
| Degradation index | 1.359468 | 1.438075 | 2.175666 |
| FUSION 6C dilution | 1:20 | 1:500 | * |
| Volume | 1.0 | 2.0 | 0.5 |
| PPY dilution | 1:20 | 1:500 | * |
| Volume | 1.0 | 2.0 | 0.5 |
|  | | | |
| 2456 | | | |
| Large | 6.816129 | 217.6687 | 0.004789 |
| Small | 11.14058 | 388.2065 | 0.010223 |
| Y | 12.25589 | 347.7951 | 0.008963 |
| Degradation index | 1.634444 | 1.783475 | 2.134794 |
| FUSION 6C dilution | 1:20 | 1:500 | * |
| Volume | 1.0 | 1.0 | 15.0 |
| PPY dilution | 1:20 | 1:500 | * |
| Volume | 1.0 | 1.0 | 15.0 |
|  | | | |
| 2457 | | | |
| Large | 9.92621 | 341.8446 | 0.041456 |
| Small | 15.91116 | 622.4179 | 0.056878 |
| Y | 18.7222 | 619.783 | 0.060642 |
| Degradation index | 1.602944 | 1.820763 | 1.372105 |
| FUSION 6C dilution | 1:30 | 1:1000 | * |
| Volume | 1.0 | 1.0 | 10.0 |
| PPY dilution | 1:30 | 1:1000 | * |
| Volume | 1.0 | 1.0 | 10.0 |
|  | | | |
| 2458 | | | |
| Large | 0.560357 | 268.8412 | 0.001515 |
| Small | 0.72616 | 488.512 | 0.007334 |
| Y | 0.784787 | 490.1525 | 0.007051 |
| Degradation index | 1.295887 | 1.817102 | 2.915864 |
| FUSION 6C dilution | * | 1:1000 | * |
| Volume | 1.0 | 1.0 | 15.0 |
| PPY dilution | * | 1:1000 | * |
| Volume | 1.0 | 1.0 | 15.0 |
|  | | | |
| 2459 | | | |
| Large | 2.244409 | 250.1194 | 0.00266 |
| Small | 2.907554 | 409.8993 | 0.005614 |
| Y | 2.97106 | 398.4968 | 0.00373 |
| Degradation index | 1.295465 | 1.638814 | 2.110356 |
| FUSION 6C dilution | 1:10 | 1:1000 | * |
| Volume | 2.0 | 1.0 | 15.0 |
| PPY dilution | 1:10 | 1:1000 | * |
| Volume | 2.0 | 1.0 | 15.0 |
|  | | | |
| 2460 | | | |
| Large | 2.190265 | 257.646 | 0.001615 |
| Small | 4.077651 | 481.0988 | 0.004782 |
| Y | 2.763091 | 314.595 | 0.002987 |
| Degradation index | 1.861715 | 1.867286 | 2.961055 |

| FUSION 6C dilution | 1:10 | 1:1000 | * |
| --- | --- | --- | --- |
| Volume | 1.0 | 1.0 | 15.0 |
| PPY dilution | 1:10 | 1:1000 | * |
| Volume | 2.0 | 1.0 | 15.0 |
|  | | | |
| 2461 | | | |
| Large | 7.372561 | 197.3265 | 0.011157 |
| Small | 9.289648 | 338.8138 | 0.026697 |
| Y | 10.33851 | 344.0496 | 0.028388 |
| Degradation index | 1.26003 | 1.717021 | 2.392843 |
| FUSION 6C dilution | 1:20 | 1:500 | * |
| Volume | 1.0 | 1.0 | 15.0 |
| PPY dilution | 1:20 | 1:500 | * |
| Volume | 1.0 | 1.0 | 15.0 |
|  | | | |
| 2471 | | | |
| Large | 7.246141 | 316.4775 | 0.036041 |
| Small | 8.721444 | 456.4803 | 0.049199 |
| Y | 11.44416 | 511.7177 | 0.058965 |
| Degradation index | 1.203598 | 1.442378 | 1.365088 |
| FUSION 6C dilution | 1:20 | 1:1000 | * |
| Volume | 1.0 | 1.0 | 10.0 |
| PPY dilution | 1:20 | 1:1000 | * |
| Volume | 1.0 | 1.0 | 10.0 |
|  | | | |
| 2472 | | | |
| Large | 3.304858 | 385.231 | 0.081085 |
| Small | 4.054952 | 595.9758 | 0.170161 |
| Y | 4.709638 | 610.494 | 0.16145 |
| Degradation index | 1.226967 | 1.547061 | 2.098551 |
| FUSION 6C dilution | 1:10 | 1:1000 | * |
| Volume | 1.0 | 1.0 | 4.0 |
| PPY dilution | 1:10 | 1:1000 | * |
| Volume | 1.0 | 1.0 | 4.0 |
|  | | | |
| 2473 | | | |
| Large | 0.468385 | 229.2087 | 0.001001 |
| Small | 0.531838 | 320.701 | 0.002771 |
| Y | 0.593942 | 364.3174 | 0.004072 |
| Degradation index | 1.135473 | 1.399166 | 2.766991 |
| FUSION 6C dilution | * | 1:500 | * |
| Volume | 1.0 | 1.0 | 15.0 |
| PPY dilution | * | 1:500 | * |
| Volume | 1.0 | 1.0 | 15.0 |
|  | | | |
| 2475 | | | |
| Large | 0.741451 | 221.6652 | 0.003165 |
| Small | 1.006851 | 325.815 | 0.012728 |
| Y | 1.128976 | 375.5749 | 0.016502 |
| Degradation index | 1.357947 | 1.469852 | 4.021864 |
| FUSION 6C dilution | * | 1:500 | * |
| Volume | 0.5 | 1.0 | 15.0 |
| PPY dilution | * | 1:500 | * |
| Volume | 0.5 | 1.0 | 15.0 |
|  | | | |
| 2476 | | | |
| Large | 0.45666 | 304.5081 | 0.002072 |
| Small | 0.564784 | 459.1367 | 0.007473 |
| Y | 0.717049 | 556.1801 | 0.004807 |
| Degradation index | 1.23677 | 1.507798 | 3.605961 |
| FUSION 6C dilution | * | 1:1000 | * |
| Volume | 1.0 | 1.0 | 15.0 |
| PPY dilution | * | 1:1000 | * |
| Volume | 1.0 | 1.0 | 15.0 |
|  | | | |
| 2477 | | | |
| Large | 2.465604 | 384.4396 | 0.049883 |
| Small | 3.385746 | 708.9366 | 0.141793 |
| Y | 3.639696 | 743.1738 | 0.138241 |
| Degradation index | 1.373191 | 1.844078 | 2.842479 |
| FUSION 6C dilution | 1:10 | 1:1000 | * |
| Volume | 2.0 | 1.0 | 4.0 |
| PPY dilution | 1:10 | 1:1000 | * |
| Volume | 2.0 | 1.0 | 4.0 |
|  | | | |
| 2478 | | | |
| Large | 4.125858 | 310.7501 | 0.025934 |
| Small | 7.394613 | 600.2087 | 0.110135 |
| Y | 9.37237 | 730.6414 | 0.124233 |
| Degradation index | 1.792261 | 1.931484 | 4.246812 |
| FUSION 6C dilution | 1:10 | 1:1000 | * |
| Volume | 1.0 | 1.0 | 5.0 |
| PPY dilution | 1:10 | 1:1000 | * |
| Volume | 1.0 | 1.0 | 5.0 |
|  | | | |
| 2480 | | | |
| Large | 0.704788 | 267.3846 | 0.096624 |
| Small | 0.868721 | 453.4335 | 0.521871 |
| Y | 0.952859 | 483.1782 | 0.510451 |
| Degradation index | 1.232599 | 1.69581 | 5.40105 |
| FUSION 6C dilution | * | 1:1000 | * |
| Volume | 0.5 | 1.0 | 1.0 |
| PPY dilution | * | 1:1000 | * |
| Volume | 0.5 | 1.0 | 1.0 |
|  | | | |
| 2488 | | | |
| Large | 7.313132 | 282.1434 | 0.040721 |
| Small | 13.47014 | 458.6145 | 0.064883 |
| Y | 14.72081 | 430.9605 | 0.061679 |
| Degradation index | 1.841911 | 1.625466 | 1.593356 |
| FUSION 6C dilution | 1:20 | 1:1000 | * |
| Volume | 1.0 | 1.0 | 10.0 |
| PPY dilution | 1:20 | 1:1000 | * |
| Volume | 1.0 | 1.0 | 10.0 |
|  | | | |
| 2489 | | | |
| Large | 2.929608 | 104.9333 | 0.013812 |
| Small | 4.553629 | 192.2243 | 0.037657 |
| Y | 4.98097 | 192.8106 | 0.046022 |
| Degradation index | 1.554348 | 1.831871 | 2.72646 |
| FUSION 6C dilution | 1:10 | 1:500 | * |
| Volume | 1.0 | 3.0 | 15.0 |
| PPY dilution | 1:10 | 1:500 | * |
| Volume | 1.0 | 3.0 | 15.0 |
|  | | | |
| 2490 | | | |
| Large | 2.016864 | 340.1974 | 0.157548 |
| Small | 3.445256 | 628.2285 | 0.428527 |
| Y | 3.707824 | 698.5079 | 0.484099 |
| Degradation index | 1.708225 | 1.846659 | 2.719969 |
| FUSION 6C dilution | 1:10 | 1:1000 | * |
| Volume | 2.0 | 1.0 | 1.0 |
| PPY dilution | 1:10 | 1:1000 | * |
| Volume | 2.0 | 1.0 | 1.0 |
|  | | | |
| 2491 | | | |
| Large | 13.59988 | 305.1395 | 0.001976 |
| Small | 23.63145 | 435.9596 | 0.005171 |
| Y | 31.50034 | 489.4253 | 0.005709 |
| Degradation index | 1.737622 | 1.428722 | 2.617065 |
| FUSION 6C dilution | 1:30 | 1:1000 | * |
| Volume | 1.0 | 1.0 | 15.0 |
| PPY dilution | 1:30 | 1:1000 | * |
| Volume | 1.0 | 1.0 | 15.0 |
|  | | | |
| 2492 | | | |
| Large | 8.087072 | 228.1081 | 0.009937 |
| Small | 12.3016 | 386.853 | 0.032743 |
| Y | 16.19263 | 440.8955 | 0.028874 |
| Degradation index | 1.521144 | 1.69592 | 3.295153 |
| FUSION 6C dilution | 1:20 | 1:500 | * |
| Volume | 1.0 | 1.0 | 15.0 |
| PPY dilution | 1:20 | 1:500 | * |
| Volume | 1.0 | 1.0 | 15.0 |
|  | | | |

| 2496 | | | |
| --- | --- | --- | --- |
| Large | 1.781215 | 249.0604 | 0.007671 |
| Small | 2.64646 | 437.3042 | 0.015953 |
| Y | 2.836679 | 461.6232 | 0.017634 |
| Degradation index | 1.485761 | 1.755816 | 2.079538 |
| FUSION 6C dilution | 1:10 | 1:1000 | * |
| Volume | 2.0 | 1.0 | 15.0 |
| PPY dilution | 1:10 | 1:1000 | * |
| Volume | 2.0 | 1.0 | 15.0 |
|  | | | |
| 2498 | | | |
| Large | 4.969479 | 262.1511 | 0.006457 |
| Small | 6.101767 | 359.5356 | 0.017858 |
| Y | 7.789943 | 399.198 | 0.022348 |
| Degradation index | 1.227848 | 1.371482 | 2.765738 |
| FUSION 6C dilution | 1:10 | 1:500 | * |
| Volume | 1.0 | 1.0 | 15.0 |
| PPY dilution | 1:10 | 1:500 | * |
| Volume | 1.0 | 1.0 | 15.0 |
|  | | | |
| 2499 | | | |
| Large | 11.51194 | 385.9034 | 0.005695 |
| Small | 15.62075 | 435.0949 | 0.012732 |
| Y | 22.76345 | 522.0738 | 0.013473 |
| Degradation index | 1.356918 | 1.521825 | 2.235613 |
| FUSION 6C dilution | 1:20 | 1:1000 | * |
| Volume | 1.0 | 1.0 | 15.0 |
| PPY dilution | 1:20 | 1:1000 | * |
| Volume | 0.5 | 1.0 | 15.0 |
|  | | | |
| 2500 | | | |
| Large | 8.85283 | 207.5445 | 0.006314 |
| Small | 8.853182 | 244.7029 | 0.27223 |
| Y | 11.68755 | 302.2404 | 0.051808 |
| Degradation index | 1.00004 | 1.179038 | 43.11692 |
| FUSION 6C dilution | 1:20 | 1:500 | * |
| Volume | 1.0 | 1.0 | 2.0 |
| PPY dilution | 1:20 | 1:500 | * |
| Volume | 1.0 | 1.0 | 10.0 |
|  | | | |
| 2501 | | | |
| Large | 9.180723 | 146.9713 | 0.025725 |
| Small | 10.61035 | 232.9803 | 0.043667 |
| Y | 10.77476 | 227.7254 | 0.043246 |
| Degradation index | 1.15572 | 1.58521 | 1.697433 |
| FUSION 6C dilution | 1:20 | 1:1000 | * |
| Volume | 1.0 | 1.0 | 10.0 |
| PPY dilution | 1:20 | 1:1000 | * |
| Volume | 1.0 | 1.0 | 10.0 |
|  | | | |
| 2502 | | | |
| Large | 3.039148 | 253.6897 | 0.04308 |
| Small | 4.240053 | 504.1606 | 0.086363 |
| Y | 4.55623 | 446.172 | 0.074046 |
| Degradation index | 1.395145 | 1.987241 | 2.004698 |
| FUSION 6C dilution | 1:10 | 1:1000 | * |
| Volume | 1.0 | 1.0 | 5.0 |
| PPY dilution | 1:10 | 1:1000 | * |
| Volume | 1.0 | 1.0 | 5.0 |
|  | | | |
| 2503 | | | |
| Large | 3.308237 | 217.375 | 0.010074 |
| Small | 5.547053 | 338.7126 | 0.034142 |
| Y | 6.565276 | 347.2557 | 0.038721 |
| Degradation index | 1.67674 | 1.558195 | 3.389077 |
| FUSION 6C dilution | 1:10 | 1:500 | * |
| Volume | 1.0 | 1.0 | 15.0 |
| PPY dilution | 1:10 | 1:500 | * |
| Volume | 1.0 | 1.0 | 15.0 |
|  | | | |
| 2505 | | | |
| Large | 3.275966 | 187.2979 | 0.009453 |

| Small | 4.682297 | 293.2931 | 0.021134 |
| --- | --- | --- | --- |
| Y | 5.333101 | 294.6572 | 0.01913 |
| Degradation index | 1.429287 | 1.565918 | 2.23568 |
| FUSION 6C dilution | 1:10 | 1:500 | * |
| Volume | 1.0 | 1.0 | 15.0 |
| PPY dilution | 1:10 | 1:500 | * |
| Volume | 1.0 | 1.0 | 15.0 |
|  | | | |
| 2512 | | | |
| Large | 3.526154 | 262.9453 | 0.032642 |
| Small | 4.440938 | 428.8702 | 0.065463 |
| Y | 5.805342 | 487.913 | 0.07509 |
| Degradation index | 1.259428 | 1.631025 | 2.005509 |
| FUSION 6C dilution | 1:10 | 1:1000 | * |
| Volume | 1.0 | 1.0 | 10.0 |
| PPY dilution | 1:10 | 1:1000 | * |
| Volume | 1.0 |  | 10.0 |
|  | | | |
| 2514 | | | |
| Large | 21.63888 | 232.5009 | 0.188293 |
| Small | 24.90614 | 390.2336 | 0.307029 |
| Y | 24.00933 | 328.2924 | 0.247821 |
| Degradation index | 1.15099 | 1.678478 | 1.630596 |
| FUSION 6C dilution | 1:40 | 1:500 | * |
| Volume | 1.0 | 1.0 | 2.0 |
| PPY dilution | 1:40 | 1:500 | * |
| Volume | 1.0 | 1.0 | 2.0 |
|  | | | |
| 2515 | | | |
| Large | 11.16212 | 270.0929 | 0.063555 |
| Small | 11.01187 | 423.675 | 0.086784 |
| Y | 11.97249 | 397.4171 | 0.084343 |
| Degradation index | 0.986539 | 1.568627 | 1.365494 |
| FUSION 6C dilution | 1:20 | 1:1000 | * |
| Volume | 1.0 | 1.0 | 5.0 |
| PPY dilution | 1:20 | 1:1000 | * |
| Volume | 1.0 | 1.0 | 5.0 |
|  | | | |
| 2517 | | | |
| Large | 5.066702 | 391.1339 | 0.009194 |
| Small | 8.189911 | 787.5563 | 0.029296 |
| Y | 8.133205 | 669.8122 | 0.024121 |
| Degradation index | 1.616419 | 2.013521 | 3.186441 |
| FUSION 6C dilution | 1:20 | 1:1000 | * |
| Volume | 1.0 | 1.0 | 15.0 |
| PPY dilution | 1:20 | 1:1000 | * |
| Volume | 1.0 | 1.0 | 15.0 |
|  | | | |
| 2518 | | | |
| Large | 2.338813 | 229.1171 | 0.012493 |
| Small | 2.627938 | 384.6437 | 0.04127 |
| Y | 2.842815 | 401.9868 | 0.045299 |
| Degradation index | 1.126321 | 1.678801 | 3.303439 |
| FUSION 6C dilution | 1:10 | 1:500 | * |
| Volume | 2.0 | 1.0 | 15.0 |
| PPY dilution | 1:10 | 1:500 | * |
| Volume | 2.0 | 1.0 | 15.0 |
|  | | | |
| 2548 | | | |
| Large | 1.417021 | 380.2294 | 0.054472 |
| Small | 1.474906 | 548.595 | 0.094277 |
| Y | 2.010349 | 684.0649 | 0.114827 |
| Degradation index | 1.048247 | 1.4428 | 1.730754 |
| FUSION 6C dilution | * | 1:1000 | * |
| Volume | 0.5 | 1.0 | 5.0 |
| PPY dilution | * | 1:1000 | * |
| Volume | 0.5 | 1.0 | 5.0 |
|  | | | |
| 2550 | | | |
| Large | 10.0607 | 454.704 | 0.010247 |
| Small | 11.34316 | 727.9312 | 0.023797 |
| Y | 14.6228 | 909.543 | 0.025907 |

| Degradation index | 1.127472 | 1.60089 | 2.322416 |
| --- | --- | --- | --- |
| FUSION 6C dilution | 1:20 | 1:1000 | * |
| Volume | 1.0 | 1.0 | 15.0 |
| PPY dilution | 1:20 | 1:1000 | * |
| Volume | 1.0 | 1.0 | 15.0 |
|  | | | |
| 2551 | | | |
| Large | 3.333037 | 411.064 | 0.770505 |
| Small | 2.922602 | 653.8621 | 1.378899 |
| Y | 4.787824 | 918.2266 | 1.863212 |
| Degradation index | 0.876859 | 1.590658 | 1.789604 |
| FUSION 6C dilution | 1:10 | 1:1000 | * |
| Volume | 2.0 | 1.0 | 0.5 |
| PPY dilution | 1:10 | 1:1000 | * |
| Volume | 1.0 | 1.0 | 0.5 |
|  | | | |
| 2552 | | | |
| Large | 5.801245 | 276.1469 | 0.004039 |
| Small | 7.408713 | 375.1851 | 0.10927 |
| Y | 11.96224 | 513.1579 | 0.014194 |
| Degradation index | 1.27709 | 1.358643 | 2.705288 |
| FUSION 6C dilution | 1:10 | 1:500 | * |
| Volume | 1.0 | 1.0 | 15.0 |
| PPY dilution | 1:10 | 1:500 | * |
| Volume | 1.0 | 1.0 | 15.0 |
|  | | | |
| 2553 | | | |
| Large | 1.08549 | 378.1192 | 0.022756 |
| Small | 2.105726 | 642.4724 | 0.15366 |
| Y | 2.03169 | 569.6768 | 0.13056 |
| Degradation index | 1.939886 | 1.699126 | 6.752624 |
| FUSION 6C dilution | 1:10 | 1:1000 | * |
| Volume | 2.0 | 1.0 | 4.0 |
| PPY dilution | 1:10 | 1:1000 | * |
| Volume | 2.0 | 1.0 | 4.0 |
|  | | | |
| 2554 | | | |
| Large | 1.54266 | 681.0751 | 0.048023 |
| Small | 3.172142 | 1315.977 | 0.096651 |
| Y | 3.613301 | 1413.083 | 0.087124 |
| Degradation index | 2.056281 | 1.932206 | 2.012616 |
| FUSION 6C dilution | 1:10 | 1:1000 | * |
| Volume | 2.0 | 0.5 | 5.0 |
| PPY dilution | 1:10 | 1:1000 | * |
| Volume | 2.0 | 0.5 | 5.0 |
|  | | | |
| 2556 | | | |
| Large | 16.24858 | 397.1853 | 0.01494 |
| Small | 27.18975 | 831.0268 | 0.043496 |
| Y | 30.78499 | 765.8901 | 0.041953 |
| Degradation index | 1.673361 | 2.09229 | 2.911406 |
| FUSION 6C dilution | 1:40 | 1:1000 | * |
| Volume | 1.0 | 0.5 | 10.0 |
| PPY dilution | 1:40 | 1:1000 | * |
| Volume | 1.0 | 0.5 | 10.0 |
|  | | | |
| 2557 | | | |
| Large | 3.170326 | 211.5256 | 0.000223 |
| Small | 8.107934 | 644.395 | 0.022051 |
| Y | 8.402453 | 341.4392 | 0.010706 |
| Degradation index | 2.557445 | 1.628148 | 99.07501 |
| FUSION 6C dilution | 1:20 | 1:500 | * |
| Volume | 1.0 | 1.0 | 15.0 |
| PPY dilution | 1:20 | 1:500 | * |
| Volume | 1.0 | 1.0 | 15.0 |
|  | | | |
| 2558 | | | |
| Large | 13.72523 | 374.3087 | 0.52741 |
| Small | 19.34358 | 658.7626 | 1.120262 |
| Y | 23.25303 | 722.8544 | 1.116512 |
| Degradation index | 1.409345 | 1.759945 | 2.124084 |
| FUSION 6C dilution | 1:30 | 1:1000 | * |
| Volume | 1.0 | 1.0 | 0.5 |
| PPY dilution | 1:30 | 1:1000 | * |
| Volume | 1.0 | 1.0 | 0.5 |
|  | | | |

| 2561 | | | |
| --- | --- | --- | --- |
| Large | 1.458411 | 444.722 | 0.1126 |
| Small | 2.165123 | 876.1624 | 0.535133 |
| Y | 2.526806 | 881.7307 | 0.56985 |
| Degradation index | 1.484577 | 1.970135 | 4.75252 |
| FUSION 6C dilution | 1:10 | 1:1000 | * |
| Volume | 2.0 | 1.0 | 1.0 |
| PPY dilution | 1:10 | 1:1000 | * |
| Volume | 2.0 | 1.0 | 1.0 |
|  | | | |
| 2562 | | | |
| Large | 1.770389 | 404.0191 | 0.039697 |
| Small | 3.118907 | 704.8669 | 0.10213 |
| Y | 3.176708 | 690.7513 | 0.093994 |
| Degradation index | 1.761708 | 1.744638 | 2.572754 |
| FUSION 6C dilution | 1:10 | 1:1000 | * |
| Volume | 2.0 | 0.5 | 5.0 |
| PPY dilution | 1:10 | 1:1000 | * |
| Volume | 2.0 | 0.5 | 5.0 |
|  | | | |
| 2563 | | | |
| Large | 10.88239 | 215.6621 | 0.009575 |
| Small | 20.65639 | 315.2034 | 0.024621 |
| Y | 13.79356 | 218.8234 | 0.018596 |
| Degradation index | 1.898149 | 1.461561 | 2.571328 |
| FUSION 6C dilution | 1:30 | 1:500 | * |
| Volume | 1.0 | 1.0 | 15.0 |
| PPY dilution | 1:30 | 1:500 | * |
| Volume | 0.5 | 1.0 | 15.0 |
|  | | | |
| 2564 | | | |
| Large | 3.210573 | 396.9126 | 0.033746 |
| Small | 5.042576 | 687.3063 | 0.063555 |
| Y | 4.86042 | 680.3604 | 0.063975 |
| Degradation index | 1.570615 | 1.731631 | 1.88331 |
| FUSION 6C dilution | 1:10 | 1:1000 | * |
| Volume | 1.0 | 1.0 | 10.0 |
| PPY dilution | 1:10 | 1:1000 | * |
| Volume | 1.0 | 1.0 | 10.0 |
|  | | | |
| 2565 | | | |
| Large | 5.868193 | 174.6342 | 0.011265 |
| Small | 15.32791 | 256.9063 | 0.029391 |
| Y | 25.33725 | 328.4339 | 0.034652 |
| Degradation index | 2.612032 | 1.47111 | 2.608959 |
| FUSION 6C dilution | 1:20 | 1:500 | * |
| Volume | 1.0 | 1.0 | 15.0 |
| PPY dilution | 1:20 | 1:500 | * |
| Volume | 0.5 | 1.0 | 15.0 |
|  | | | |
| 2567 | | | |
| Large | 0.789071 | 162.6572 | 0.011353 |
| Small | 0.978289 | 143.7167 | 0.075466 |
| Y | 1.13157 | 218.6766 | 0.035707 |
| Degradation index | 1.239798 | 0.883556 | 6.647031 |
| FUSION 6C dilution | * | 1:500 | * |
| Volume | 0.5 | 2.0 | 10.0 |
| PPY dilution | * | 1:500 | * |
| Volume | 0.5 | 2.0 | 15.0 |
|  | | | |
| 2568 | | | |
| Large | 0.509721 | 148.1834 | 0.026934 |
| Small | 0.821744 | 218.2094 | 0.128852 |
| Y | 0.923459 | 251.9923 | 0.138337 |
| Degradation index | 1.612144 | 1.472562 | 4.783975 |
| FUSION 6C dilution | * | 1:500 | * |
| Volume | 0.5 | 1.0 | 5.0 |
| PPY dilution | * | 1:500 | * |
| Volume | 0.5 | 1.0 | 5.0 |
|  | | | |
| 2603 | | | |
| Large | 3.040398 | 214.4446 | 0.009914 |

| Small | 6.862943 | 273.629 | 0.026563 |
| --- | --- | --- | --- |
| Y | 8.28522 | 356.5529 | 0.02566 |
| Degradation index | 2.257251 | 1.275989 | 2.679462 |
| FUSION 6C dilution | 1:10 | 1:500 | * |
| Volume | 1.0 | 1.0 | 15.0 |
| PPY dilution | 1:10 | 1:500 | * |
| Volume | 1.0 | 1.0 | 15.0 |
|  | | | |
| 2610 | | | |
| Large | 14.40844 | 321.446 | 0.117511 |
| Small | 29.28795 | 518.8289 | 0.255655 |
| Y | 41.92225 | 563.1808 | 0.205775 |
| Degradation index | 2.032695 | 1.614047 | 2.175583 |
| FUSION 6C dilution | 1:40 | 1:1000 | * |
| Volume | 1.0 | 1.0 | 2.0 |
| PPY dilution | 1:40 | 1:1000 | * |
| Volume | 0.5 | 1.0 | 3.0 |
|  | | | |
| 2612 | | | |
| Large | 0.364352 | 197.9781 | 0.346524 |
| Small | 1.03365 | 299.1764 | 1.438291 |
| Y | 1.230313 | 321.1305 | 1.706978 |
| Degradation index | 2.836956 | 1.51116 | 4.150621 |
| FUSION 6C dilution | * | 1:500 | * |
| Volume | 0.5 | 1.0 | 0.5 |
| PPY dilution | * | 1:500 | * |
| Volume | 0.5 | 1.0 | 0.5 |
|  | | | |
| 2613 | | | |
| Large | 2.060558 | 285.9974 | 0.009976 |
| Small | 3.459818 | 554.8238 | 0.033594 |
| Y | 3.0789 | 395.3463 | 0.025974 |
| Degradation index | 1.679069 | 1.940311 | 3.367586 |
| FUSION 6C dilution | 1:10 | 1:1000 | * |
| Volume | 2.0 | 1.0 | 15.0 |
| PPY dilution | 1:10 | 1:1000 | * |
| Volume | 2.0 | 1.0 | 15.0 |
|  | | | |
| 2614 | | | |
| Large | 2.651711 | 210.2489 | 0.032568 |
| Small | 4.996408 | 441.4192 | 0.099194 |
| Y | 6.429025 | 443.8855 | 0.088736 |
| Degradation index | 1.884221 | 2.099507 | 30045734 |
| FUSION 6C dilution | 1:10 | 1:1000 | * |
| Volume | 1.0 | 1.0 | 5.0 |
| PPY dilution | 1:10 | 1:1000 | * |
| Volume | 1.0 | 1.0 | 5.0 |
|  | | | |
| 2615 | | | |
| Large | 1.425065 | 261.1766 | 0.023589 |
| Small | 2.259261 | 440.4669 | 0.094537 |
| Y | 2.742595 | 485.9885 | 0.85836 |
| Degradation index | 1.585374 | 1.686472 | 4.007673 |
| FUSION 6C dilution | 1:10 | 1:1000 | * |
| Volume | 2.0 | 1.0 | 5.0 |
| PPY dilution | 1:10 | 1:1000 | * |
| Volume | 2.0 | 1.0 | 5.0 |
|  | | | |
| 2616 | | | |
| Large | 3.952221 | 268.0953 | 0.952911 |
| Small | 7.693162 | 414.7448 | 3.219313 |
| Y | 10.77692 | 442.2723 | 2.568954 |
| Degradation index | 1.946541 | 1.547005 | 3.378397 |
| FUSION 6C dilution | 1:10 | 1:1000 | 1:10 |
| Volume | 1.0 | 1.0 | 2.0 |
| PPY dilution | 1:10 | 1:1000 | 1:10 |
| Volume | 0.5 | 1.0 | 2.0 |
|  | | | |
| 2618 | | | |
| Large | 1.756011 | 148.911 | 0.075507 |
| Small | 2.33252 | 161.266 | 0.210499 |
| Y | 3.194579 | 229.7551 | 0.2125 |

| Degradation index | 1.328249 | 1.082968 | 2.787804 |
| --- | --- | --- | --- |
| FUSION 6C dilution | 1:10 | 1:500 | * |
| Volume | 2.0 | 3.0 | 3.0 |
| PPY dilution | 1:10 | 1:500 | * |
| Volume | 2.0 | 3.0 | 3.0 |
|  | | | |
| 2619 | | | |
| Large | 5.159396 | 244.5792 | 0.000743 |
| Small | 12.01876 | 429.5208 | 0.004846 |
| Y | 15.12896 | 311.2011 | 0.002745 |
| Degradation index | 2.329489 | 1.756162 | 6.525216 |
| FUSION 6C dilution | 1:20 | 1:1000 | * |
| Volume | 1.0 | 1.0 | 15.0 |
| PPY dilution | 1:20 | 1:1000 | * |
| Volume | 1.0 | 1.0 | 15.0 |
|  | | | |
| 2620 | | | |
| Large | 4.909695 | 212.8342 | 0.029387 |
| Small | 9.541229 | 324.554 | 0.080354 |
| Y | 14.22695 | 372.5565 | 0.086412 |
| Degradation index | 1.943345 | 1.510819 | 2.734338 |
| FUSION 6C dilution | 1:20 | 1:500 | * |
| Volume | 1.0 | 1.0 | 5.0 |
| PPY dilution | 1:20 | 1:500 | * |
| Volume | 0.5 | 1.0 | 5.0 |
|  | | | |
| 2622 | | | |
| Large | 4.864139 | 97.91629 | 0.310292 |
| Small | 10.33898 | 138.2761 | 0.838602 |
| Y | 13.16821 | 150.4968 | 0.81639 |
| Degradation index | 2.125552 | 1.412187 | 2.702621 |
| FUSION 6C dilution | 1:20 | 1:500 | * |
| Volume | 1.0 | 3.0 | 0.5 |
| PPY dilution | 1:20 | 1:500 | * |
| Volume | 1.0 | 3.0 | 0.5 |
|  | | | |
| 2624 | | | |
| Large | 3.033331 | 293.3608 | 0.005686 |
| Small | 7.753662 | 617.1726 | 0.02408 |
| Y | 7.243911 | 466.0439 | 0.018969 |
| Degradation index | 2.556154 | 2.103801 | 4.234628 |
| FUSION 6C dilution | 1:10 | 1:1000 | * |
| Volume | 1.0 | 1.0 | 15.0 |
| PPY dilution | 1:10 | 1:1000 | * |
| Volume | 1.0 | 1.0 | 15.0 |
|  | | | |
| 2626 | | | |
| Large | 0.666733 | 357.9069 | 0.023904 |
| Small | 1.550852 | 561.7985 | 0.064971 |
| Y | 2.258561 | 591.3342 | 0.063315 |
| Degradation index | 2.326046 | 1.569678 | 2.7117939 |
| FUSION 6C dilution | * | 1:1000 | * |
| Volume | 0.5 | 1.0 | 10.0 |
| PPY dilution | * | 1:1000 | * |
| Volume | 0.5 | 1.0 | 10.0 |
|  | | | |
| 2627 | | | |
| Large | 0.237652 | 81.62191 | 0.016088 |
| Small | 1.003621 | 103.1445 | 0.057649 |
| Y | 0.751577 | 91.1542 | 0.060384 |
| Degradation index | 4.2207 | 1.263687 | 3.583356 |
| FUSION 6C dilution | * | 1:200 | * |
| Volume | 0.5 | 1.0 | 10.0 |
| PPY dilution | * | 1:200 | * |
| Volume | 1.0 | 1.0 | 10.0 |
|  | | | |
| 2629 | | | |
| Large | 0.102381 | 432.6681 | 0.015922 |
| Small | 0.262039 | 695.3394 | 0.07575 |
| Y | 0.29223 | 717.9774 | 0.08696 |
| Degradation index | 2.559433 | 1.607097 | 4.757416 |
| FUSION 6C dilution | * | 1:1000 | * |
| Volume | 2.0 | 1.0 | 10.0 |
| PPY dilution | * | 1:1000 | * |
| Volume | 2.0 | 1.0 | 10.0 |
|  | | | |

| 2630 | | | |
| --- | --- | --- | --- |
| Large | 3.654274 | 437.4425 | 0.286535 |
| Small | 7.185802 | 471.8078 | 0.382044 |
| Y | 8.853642 | 505.9128 | 0.399303 |
| Degradation index | 1.966411 | 1.07856 | 1.333322 |
| FUSION 6C dilution | 1:10 | 1:1000 | * |
| Volume | 1.0 | 1.0 | 2.0 |
| PPY dilution | 1:10 | 1:1000 | * |
| Volume | 1.0 | 1.0 | 2.0 |
|  | | | |
| 2655 | | | |
| Large | 1.080196 | 207.7086 | 0.016229 |
| Small | 1.316036 | 174.3559 | 0.026201 |
| Y | 2.078187 | 276.4256 | 0.034554 |
| Degradation index | 1.218331 | 0.839425 | 1.61453 |
| FUSION 6C dilution | * | 1:200 | * |
| Volume | 0.5 | 1.0 | 15.0 |
| PPY dilution | * | 1:200 | * |
| Volume | 0.5 | 1.0 | 15.0 |
|  | | | |
| 2657 | | | |
| Large | 0.447914 | 331.874 | 0.004299 |
| Small | 1.379497 | 325.77 | 0.008394 |
| Y | 1.272416 | 402.0777 | 0.008439 |
| Degradation index | 3.079825 | 0.981607 | 1.952517 |
| FUSION 6C dilution | * | 1:500 | * |
| Volume | 0.5 | 1.0 | 15.0 |
| PPY dilution | * | 1:500 | * |
| Volume | 0.5 | 1.0 | 15.0 |
