# Supplementary material for: Systematic STR analysis of old post-vasectomy seminal fluid stains to examine evidence stored for 16 years
Source: Sci Rep. 2021 Apr 26;11:8918. doi: 10.1038/s41598-021-87937-x (PMC8076208; doi:10.1038/s41598-021-87937-x)
Supplement: Supplementary file 4 — Supplementary Information 4. [file 41598_2021_87937_MOESM4_ESM.docx]

**Supplementary information**

**Systematic STR analysis of old post-vasectomy seminal fluid stains to examine evidence stored for 16 years**

Julianna Kesselring Romero^1^, Eloisa Auler Bittencourt^1,2^, José Arnaldo Soares-Vieira^3^, Ana Claudia Pacheco^4^, Alexandre Learth Soares^4^, Edna Sadayo Miazato Iwamura ^1^*

^1^ Laboratório de Patologia Moslecular, Departamento de Patologia - Escola Paulista de Medicina /Universidade Federal de São Paulo (EPM/UNIFESP), SP, Brazil; ^2^ Academia de Polícia de São Paulo (ACADEPOL), SP, Brazil; ^3^ Departamento de Medicina Legal, Ética Médica, Medicina Social e do Trabalho- Faculdade de Medicina da Universidade São Paulo (USP), SP, Brazil; ^4^ Instituto de Criminalística-Superintendência da Polícia Técnico-Científica do Estado de São Paulo (SPTC SP), SP, Brazil

* Corresponding author : Edna Sadayo Miazato Iwamura, phone +55 11 5576 4848 ext 1386, e-mail: edna.iwamura@unifesp.com

Departamento de Patologia, Escola Paulista de Medicina/Universidade Federal de São Paulo (EPM/UNIFESP), Rua Botucatu 740, Edifício Lemos Torres. Vila Clementino- CEP 04023-62, São Paulo, SP- Brazil.

**Supplementary table 2 -** Genetic Profile of PowerPlex Fusion 6C and PowerPlex 23Y from blood DNA (2n), semen stains pre vasectomy DNA (n) and ejaculate stains post vasectomy. The volume used for amplifications was according to supplementary S2.

Sample 2364

PowerPlex Fusion 6C

| **DNA *Locus*** | **2364**  **Blood** | **2364**  **Pre vasectomy semen** | **2364**  **Post vasectomy seminal fluid** |
| --- | --- | --- | --- |
| **D3S1358** | 14/16 | 14/16 | 14/16 |
| **D1S1656** | 16/18.3 | 16/18.3 | * |
| **D2S441** | 14 | 14 | * |
| **D10S1248** | 16 | 16 | * |
| **D13S317** | 12/14 | 12/14 | * |
| **Penta E** | 9 | 9 | * |
| **D16S539** | 12/13 | 12/13 | 12/13 |
| **D18S51** | 12/16 | 12/16 | 12/16 |
| **D2S1338** | 16/18 | 16/18 | * |
| **CSF1PO** | 8/12 | 8/12 | * |
| **Penta D** | 10/14 | 14 | * |
| **TH01** | 9.3 | 9.3 | 9.3 |
| **vWA** | 17/18 | 17/18 | 17/18 |
| **D21S11** | 29 | 29 | * |
| **D7S820** | 8/9 | 8/9 | * |
| **D5S818** | 12 | 12 | * |
| **TPOX** | 11 | * |  |
| **D8S1179** | 10/13 | 10/13 | 10/13 |
| **D12S391** | 17/22 | 17/22 | 17 |
| **D19S433** | 11/14 | 11/14 | 14 |
| **SE33** | 17/21 | 17/21 | 17 |
| **D22S1045** | 11/15 | * | * |
| **DYS391** | 11 | 11 | 11 |
| **FGA** | 24/25 | 24/25 | 24/25 |
| **DYS576** | 18 | 18 | * |
| **DYS570** | 20 | 20 | * |
| **Amelogenina** | XY  (male) | XY  (male) | XY  (male) |

Sample 2364

PowerPlex Y23 System

| **Y STR *locus*** | **2364**  **Blood** | **2364**  **Pre vasectomy semen** | **2364**  **Post vasectomy seminal fluid** |
| --- | --- | --- | --- |
| **DYS576** | 18 |  |  |
| **DYS389I** | 14 |  | 14 |
| **DYS448** | 20 |  |  |
| **DYS389II** | 31 |  |  |
| **DYS19** | 16 |  |  |
| **DYS391** | 11 |  | 11 |
| **DYS481** | 25 |  |  |
| **DYS549** | 11 |  |  |
| **DYS533** | 12 |  |  |
| **DYS438** | 10 |  |  |
| **DYS437** | 14 |  |  |
| **DYS570** | 20 |  | 20 |
| **DYS635** | 20 |  |  |
| **DYS390** | 24 |  |  |
| **DYS439** | 11 |  |  |
| **DYS392** | 11 |  |  |
| **DYS643** | 12 |  |  |
| **DYS393** | 13 |  | 13 |
| **DYS458** | 17 |  | 17 |
| **DYS385** | 12/17 |  |  |
| **DYS456** | 17 |  |  |
| **Y_GATA_H4** | 11 |  |  |

sample 2365

PowerPlex Fusion 6C

| **DNA *locus*** | **2365**  **Blood** | **2365**  **Pre vasectomy semen** | **2365**  **Post vasectomy seminal fluid** |
| --- | --- | --- | --- |
| **D3S1358** | 15/19 | 15/19 | 15/19 |
| **D1S1656** | 17/17.3 | 17/17.3 | 17/17.3 |
| **D2S441** | 10/11 | 10/11 | 10/11 |
| **D10S1248** | 14 | 14 | 14 |
| **D13S317** | 12 | 12 | 12 |
| **Penta E** | 5 | 5 | 5 |
| **D16S539** | 9/13 | 9/13 | 9/13 |
| **D18S51** | 15/19 | 15/19 | 15/19 |
| **D2S1338** | 19/24 | 19/24 | 19/24 |
| **CSF1PO** | 10/12 | 10/12 | 10/12 |
| **Penta D** | 9/11 | 9/11 | 9/11 |
| **TH01** | 8/10 | 8/10 | 8/10 |
| **vWA** | 14/16 | 14/16 | 14/16 |
| **D21S11** | 28/33.2 | 28/33.2 | 28/33.2 |
| **D7S820** | 10/11 | 10/11 | 10/11 |
| **D5S818** | 11 | 11 | 11 |
| **TPOX** | 8/9 | 8/9 | 9 |
| **D8S1179** | 12/16 | 12/16 | 12/16 |
| **D12S391** | 15/16 | 15/16 | 15/16 |
| **D19S433** | 14/15 | 14/15 | 14/15 |
| **SE33** | 19 | 19 | 19 |
| **D22S1045** | 16 | 16 | 16 |
| **DYS391** | 10 | 10 | 10 |
| **FGA** | 23/24 | 23/24 | 23/24 |
| **DYS576** | 18 | 18 | 18 |
| **DYS570** | 17 | 17 | 17 |
| **Amelogenina** | XY  (male) | XY  (male) | XY  (male) |

PowerPlex Y23 System

| ***DNA locus* Y STR** | **2365**  **Blood** | **2365**  **Pre vasectomy semen** | **2365**  **Post vasectomy seminal fluid** |
| --- | --- | --- | --- |
| **DYS576** | 18 | 18 | 18 |
| **DYS389I** | 13 | 13 | 13 |
| **DYS448** | 23 | 23 | 23 |
| **DYS389II** | 31 | 31 | 31 |
| **DYS19** | 15 | 15 | 15 |
| **DYS391** | 10 | 10 | 10 |
| **DYS481** | 25 | 25 | 25 |
| **DYS549** | 12 | 12 | 12 |
| **DYS533** | 11 | 11 | 11 |
| **DYS438** | 10 | 10 | 10 |
| **DYS437** | 14 | 14 | 14 |
| **DYS570** | 17 | 17 | 17 |
| **DYS635** | 17 | 17 | 17 |
| **DYS390** | 24 | 24 | 24 |
| **DYS439** | 14 | 14 | 14 |
| **DYS392** | 11 | 11 | 11 |
| **DYS643** | 13 | 13 | 13 |
| **DYS393** | 13 | 13 | 13 |
| **DYS458** | 18 | 18 | 18 |
| **DYS385** | 11 | 11 | 11 |
| **DYS456** | 13 | 13 | 13 |
| **Y_GATA_H4** | 12 | 12 | 12 |

sample 2367

PowerPlex Fusion 6C

| ***DNA locus*** | **2367**  **Blood** | **2367**  **Pre vasectomy semen** | **2367**  **Post vasectomy seminal fluid** |
| --- | --- | --- | --- |
| **D3S1358** | 15/18 | 15/18 | 15/18 |
| **D1S1656** | 12/14 | 12/14 | 12/14 |
| **D2S441** | 10/14 | 10/14 | 10/14 |
| **D10S1248** | 13 | 13 | 13 |
| **D13S317** | 11/12 | 11/12 | 11/12 |
| **Penta E** | 8/17 | 8/17 | 8/17 |
| **D16S539** | 11 | 11 | 11 |
| **D18S51** | 18/20 | 18/20 | 18/20 |
| **D2S1338** | 17 | 17 | 17 |
| **CSF1PO** | 10/11 | 10/11 | 10/11 |
| **Penta D** | 10/11 | 10/11 | 11 |
| **TH01** | 7 | 7 | 7 |
| **vWA** | 17 | 17 | 17 |
| **D21S11** | 28/31.2 | 28/31.2 | 28/31.2 |
| **D7S820** | 9/10 | 9/10 | 9/10 |
| **D5S818** | 11/12 | 11/12 | 11/12 |
| **TPOX** | 8 | 8 | 8 |
| **D8S1179** | 12/14 | 12/14 | 12/14 |
| **D12S391** | 18/19 | 18/19 | 18/19 |
| **D19S433** | 14/15.2 | 14/15.2 | 14/15.2 |
| **SE33** | 20/30.2 | 20/30.2 | 20/30.2 |
| **D22S1045** | 11 | 11 | 11 |
| **DYS391** | 11 | 11 | 11 |
| **FGA** | 21/24 | 21/24 | 21/24 |
| **DYS576** | 20 | 20 | 20 |
| **DYS570** | 17 | 17 | * |
| **Amelogenina** | XY  (male) | XY  (male) | XY  (male) |

PowerPlex Y23 System

| ***DNA locus* Y STR** | **2367**  **Blood** | **2367**  **Pre vasectomy semen** | **2367**  **Post vasectomy seminal fluid** |
| --- | --- | --- | --- |
| **DYS576** | 20 | 20 | 20 |
| **DYS389I** | 14 | 14 | 14 |
| **DYS448** | 18 | 18 | 18 |
| **DYS389II** | 32 | 32 | 32 |
| **DYS19** | 15 | 15 | 15 |
| **DYS391** | 11 | 11 | 11 |
| **DYS481** | 22 | 22 | 22 |
| **DYS549** | 12 | 12 | 12 |
| **DYS533** | 11 | 11 | 11 |
| **DYS438** | 12 | 12 | 12 |
| **DYS437** | 14 | 14 |  |
| **DYS570** | 17 | 17 | 17 |
| **DYS635** | 24 | 24 | 24 |
| **DYS390** | 24 | 24 | 24 |
| **DYS439** | 13 | 13 | 13 |
| **DYS392** | 13 | 13 | 13 |
| **DYS643** | 10 | 10 | 10 |
| **DYS393** | 13 | 13 | 13 |
| **DYS458** | 18 | 18 | 18 |
| **DYS385** | 11/13 | 11/13 | 11/13 |
| **DYS456** | 16 | 16 | 16 |
| **Y_GATA_H4** | 12 | 12 | 12 |

sample 2368

PowerPlex Fusion 6C

| ***DNA locus*** | 2368  **Blood** | **2368**  **Pre vasectomy semen** | **2368**  **Post vasectomy seminal fluid** |
| --- | --- | --- | --- |
| **D3S1358** | 15/16 | 15/16 | 15/16 |
| **D1S1656** | 12/17.3 | 12/17.3 | 12/17.3 |
| **D2S441** | 10 | 10 | 10 |
| **D10S1248** | 14/16 | 14/16 | 14/16 |
| **D13S317** | 13/14 | 13/14 | 13 (9/11) |
| **Penta E** | 7/14 | * | 7 |
| **D16S539** | 9 | 9 | 9 |
| **D18S51** | 14/17 | 14/17 | 14/17 |
| **D2S1338** | 17/19 | 17/19 | 17/19 |
| **CSF1PO** | 7/11 | 7/11 | 7/10 |
| **Penta D** | 10/11 | 10/11 | 10/11 |
| **TH01** | 7/9 | 7/9 | 7/9 |
| **vWA** | 17 | 17 | 17 |
| **D21S11** | 29/35 | 29/35 | 29/35 |
| **D7S820** | 10/11 | 10/11 | 10/11 |
| **D5S818** | 10/13 | 13 | 12 |
| **TPOX** | 11 | 11 | 9/11 |
| **D8S1179** | 14/17 | 14/17 | 14/17 |
| **D12S391** | 15/20 | 15/20 | 15/20 |
| **D19S433** | 14/15 | 14/15 | 14/15 |
| **SE33** | 16/18 | 16/18 | 16/18 |
| **D22S1045** | 14/15 | * | 15 |
| **DYS391** | 9 | 9 | 9 |
| **FGA** | 21/27 | 21/27 | 21/27 |
| **DYS576** | 16 | 16 | 17 |
| **DYS570** | 18 | 18 | 16 |
| **Amelogenina** | XY  (male) | XY  (male) | XY  (male) |

PowerPlex Y23 System

| ***DNA locus* Y STR** | 2368  **Blood** | **2368**  **Pre vasectomy semen** | **2368**  **Post vasectomy seminal fluid** |
| --- | --- | --- | --- |
| **DYS576** | 16 | 16 | 16 |
| **DYS389I** | 13 | 13 | 13 |
| **DYS448** | 21 | 21 | 21 |
| **DYS389II** | 29 | 29 | 29 |
| **DYS19** | 15 | 15 |  |
| **DYS391** | 9 | 9 | 9 |
| **DYS481** | 22 | 22 | 22 |
| **DYS549** | 12 | 12 | 12 |
| **DYS533** | 12 | 12 | 12 |
| **DYS438** | 9 | 9 | 9 |
| **DYS437** | 14 | 14 |  |
| **DYS570** | 18 | 18 | 18 |
| **DYS635** | 23 | 23 | 23 |
| **DYS390** | 23 | 23 | 23 |
| **DYS439** | 13 | 12 | 13 |
| **DYS392** | 11 | 11 |  |
| **DYS643** | 10 | 10 |  |
| **DYS393** | 12 | 12 | 12 |
| **DYS458** | 14 | 14 | 14 |
| **DYS385** | 13/16 | 13/16 | 13/16 |
| **DYS456** | 16 | 16 |  |
| **Y_GATA_H4** | 12 | 12 |  |

sample 2369

PowerPlex Fusion 6C

| ***DNA locus*** | **2369**  **Blood** | **2369**  **Pre vasectomy semen** | **2369**  **Post vasectomy seminal fluid** |
| --- | --- | --- | --- |
| **D3S1358** | 16 | 16 | 16 |
| **D1S1656** | 17.3/18.3 | 17.3/18.3 | 17.3/18.3 |
| **D2S441** | 10/13 | 10/13 | 10/13 |
| **D10S1248** | 14/15 | 14/15 | 14/15 |
| **D13S317** | 8/12 | 8/12 | 8/12 |
| **Penta E** | 11 | 11 | 11 |
| **D16S539** | 11/13 | 11/13 | 11/13 |
| **D18S51** | 17 | 17 | 17 |
| **D2S1338** | 17/19 | 17/19 | 17/19 |
| **CSF1PO** | 12/14 | 12/14 | 12 |
| **Penta D** | 10/13 | 10/13 | 10/13 |
| **TH01** | 7 | 7 | 7 |
| **vWA** | 16/17 | 16/17 | 16/17 |
| **D21S11** | 28/30 | 28/30 | 28/30 |
| **D7S820** | 12/13 | 12/13 | 12/13 |
| **D5S818** | 12 | 12 | 12 |
| **TPOX** | 8 | 8 | * |
| **D8S1179** | 11/13 | 11/13 | 11/13 |
| **D12S391** | 20/23 | 20/23 | 20/23 |
| **D19S433** | 13/15 | 13/15 | 13/15 |
| **SE33** | 17/18 | 17/18 | 17/18 |
| **D22S1045** | 15/16 | 15/16 | 15 |
| **DYS391** | 10 | 10 | 10 |
| **FGA** | 21/25 | 21/25 | 21/25 |
| **DYS576** | 18 | 18 | 18 |
| **DYS570** | 18 | 18 | * |
| **Amelogenina** | XY  (male) | XY  (male) | XY  (male) |

PowerPlex Y23 System

| ***DNA locus* Y STR** | **2369**  **Blood** | **2369**  **Pre vasectomy semen** | **2369**  **Post vasectomy seminal fluid** |
| --- | --- | --- | --- |
| **DYS576** | 18 | 18 | 18 |
| **DYS389I** | 13 | 13 | 13 |
| **DYS448** | 19 | 19 | 19 |
| **DYS389II** | 30 | 30 |  |
| **DYS19** | 13 | 13 | 13 |
| **DYS391** | 10 | 10 | 10 |
| **DYS481** | 24 | 24 | 24 |
| **DYS549** | 12 | 12 | 12 |
| **DYS533** | 11 | 11 | 11 |
| **DYS438** | 10 | 10 | 10 |
| **DYS437** | 14 | 14 |  |
| **DYS570** | 18 | 18 | 18 |
| **DYS635** | 23 | 23 | 23 |
| **DYS390** | 24 | 24 | 24 |
| **DYS439** | 13 | 13 | 13 |
| **DYS392** | 11 | 11 |  |
| **DYS643** | 12 | 12 |  |
| **DYS393** | 12 | 12 | 12 |
| **DYS458** | 17 | 17 | 17 |
| **DYS385** | 16 | 16 | 16 |
| **DYS456** | 15 | 15 | 15 |
| **Y_GATA_H4** | 11 | 11 | 11 |

sample 2405

PowerPlex Fusion 6C

| ***DNA locus*** | **2405**  **Blood** | **2405**  **Pre vasectomy semen** | **2405**  **Post vasectomy seminal fluid** |
| --- | --- | --- | --- |
| **D3S1358** | 14/16 | 14/16 | 14/16 |
| **D1S1656** | 11/15 | 11/15 | 11/15 |
| **D2S441** | 11 | 11 | 11 |
| **D10S1248** | 14/15 | 14/15 | 14/15 |
| **D13S317** | 11/12 | 11/12 | * |
| **Penta E** | 7/13 | 7/13 | * |
| **D16S539** | 11/13 | 11/13 | 11/13 |
| **D18S51** | 13/17 | 13/17 | 13/17 |
| **D2S1338** | 26/28 | 26/28 | 26/28 |
| **CSF1PO** | 10/11 | 10/11 | 10/11 |
| **Penta D** | 9/13 | 9/13 | * |
| **TH01** | 7/9.3 | 7/9.3 | 7/9.3 |
| **vWA** | 15/16 | 15/16 | 15/16 |
| **D21S11** | 30.2/32.2 | 30.2/32.2 | 30.2/32.2 |
| **D7S820** | 11/13 | 11/13 | 11 |
| **D5S818** | 12 | 12 | 12 |
| **TPOX** | 8/11 | 11 | * |
| **D8S1179** | 12/16 | 12/16 | 12/16 |
| **D12S391** | 20/20.3 | 20/20.3 | 20/20.3 |
| **D19S433** | 13/14 | 13/14 | 13/14 |
| **SE33** | 17/24.2 | 17/24.2 | 17/24.2 |
| **D22S1045** | 16/17 | 16 | * |
| **DYS391** | 11 | 11 | 11 |
| **FGA** | 18/23 | 18/23 | 18/23 |
| **DYS576** | 17 | 17 | * |
| **DYS570** | 16 | 16 | * |
| **Amelogenina** | XY  (male) | XY  (male) | XY  (male) |

PowerPlex Y23 System

| ***DNA locus* Y STR** | **2405**  **Blood** | **2405**  **Pre vasectomy semen** | **2405**  **Post vasectomy seminal fluid** |
| --- | --- | --- | --- |
| **DYS576** | 17 | 17 | 17 |
| **DYS389I** | 13 | 13 | 13 |
| **DYS448** | 19 | 19 | 19 |
| **DYS389II** | 29 | 29 | 29 |
| **DYS19** | 14 | 14 |  |
| **DYS391** | 11 | 11 | 11 |
| **DYS481** | 23 | 23 | 23 |
| **DYS549** | 12 | 12 | 12 |
| **DYS533** | 12 | 12 | 12 |
| **DYS438** | 11 | 11 | 11 |
| **DYS437** | 15 | 15 |  |
| **DYS570** | 16 | 16 | 16 |
| **DYS635** | 23 | 23 | 23 |
| **DYS390** | 24 | 24 | 24 |
| **DYS439** | 13 | 13 |  |
| **DYS392** | 13 | 13 |  |
| **DYS643** | 10 | 10 |  |
| **DYS393** | 13 | 13 | 13 |
| **DYS458** | 17 | 17 | 17 |
| **DYS385** | 11/15 | 11/15 |  |
| **DYS456** | 16 | 16 | 16 |
| **Y_GATA_H4** | 12 | 12 |  |

sample 2406

PowerPlex Fusion 6C

| ***DNA locus*** | **2406**  **Blood** | **2406**  **Pre vasectomy semen** | **2406**  **Post vasectomy seminal fluid** |
| --- | --- | --- | --- |
| **D3S1358** | 15/18 | 15/18 | 15/18 |
| **D1S1656** | 14/17.3 | 14/17.3 | 14/17.3 |
| **D2S441** | 11/14 | 11/14 | 11/14 |
| **D10S1248** | 15 | 15 | 15 |
| **D13S317** | 12/13 | 12/13 | 12/13 |
| **Penta E** | 8/13 | 8/13 | * |
| **D16S539** | 11/13 | 11/13 | 11/13 |
| **D18S51** | 15/16 | 15/16 | 15/16 |
| **D2S1338** | 20/23 | 20/23 | 20/23 |
| **CSF1PO** | 10/12 | 10/12 | * |
| **Penta D** | 11 | 11 | * |
| **TH01** | 6/7 | 6/7 | 6/7 |
| **vWA** | 15/17 | 15/17 | 15/17 |
| **D21S11** | 29/33.2 | 29/33.2 | 29/33.2 |
| **D7S820** | 10/11 | 10/11 | 11 |
| **D5S818** | 11 | 11 | * |
| **TPOX** | 6/11 | 6/11 | * |
| **D8S1179** | 13/14 | 13/14 | 13/14 |
| **D12S391** | 19/24 | 19/24 | 19/24 |
| **D19S433** | 13.2/15 | 13.2/15 | 13.2/15 |
| **SE33** | 26.2/30.2 | 26.2/30.2 | 30.2 |
| **D22S1045** | 16/17 | 16/17 | * |
| **DYS391** | 11 | 11 | 11 |
| **FGA** | 20 | 20 | 20/46.2 |
| **DYS576** | 14 | 14 | * |
| **DYS570** | 21 | 21 | * |
| **Amelogenina** | XY  (male) | XY  (male) | XY  (male) |

PowerPlex Y23 System

| ***DNA locus* Y STR** | **2406**  **Blood** | **2406**  **Pre vasectomy semen** | **2406**  **Post vasectomy seminal fluid** |
| --- | --- | --- | --- |
| **DYS576** | 14 | 14 | 14 |
| **DYS389I** | 13 | 13 | 13 |
| **DYS448** | 21 | 21 | 21 |
| **DYS389II** | 31 | 31 |  |
| **DYS19** | 15 | 15 |  |
| **DYS391** | 11 | 11 | 11 |
| **DYS481** | 28 | 28 | 28 |
| **DYS549** | 11 | 11 | 11 |
| **DYS533** | 12 | 12 | 12 |
| **DYS438** | 11 | 11 | 11 |
| **DYS437** | 14 | 14 |  |
| **DYS570** | 21 | 21 | 21 |
| **DYS635** | 21 | 21 | 21 |
| **DYS390** | 21 | 21 | 21 |
| **DYS439** | 12 | 12 |  |
| **DYS392** | 11 | 11 |  |
| **DYS643** |  | 15 |  |
| **DYS393** | 13 | 13 | 13 |
| **DYS458** | 18 | 18 | 18 |
| **DYS385** | 17/18 | 17/18 |  |
| **DYS456** | 15 | 15 | 15 |
| **Y_GATA_H4** |  | 12 |  |

sample 2407

PowerPlex Fusion 6C

| ***DNA locus*** | **2407**  **Blood** | **2407**  **Pre vasectomy semen** | **2407**  **Post vasectomy seminal fluid** |
| --- | --- | --- | --- |
| **D3S1358** | 16 | 15/17 | 15/17 |
| **D1S1656** | 14/15.3 | 13 | 13 |
| **D2S441** | 10/15 | 14 | 14 |
| **D10S1248** | 14 | 13/14 | 13/14 |
| **D13S317** | 8/12 | 8/9 | 8/9 |
| **Penta E** | 5/13 | 8/14 | * |
| **D16S539** | 9/12 | 11/12 | 11/12 |
| **D18S51** | 15/17 | 14/17 | 14/17 |
| **D2S1338** | 18/24 | 22/25 | 22/25 |
| **CSF1PO** | 10/12 | 10/12 | 12 |
| **Penta D** | 12/13 | 2.2/10 | 2.2 |
| **TH01** | 7/8 | 6/7 | 6/7 |
| **vWA** | 15/16 | 15/16 | 15/16 |
| **D21S11** | 27/31.2 | 29/32.2 | 29/32.2 |
| **D7S820** | 10/11 | 9 | 9 |
| **D5S818** | 9/11 | 12/13 | 13 |
| **TPOX** | 8/11 | 8 | * |
| **D8S1179** | 13/15 | 13/14 | 13/14 |
| **D12S391** | 17/22 | 18/25 | 18/25 |
| **D19S433** | 14/15 | 12.2/13 | 12.2/13 |
| **SE33** | 21.2/27.2 | 18/21 | 18/21 |
| **D22S1045** | 11/17 | 13/15 | * |
| **DYS391** | 10 | 10 | 10 |
| **FGA** | 19 | 19/24 | 19/24 |
| **DYS576** | 17 | 18 | * |
| **DYS570** | 17 | 18 | * |
| **Amelogenina** | XY  (male) | XY  (male) | XY  (male) |

PowerPlex Y23 System

| ***DNA locus* Y STR** | **2407**  **Blood** | **2407**  **Pre vasectomy semen** | **2407**  **Post vasectomy seminal fluid** |
| --- | --- | --- | --- |
| **DYS576** | 17 | 18 | 18 |
| **DYS389I** | 13 | 12 | 12 |
| **DYS448** | 19 | 20 | 20 |
| **DYS389II** | 29 | 29 |  |
| **DYS19** | 14 | 15 |  |
| **DYS391** | 10 | 10 | 10 |
| **DYS481** | 23 | 24 | 24 |
| **DYS549** | 13 | 11 | 11 |
| **DYS533** | 12 | 11 | 11 |
| **DYS438** | 11 | 9 |  |
| **DYS437** | 15 | 15 |  |
| **DYS570** | 17 | 18 | 18 |
| **DYS635** | 23 | 23 | 23 |
| **DYS390** | 23 | 23 | 23 |
| **DYS439** | 13 | 10 |  |
| **DYS392** | 12 | 11 |  |
| **DYS643** | 11 | 10 |  |
| **DYS393** | 13 | 12 | 12 |
| **DYS458** | 18 | 17 | 17 |
| **DYS385** | 11/12 | 13/16 |  |
| **DYS456** | 14 | 15 |  |
| **Y_GATA_H4** | 11 | 11 |  |

sample 2414

PowerPlex Fusion 6C

| ***DNA locus*** | **2414**  **Blood** | **2414**  **Pre vasectomy semen** | **2414**  **Post vasectomy seminal fluid** |
| --- | --- | --- | --- |
| **D3S1358** | 14/17 | 14/17 | 14/17 |
| **D1S1656** | 13/14 | 13/14 | 13/14 |
| **D2S441** | 10/14 | 10/14 | 10/14 |
| **D10S1248** | 12/14 | 12/14 | 14 |
| **D13S317** | 11/14 | 11/14 | * |
| **Penta E** | 8/15 | 8/15 | * |
| **D16S539** | 9/12 | 9/12 | 9/12 |
| **D18S51** | 15 ? OL | 15 | 15 |
| **D2S1338** | 15/27 | 15/27 | * |
| **CSF1PO** | 10/11 | 10/11 | 10 |
| **Penta D** | 9/10 | 9/10 | * |
| **TH01** | 6/9 | 6/9 | 6/9 |
| **vWA** | 16/17 | 16/17 | 16/17 |
| **D21S11** | 29/31.2 | 29/31.2 | 29/31.2 |
| **D7S820** | 10/11 | 10/11 | * |
| **D5S818** | 9/13 | 9/13 | * |
| **TPOX** | 9/11 | 9/11 | * |
| **D8S1179** | 13/14 | 13/14 | 13/14 |
| **D12S391** | 20.1 | 20 | 20 |
| **D19S433** | 13/16 | 13/16 | 13/16 |
| **SE33** | 18/20 | 18/20 | * |
| **D22S1045** | 15/16 | 15/16 | * |
| **DYS391** | 10 | 10 | 10 |
| **FGA** | 24/26 | 24/26 | 24/26 |
| **DYS576** | 20 | 20 | 20 |
| **DYS570** | 17 | 17 | 17 |
| **Amelogenina** | XY  (male) | XY  (male) | XY  (male) |

PowerPlex Y23 System

| ***DNA locus* Y STR** | **2414**  **Blood** | **2414**  **Pre vasectomy semen** | **2414**  **Post vasectomy seminal fluid** |
| --- | --- | --- | --- |
| **DYS576** | 20 | 20 |  |
| **DYS389I** | 13 | 13 | 13 |
| **DYS448** | 19 | 19 |  |
| **DYS389II** | 29 | 29 |  |
| **DYS19** | 14 | 14 |  |
| **DYS391** | 10 | 10 |  |
| **DYS481** | 22 | 22 | 22 |
| **DYS549** | 13 | 13 |  |
| **DYS533** | 13 | 13 |  |
| **DYS438** | 12 | 12 |  |
| **DYS437** | 15 | 15 |  |
| **DYS570** | 17 | 17 |  |
| **DYS635** | 25 | 25 | 25 |
| **DYS390** | 24 | 24 |  |
| **DYS439** | 12 | 12 |  |
| **DYS392** | 13 | 13 |  |
| **DYS643** | 10 | 10 |  |
| **DYS393** | 12 | 12 | 12 |
| **DYS458** | 17 | 17 | 17 |
| **DYS385** | 11/14 | 11/14 |  |
| **DYS456** | 16 | 16 |  |
| **Y_GATA_H4** | 12 | 12 |  |

sample 2415

PowerPlex Fusion 6C

| ***DNA locus*** | 2415  **Blood** | 2415  **Pre vasectomy semen** | 2415  **Post vasectomy seminal fluid** |
| --- | --- | --- | --- |
| **D3S1358** | 16 | 16 | 16 |
| **D1S1656** | 14/15 | 14/15 | 14/15 |
| **D2S441** | 10/16 | 10/16 | 10/16 |
| **D10S1248** | 14/15 | 14/15 | 14/15 |
| **D13S317** | 11/12 | 11/12 | 11/12 |
| **Penta E** | 7/11 | 7/11 | * |
| **D16S539** | 11/12 | 11/12 | 11/12 |
| **D18S51** | 17/18 | 17/18 | 17/18 |
| **D2S1338** | 18/20 | 18/20 | 18/20 |
| **CSF1PO** | 7/11 | 7/11 | 7/11 |
| **Penta D** | 11/12 | 11/12 | * |
| **TH01** | 7 | 7 | 7 |
| **vWA** | 17/18 | 17/18 | 17/18 |
| **D21S11** | 29/32.2 | 29/32.2 | 29/32.2 |
| **D7S820** | 10/12 | 10/12 | 10/12 |
| **D5S818** | 11/13 | 11/13 | 11 |
| **TPOX** | 8/9 | 8/9 | * |
| **D8S1179** | 13/14 | 13/14 | 13/14 |
| **D12S391** | 20/21 | 20/21 | 20/21 |
| **D19S433** | 13 | 13 | 13 |
| **SE33** | 15/31.2 | 15/31.2 | 15/31.2 |
| **D22S1045** | 17 | 17 | * |
| **DYS391** | 11 | 11 | 11 |
| **FGA** | 23 | 23 | 23 |
| **DYS576** | 17 | 17 | 17 |
| **DYS570** | 18 | 18 | * |
| **Amelogenina** | XY  (male) | XY  (male) | XY  (male) |

PowerPlex Y23 System

| ***DNA locus* Y STR** | 2415  **Blood** | 2415  **Pre vasectomy semen** | 2415  **Post vasectomy seminal fluid** |
| --- | --- | --- | --- |
| **DYS576** | 17 | 17 | * |
| **DYS389I** | 13 | 13 | * |
| **DYS448** | 20 | 20 | * |
| **DYS389II** | 29 | 29 | * |
| **DYS19** | 14 | 14 | * |
| **DYS391** | 11 | 11 | * |
| **DYS481** | 22 | 22 | * |
| **DYS549** | 14 | 14 | * |
| **DYS533** | 13 | 13 | * |
| **DYS438** | 12 | 12 | * |
| **DYS437** | 15 | 15 | * |
| **DYS570** | 18 | 18 | * |
| **DYS635** | 23 | 23 | * |
| **DYS390** | 24 | 24 | * |
| **DYS439** | 12 | 12 | * |
| **DYS392** | 14 | 14 | * |
| **DYS643** | 10 | 10 | * |
| **DYS393** | 12 | 12 | * |
| **DYS458** | 16 | 16 | * |
| **DYS385** | 11/13 | 11/13 | * |
| **DYS456** | 16 | 16 | * |
| **Y_GATA_H4** | 12 | 12 | * |

sample 2421

PowerPlex Fusion 6C

| ***DNA locus*** | 2421  **Blood** | 2421  **Pre vasectomy semen** | 2421  **Post vasectomy seminal fluid** |
| --- | --- | --- | --- |
| **D3S1358** | 15/16 | 15/16 | 15/16 |
| **D1S1656** | 14/15.3 | 14/15.3 | 14/15.3 |
| **D2S441** | 11/14 | 11/14 | 11/14 |
| **D10S1248** | 11/14 | 11/14 | 11/14 |
| **D13S317** | 11 ? | 12 | 12 |
| **Penta E** | 10 | 10 | 10 |
| **D16S539** | 9/13 | 9/13 | 9/13 |
| **D18S51** | 16/17 | 17 | 17 |
| **D2S1338** | 17/24 | 17/24 | 17/24 |
| **CSF1PO** | 12 | 12 | 12 |
| **Penta D** | 8/9 | 8/9 | 8/9 |
| **TH01** | 7/9.3? | 7/9.3 | 7/9.3 |
| **vWA** | 14/15 | 14/15 | 14/15 |
| **D21S11** | 29/32.1/37? | 29/37 | 29/37 |
| **D7S820** | 10/11 | 10/11 | 10/11 |
| **D5S818** | 9/12 | 9/12 | 9/12 |
| **TPOX** | 8/10 | 8/10 | 8/10 |
| **D8S1179** | 12 | 12 | 12 |
| **D12S391** | 16/19 | 16/19 | 16/19 |
| **D19S433** | 14/15 | 14/15 | 14/15 |
| **SE33** | 18/24.2 | 18/24.2 | 18/24.2 |
| **D22S1045** | 11/14 | 11/14 | 11/14 |
| **DYS391** | 10 | 10 | 10 |
| **FGA** | 14/17/24? | 17/24 | 17/24 |
| **DYS576** | 17 | 17 | 17 |
| **DYS570** | 17 | 17 | 17 |
| **Amelogenina** | XY  (male) | XY  (male) | XY  (male) |

PowerPlex Y23 System

| ***DNA locus* Y STR** | 2421  **Blood** | 2421  **Pre vasectomy semen** | 2421  **Post vasectomy seminal fluid** |
| --- | --- | --- | --- |
| **DYS576** | 17 | 17 | 17 |
| **DYS389I** | 13 | 13 | 13 |
| **DYS448** | 19 | 19 | 19 |
| **DYS389II** | 29 | 29 | 29 |
| **DYS19** | 15 | 15 | 15 |
| **DYS391** | 10 | 10 | 10 |
| **DYS481** | 22 | 22 | 22 |
| **DYS549** | 12 | 12 | 12 |
| **DYS533** | 12 | 12 | 12 |
| **DYS438** | 12 | 12 | 12 |
| **DYS437** | 15 | 15 | 15 |
| **DYS570** | 17 | 17 | 17 |
| **DYS635** | 23 | 23 | 23 |
| **DYS390** | 24 | 24 | 24 |
| **DYS439** | 12 | 12 | 12 |
| **DYS392** | 13 | 13 | 13 |
| **DYS643** | 10 | 10 | 10 |
| **DYS393** | 13 | 13 | 13 |
| **DYS458** | 15 | 15 | 15 |
| **DYS385** | 11/14 | 11/14 | 11/14 |
| **DYS456** | 15 | 15 | 15 |
| **Y_GATA_H4** | 12 | 12 | 12 |

sample 2423 DIFERENTE

PowerPlex Fusion 6C

| ***DNA locus*** | 2423  **Blood** | 2423  **Pre vasectomy semen** | 2423  **Post vasectomy seminal fluid** |
| --- | --- | --- | --- |
| **D3S1358** | 15/16 | 15/16 | 15/16 |
| **D1S1656** | 14/17.3 | 14/17.3 | 14/17.3 |
| **D2S441** | 10/11? | 11 | 11 |
| **D10S1248** | 14/16 | 14/16 | 14/16 |
| **D13S317** | 11/12 | 11/12 | 11/12 |
| **Penta E** | 15/16 | 15/16 | 8/15 |
| **D16S539** | 10/11 | 10/11 | 10/11 |
| **D18S51** | 12/14 | 12/14 | 12/14 |
| **D2S1338** | 17/19 | 17/19 | 17/23 |
| **CSF1PO** | 10/11 | 10/11 | 10/11 |
| **Penta D** | 12/13 | 12/13 | 12/13 |
| **TH01** | 7/8 | 7/8 | 7/8 |
| **vWA** | 16/17? | 16/17 | 16/17 |
| **D21S11** | 31/37? | 31/37 | 31/37 |
| **D7S820** | 7.3/8/12? | 8/12 | 8/11 |
| **D5S818** | 11/13 | 11/13 | 12/13 |
| **TPOX** | 8/10 | 8/10 | 9/11 |
| **D8S1179** | 11/14 | 11/14 | 11/14 |
| **D12S391** | 18/24 | 18/24 | 18/24 |
| **D19S433** | 13/15 | 13/15 | 13/15 |
| **SE33** | 26.2? | 20/26.2 | 20/26.2 |
| **D22S1045** | 16 | 16 | 15 |
| **DYS391** | 10 | 10 | 10 |
| **FGA** | 16/25.2/28? | 25.2/28 | 25.2/28 |
| **DYS576** | 17/18? | 17 | 17 |
| **DYS570** | 16? | 16 | 16 |
| **Amelogenina** | XY  (male) | XY  (male) | XY  (male) |

PowerPlex Y23 System

| ***DNA locus* Y STR** | 2423  **Blood** | 2423  **Pre vasectomy semen** | 2423  **Post vasectomy seminal fluid** |
| --- | --- | --- | --- |
| **DYS576** | 17 | 17 | 17 |
| **DYS389I** | 13 | 13 | 13 |
| **DYS448** | 19 | 19 | 19 |
| **DYS389II** | 29 | 29 | 29 |
| **DYS19** | 14 | 14 | 14 |
| **DYS391** | 10 | 10 | 10 |
| **DYS481** | 22 | 22 | 22 |
| **DYS549** | 12 | 12 | 12 |
| **DYS533** | 12 | 12 | 12 |
| **DYS438** | 12 | 12 | 12 |
| **DYS437** | 15 | 15 | 15 |
| **DYS570** | 16 | 16 | 16 |
| **DYS635** | 23 | 23 | 23 |
| **DYS390** | 24 | 24 | 24 |
| **DYS439** | 11 | 11 | 11 |
| **DYS392** | 13 | 13 | 13 |
| **DYS643** | 10 | 10 | 10 |
| **DYS393** | 13 | 13 | 13 |
| **DYS458** | 17 | 17 | 17 |
| **DYS385** | 12/14 | 12/14 | 12/14 |
| **DYS456** | 16 | 16 | 16 |
| **Y_GATA_H4** | 12 | 12 |  |

sample 2424

PowerPlex Fusion 6C

| ***DNA locus*** | 2424  **Blood** | 2424  **Pre vasectomy semen** | 2424  **Post vasectomy seminal fluid** |
| --- | --- | --- | --- |
| **D3S1358** | 16 | 16 | 16 |
| **D1S1656** | 13 | 13 | 15.3/18.3 |
| **D2S441** | 10/11 | 10/11 | * |
| **D10S1248** | 13/16 | 13/16 | * |
| **D13S317** | 9/11 | 9/11 | * |
| **Penta E** | 10/11 | 10/11 | * |
| **D16S539** | 9/12 | 9/12 | 10/12 |
| **D18S51** | 13/16 | 13/16 | 12/15 |
| **D2S1338** | 8/26 | 18/26 | * |
| **CSF1PO** | 10/12 | 10/12 | * |
| **Penta D** | 11/12 | 11/12 | * |
| **TH01** | 8/9.3 | 8/9.3 | 7/9 |
| **vWA** | 14/18 | 14/18 | 15/16 |
| **D21S11** | 30/31 | 30/31 | * |
| **D7S820** | 8/11 | 8/11 | * |
| **D5S818** | 12 | 12 | * |
| **TPOX** | 8/9 | 8/9 | * |
| **D8S1179** | 13 | 13 | 14/15 |
| **D12S391** | 19/21 | 19/21 | 18/20 |
| **D19S433** | 15.2 | 15.2 | 16.2 |
| **SE33** | 13/17 | 13/17 | * |
| **D22S1045** | 11/15 | 11/15 | * |
| **DYS391** | 10 | 10 | 10 |
| **FGA** | 23/24 | 23/24 | 19/24 |
| **DYS576** | 18 | 18 | * |
| **DYS570** | 20 | 20 | * |
| **Amelogenina** | XY  (male) | XY  (male) | XY  (male) |

PowerPlex Y23 System

| ***DNA locus* Y STR** | 2424  **Blood** | 2424  **Pre vasectomy semen** | 2424  **Post vasectomy seminal fluid** |
| --- | --- | --- | --- |
| **DYS576** | 18 | 18 | * |
| **DYS389I** | 13 | 13 | * |
| **DYS448** | 21 | 21 | * |
| **DYS389II** | 32 | 32 | * |
| **DYS19** | 15 | 15 | * |
| **DYS391** | 10 | 10 | * |
| **DYS481** | 26 | 26 | * |
| **DYS549** | 13 | 13 | * |
| **DYS533** | 12 | 12 | * |
| **DYS438** | 10 | 10 | * |
| **DYS437** | 15 | 15 | * |
| **DYS570** | 20 | 20 | * |
| **DYS635** | 21 | 21 | * |
| **DYS390** | 22 | 22 | * |
| **DYS439** | 11 | 11 | * |
| **DYS392** | 11 | 11 | * |
| **DYS643** | 12 | 12 | * |
| **DYS393** | 14 | 14 | * |
| **DYS458** | 17 | 17 | * |
| **DYS385** | 16/19 | 16/19 | * |
| **DYS456** | 14 | 14 | * |
| **Y_GATA_H4** | 11 | 11 | * |

sample 2425

PowerPlex Fusion 6C

| ***DNA locus*** | 2425  **Blood** | 2425  **Pre vasectomy semen** | 2425  **Post vasectomy seminal fluid** |
| --- | --- | --- | --- |
| **D3S1358** | 16 | 16 | 16 |
| **D1S1656** | 15 | 15 | 15 |
| **D2S441** | 10/11 | 10/11 | 10/11 |
| **D10S1248** | 14 | 14 | 14 |
| **D13S317** | 11 | 11 | 11 |
| **Penta E** | 7/13 | 7/13 | * |
| **D16S539** | 9/12 | 9/12 | 9/12 |
| **D18S51** | 15/18 | 15/18 | 15/18 |
| **D2S1338** | 16/20 | 16/20 | 16/20 |
| **CSF1PO** | 10/13 | 10/13 | 10/13 |
| **Penta D** | 10 | 10 | 10 |
| **TH01** | 7/8 | 7/8 | 7/8 |
| **vWA** | 17 | 17 | 17 |
| **D21S11** | 29 | 29 | 29 |
| **D7S820** | 8/12 | 8/12 | 8/12 |
| **D5S818** | 12 | 12 | * |
| **TPOX** | 8/9 | 8/9 | * |
| **D8S1179** | 10/13 | 10/13 | 10/13 |
| **D12S391** | 18/23 | 18/23 | 18/23 |
| **D19S433** | 14/15 | 14/15 | 14 |
| **SE33** | 17/18 | 17/18 | 17 |
| **D22S1045** | 16 | 16 | 16 |
| **DYS391** | 10 | 10 | 10 |
| **FGA** | 21/25 | 21/25 | 21/25 |
| **DYS576** | 20 | 20 | 20 |
| **DYS570** | 17 | 17 | 16/17 |
| **Amelogenina** | XY  (male) | XY  (male) | XY  (male) |

PowerPlex Y23 System

| ***DNA locus* Y STR** | 2425  **Blood** | 2425  **Pre vasectomy semen** | 2425  **Post vasectomy seminal fluid** |
| --- | --- | --- | --- |
| **DYS576** | 20 | 20 |  |
| **DYS389I** | 13 | 13 |  |
| **DYS448** | 19 | 19 |  |
| **DYS389II** | 29 | 29 |  |
| **DYS19** | 14 | 14 |  |
| **DYS391** | 10 | 10 |  |
| **DYS481** | 22 | 22 |  |
| **DYS549** | 13 | 13 |  |
| **DYS533** | 12 | 12 |  |
| **DYS438** | 12 | 12 |  |
| **DYS437** | 15 | 15 |  |
| **DYS570** | 17 | 17 |  |
| **DYS635** | 23 | 23 | 23 |
| **DYS390** | 24 | 24 |  |
| **DYS439** | 11 | 11 |  |
| **DYS392** | 13 | 13 |  |
| **DYS643** | 10 | 10 |  |
| **DYS393** | 13 | 13 | 13 |
| **DYS458** | 17 | 17 | 17 |
| **DYS385** | 12/13 | 12/13 |  |
| **DYS456** | 15 | 15 |  |
| **Y_GATA_H4** | 12 | 12 |  |

sample 2442

PowerPlex Fusion 6C

| ***DNA locus*** | 2442  **Blood** | 2442  **Pre vasectomy semen** | 2442  **Post vasectomy seminal fluid** |
| --- | --- | --- | --- |
| **D3S1358** | 15 | 15 | 15 |
| **D1S1656** | 14/17.3 | 14/17.3 | 14/17.3 |
| **D2S441** | 14 | 14 | 14 |
| **D10S1248** | 11/14 | 11/14 | 11/14 |
| **D13S317** | 9/12 | 9/12 | 9/12 |
| **Penta E** | 10/11 | 10/11 | 10/11 |
| **D16S539** | 9/14 | 9/14 | 9/14 |
| **D18S51** | 17 | 11/17 | 11/17 |
| **D2S1338** | 17 | 17 | 17 |
| **CSF1PO** | 10/11 | 10/11 | 10/11 |
| **Penta D** | 9/12 | 9/12 | 9/12 |
| **TH01** | 7/8 | 7/8 | 7/8 |
| **vWA** | 16/18 | 16/18 | 16/18 |
| **D21S11** | 28/29 | 28/29 | 28/29 |
| **D7S820** | 11 | 11 | 11 |
| **D5S818** | 11/12 | 11/12 | 11/12 |
| **TPOX** | 9/11 | 9/11 | 9/11 |
| **D8S1179** | 8/12 | 8/12 | 8/12 |
| **D12S391** | 17/19 | 17/19 | 17/19 |
| **D19S433** | 13.2/14 | 13.2/14 | 13.2/14 |
| **SE33** | 17/25.2 | 17/25.2 | 17/25.2 |
| **D22S1045** | 11/15 | 11/15 | 11 |
| **DYS391** | 10 | 10 | 10 |
| **FGA** | 24/26 | 24/26 | 24/26 |
| **DYS576** | 17 | 17 | 17 |
| **DYS570** | 19 | 19 | * |
| **Amelogenina** | XY  (male) | XY  (male) | XY  (male) |

PowerPlex Y23 System

| ***DNA locus* Y STR** | 2442  **Blood** | 2442  **Pre vasectomy semen** | 2442  **Post vasectomy seminal fluid** |
| --- | --- | --- | --- |
| **DYS576** | 17 | 17 | 17 |
| **DYS389I** | 12 | 12 | 12 |
| **DYS448** | 21 | 21 | 21 |
| **DYS389II** | 29 | 29 | 29 |
| **DYS19** | 15 | 15 | 15 |
| **DYS391** | 10 | 10 | 10 |
| **DYS481** | 22 | 22 | 22 |
| **DYS549** | 11 | 11 | 11 |
| **DYS533** | 9 | 9 | 9 |
| **DYS438** | 10 | 10 | 10 |
| **DYS437** | 16 | 16 | 16 |
| **DYS570** | 19 | 19 | 19 |
| **DYS635** | 21 | 21 | 21 |
| **DYS390** | 22 | 22 | 22 |
| **DYS439** | 12 | 12 | 12 |
| **DYS392** | 11 | 11 | 11 |
| **DYS643** |  | 11 | 11 |
| **DYS393** | 14 | 14 | 14 |
| **DYS458** | 16 | 16 | 16 |
| **DYS385** | 15 | 15 | 15 |
| **DYS456** | 18 | 18 | 18 |
| **Y_GATA_H4** |  | 12 | 12 |

sample 2445

PowerPlex Fusion 6C

| ***DNA locus*** | 2445  **Blood** | 2445  **Pre vasectomy semen** | 2445  **Post vasectomy seminal fluid** |
| --- | --- | --- | --- |
| **D3S1358** | 15/18 | 15/18 | 15/18 |
| **D1S1656** | 13/16 | 13/16 | 13/16 |
| **D2S441** | 11/13 | 11/13 | 11/13 |
| **D10S1248** | 14/16 | 14/16 | 14/16 |
| **D13S317** | 11/12 | 11/12 | 11/12 |
| **Penta E** | 13/22 | 13/22 | * |
| **D16S539** | 8/9 | 8/9 | 8/9 |
| **D18S51** | 13/19 | 13/19 | 13/19 |
| **D2S1338** | 16/17 | 16/17 | 16/17 |
| **CSF1PO** | 8/10 | 8/10 | 8/10 |
| **Penta D** | 12/13 | 12/13 | * |
| **TH01** | 6/8 | 6/8 | 6/8 |
| **vWA** | 15/17 | 15/17 | 15/17 |
| **D21S11** | 29/30 | 29/30 | 29/30 |
| **D7S820** | 10/12 | 10/12 | 10/12 |
| **D5S818** | 10/11 | 10/11 | 10/11 |
| **TPOX** | 8/11 | 8/11 | * |
| **D8S1179** | 13/14 | 13/14 | 13/14 |
| **D12S391** | 22/23 | 22/23 | 22/23 |
| **D19S433** | 14 | 14 | 14 |
| **SE33** | 15/16 | 15/16 | 15/16 |
| **D22S1045** | 15/17 | 15/17 | * |
| **DYS391** | 11 | 11 | 11 |
| **FGA** | 20/23.2 | 20/23.2 | 20/23.2 |
| **DYS576** | 18 | 18 | 18 |
| **DYS570** | 17 | 17 | 17 |
| **Amelogenina** | XY  (male) | XY  (male) | XY  (male) |

PowerPlex Y23 System

| ***DNA locus* Y STR** | 2445  **Blood** | 2445  **Pre vasectomy semen** | 2445  **Post vasectomy seminal fluid** |
| --- | --- | --- | --- |
| **DYS576** | 18 | 18 | 18 |
| **DYS389I** | 12 | 12 | 12 |
| **DYS448** | 20 | 20 | 20 |
| **DYS389II** | 29 | 29 | 29 |
| **DYS19** | 14 | 14 | 14 |
| **DYS391** | 11 | 11 | 11 |
| **DYS481** | 22 | 22 | 22 |
| **DYS549** | 12 | 12 | 12 |
| **DYS533** | 12 | 12 | 12 |
| **DYS438** | 12 | 12 | 12 |
| **DYS437** | 15 | 15 |  |
| **DYS570** | 17 | 17 | 17 |
| **DYS635** | 23 | 23 | 23 |
| **DYS390** | 25 | 25 | 25 |
| **DYS439** | 12 | 12 | 12 |
| **DYS392** | 13 | 13 | 13 |
| **DYS643** | 10 | 10 | 10 |
| **DYS393** | 13 | 13 | 13 |
| **DYS458** | 17 | 17 | 17 |
| **DYS385** | 10/14 | 10/14 | 10/14 |
| **DYS456** | 15 | 15 | 15 |
| **Y_GATA_H4** | 12 | 12 |  |

sample 2446

PowerPlex Fusion 6C

| ***DNA locus*** | 2446  **Blood** | 2446  **Pre vasectomy semen** | 2446  **Post vasectomy seminal fluid** |
| --- | --- | --- | --- |
| **D3S1358** | 15/18 | 15/18 | 15/18 |
| **D1S1656** | 14/15 | 14/15 | 14/15 |
| **D2S441** | 12/15 | 12/15 | 12/15 |
| **D10S1248** | 12/13 | 12/13 | 12/13 |
| **D13S317** | 11/12 | 11/12 | 11/12 |
| **Penta E** | 13/18 | 13/18 | 13 |
| **D16S539** | 11/13 | 11/13 | 11/13 |
| **D18S51** | 16 | 16 | 16 |
| **D2S1338** | 17/22 | 17/22 | 17/22 |
| **CSF1PO** | 10/12 | 10/12 | 10/12 |
| **Penta D** | 9/13 | 9/13 | 9/13 |
| **TH01** | 7/9 | 7/9 | 7/9 |
| **vWA** | 15/17 | 15/17 | 15/17 |
| **D21S11** | 28/31 | 28/31 | 28/31 |
| **D7S820** | 11/12 | 11/12 | 11/12 |
| **D5S818** | 12 | 12 | 12 |
| **TPOX** | 8/11 | 8/11 | 8/11 |
| **D8S1179** | 12/15 | 12/15 | 12/15 |
| **D12S391** | 19/22 | 19/22 | 19/22 |
| **D19S433** | 13.2/15 | 13.2/15 | 13.2/15 |
| **SE33** | 20/23.2 | 20/23.2 | 20/23.2 |
| **D22S1045** | 14/17 | 17 | * |
| **DYS391** | 11 | 11 | 11 |
| **FGA** | 20/22 | 20/22 | 20/22 |
| **DYS576** | 18 | 18 | 18 |
| **DYS570** | 17 | 17 | 17 |
| **Amelogenina** | XY  (male) | XY  (male) | XY  (male) |

PowerPlex Y23 System

| ***DNA locus* Y STR** | 2446  **Blood** | 2446  **Pre vasectomy semen** | 2446  **Post vasectomy seminal fluid** |
| --- | --- | --- | --- |
| **DYS576** | 18 | 18 | 18 |
| **DYS389I** | 13 | 13 | 13 |
| **DYS448** | 19 | 19 | 19 |
| **DYS389II** | 29 | 29 | 29 |
| **DYS19** | 14 | 14 | 14 |
| **DYS391** | 11 | 11 | 11 |
| **DYS481** | 22 | 22 | 22 |
| **DYS549** | 13 | 13 | 13 |
| **DYS533** | 12 | 12 | 12 |
| **DYS438** | 12 | 12 | 12 |
| **DYS437** | 15 | 15 | 15 |
| **DYS570** | 17 | 17 | 17 |
| **DYS635** | 23 | 23 | 23 |
| **DYS390** | 24 | 24 | 24 |
| **DYS439** | 12 | 12 | 12 |
| **DYS392** | 13 | 13 | 13 |
| **DYS643** | 9 | 9 | 9 |
| **DYS393** | 13 | 13 | 13 |
| **DYS458** | 16 | 16 | 16 |
| **DYS385** | 11/14 | 11/14 | 11/14 |
| **DYS456** | 15 | 15 | 15 |
| **Y_GATA_H4** | 12 | 12 | 12 |

sample 2447

PowerPlex Fusion 6C

| ***DNA locus*** | 2447  **Blood** | 2447  **Pre vasectomy semen** | 2447  **Post vasectomy seminal fluid** |
| --- | --- | --- | --- |
| **D3S1358** | 16 | 16 | 16 |
| **D1S1656** | 16/18.3 | 16/18.3 | 16/18.3 |
| **D2S441** | 11/13 | 11/13 | 11/13 |
| **D10S1248** | 13/14 | 13/14 | 13/14 |
| **D13S317** | 9/12 | 9 | 9/12 |
| **Penta E** | 5/20 | 5/20 | 5/20 |
| **D16S539** | 11/14 | 11/14 | 11/14 |
| **D18S51** | 16/18 | 16/18 | 16/18 |
| **D2S1338** | 19/21 | 19/21 | 19/21 |
| **CSF1PO** | 11/12 | 11/12 | 11/12 |
| **Penta D** | 9/13 | 9 | 9/13 |
| **TH01** | 8/9.3 | 8/9.3 | 8/9.3 |
| **vWA** | 15/16 | 15/16 | 15/16 |
| **D21S11** | 30 | 30 | 30 |
| **D7S820** | 7/8 | 7/8 | 7/8 |
| **D5S818** | 10/12 | 10/12 | 10/12 |
| **TPOX** | 8/9 | 9 | 8/9 |
| **D8S1179** | 11/15 | 11/15 | 11/15 |
| **D12S391** | 18/20 | 18/20 | 18/20 |
| **D19S433** | 13/14 | 13/14 | 13/14 |
| **SE33** | 14/26.2 | 14/26.2 | 14/26.2 |
| **D22S1045** | 16 | 16 | * |
| **DYS391** | 10 | 10 | 10 |
| **FGA** | 21/22 | 21/22 | 21/22 |
| **DYS576** | 15 | 15 | 15 |
| **DYS570** | 19 | 19 | 19 |
| **Amelogenina** | XY  (male) | XY  (male) | XY  (male) |

PowerPlex Y23 System

| ***DNA locus* Y STR** | 2447  **Blood** | 2447  **Pre vasectomy semen** | 2447  **Post vasectomy seminal fluid** |
| --- | --- | --- | --- |
| **DYS576** | 15 | 15 | 15 |
| **DYS389I** | 13 | 13 | 13 |
| **DYS448** | 21 | 21 | 21 |
| **DYS389II** | 30 | 30 | 30 |
| **DYS19** | 14 | 14 | 14 |
| **DYS391** | 10 | 10 | 10 |
| **DYS481** | 20 | 20 | 20 |
| **DYS549** | 14 | 14 | 14 |
| **DYS533** | 11 | 11 | 11 |
| **DYS438** | 9 | 9 | 9 |
| **DYS437** | 15 | 15 | 15 |
| **DYS570** | OL | 19 | 19 |
| **DYS635** | 23 | 23 | 23 |
| **DYS390** | 24 | 24 | 24 |
| **DYS439** | 12 | 12 | 12 |
| **DYS392** | 11 | 11 | 11 |
| **DYS643** | 10 | 10 | 10 |
| **DYS393** | OL | 12 | 12 |
| **DYS458** | 18 | 18 | 18 |
| **DYS385** | 14/16 | 14/16 | 14/16 |
| **DYS456** | 17 | 17 |  |
| **Y_GATA_H4** | 12 | 12 | 12 |

sample 2450

PowerPlex Fusion 6C

| ***DNA locus*** | 2450  **Blood** | 2450  **Pre vasectomy semen** | 2450  **Post vasectomy seminal fluid** |
| --- | --- | --- | --- |
| **D3S1358** | 16/17 | 16/17 | 16/17 |
| **D1S1656** | 16/16.3 | 16/16.3 | 16/16.3 |
| **D2S441** | 10/14 | 10/14 | 10/14 |
| **D10S1248** | 13/14 | 13/14 | 13/14 |
| **D13S317** | 9/11 | 9/11 | 9/11 |
| **Penta E** | 7/8 | 7/8 | * |
| **D16S539** | 9/11 | 9/11 | 9/11 |
| **D18S51** | 14/18 | 14/18 | 14/18 |
| **D2S1338** | 23/25 | 23/25 | 23/25 |
| **CSF1PO** | 7/10 | 7/10 | 7/10 |
| **Penta D** | 10/11 | 10/11 | 10/11 |
| **TH01** | 7/9.3 | 7/9.3 | 7/9.3 |
| **vWA** | 16/17 | 16/17 | 16/17 |
| **D21S11** | 28/30 | 28/30 | 28/30 |
| **D7S820** | 8/11 | 8/11 | 8/11 |
| **D5S818** | 12 | 12 | 12 |
| **TPOX** | 9/11 | 9/11 | 9/11 |
| **D8S1179** | 11/14 | 11/14 | 11/14 |
| **D12S391** | 19/23 | 19/23 | 19/23 |
| **D19S433** | 13/15 | 13/15 | 13/15 |
| **SE33** | 18 | 18 | 18 |
| **D22S1045** | 15 | 15 | 15 |
| **DYS391** | 11 | 11 | 11 |
| **FGA** | 21/22 | 21/22 | 21/22 |
| **DYS576** | 18 | 18 | 18 |
| **DYS570** | 16 | 16/17 | 16 |
| **Amelogenina** | XY  (male) | XY  (male) | XY  (male) |

PowerPlex Y23 System

| ***DNA locus* Y STR** | 2450  **Blood** | 2450  **Pre vasectomy semen** | 2450  **Post vasectomy seminal fluid** |
| --- | --- | --- | --- |
| **DYS576** | 18 | 18 |  |
| **DYS389I** | 13 | 13 | 13 |
| **DYS448** | 20 | 20 |  |
| **DYS389II** | 29 | 29 |  |
| **DYS19** | 14 | 14 |  |
| **DYS391** | 11 | 11 |  |
| **DYS481** | 23 | 23 | 23 |
| **DYS549** | 13 | 13 |  |
| **DYS533** | 13 | 13 |  |
| **DYS438** | 12 | 12 |  |
| **DYS437** | 14 | 14 |  |
| **DYS570** | 16 | 16 |  |
| **DYS635** | 23 | 23 | 23 |
| **DYS390** | 24 | 24 |  |
| **DYS439** | 12 | 12 |  |
| **DYS392** | 13 | 13 |  |
| **DYS643** | 10 | 10 |  |
| **DYS393** | 13 | 13 | 13 |
| **DYS458** | 17 | 17 | 17 |
| **DYS385** | 11/14 | 11/14 |  |
| **DYS456** | 15 | 15 |  |
| **Y_GATA_H4** | 12 | 12 |  |

sample 2451

PowerPlex Fusion 6C

| ***DNA locus*** | 2451  **Blood** | 2451  **Pre vasectomy semen** | 2451  **Post vasectomy seminal fluid** |
| --- | --- | --- | --- |
| **D3S1358** | 15/17 | 15/17 | 15/17 |
| **D1S1656** | 17.3 | 17.3 | 17.3 |
| **D2S441** | 11/14 | 11/14 | 11/14 |
| **D10S1248** | 14/15 | 14/15 | 14/15 |
| **D13S317** | 12 | 12 | 12 |
| **Penta E** | 13/15 | 13/15 | 13 |
| **D16S539** | 9/13 | 9/13 | 9/13 |
| **D18S51** | 13 | 13 | 13 |
| **D2S1338** | 23/24 | 23/24 | 23/24 |
| **CSF1PO** | 10/22 | 10 | 10 |
| **Penta D** | 12 | 2.2/12 | 2.2/12 |
| **TH01** | 8/9 | 8/9 | 8/9 |
| **vWA** | 14/16 | 14/16 | 14/16 |
| **D21S11** | 28/29 | 28/29 | 28/29 |
| **D7S820** | 11/12 | 11/12 | 11/12 |
| **D5S818** | 11/12? | 11/12 | 11/12 |
| **TPOX** | 8/10 | 8/10 | 9/11 |
| **D8S1179** | 12/13 | 12/13 | 12/13 |
| **D12S391** | 17/19 | 17/19 | 17/19 |
| **D19S433** | 15/16 | 15/16 | 15/16 |
| **SE33** | 11/15 | 11/15 | 11/15 |
| **D22S1045** | 11/15 | 11/15 | 15 |
| **DYS391** | 10 | 10 | 10 |
| **FGA** | 21/23 | 21/23 | 21/23 |
| **DYS576** | 17 | 17 | 17 |
| **DYS570** | 17 | 17 | 17 |
| **Amelogenina** | XY  (male) | XY  (male) | XY  (male) |

PowerPlex Y23 System

| ***DNA locus* Y STR** | 2451  **Blood** | 2451  **Pre vasectomy semen** | 2451  **Post vasectomy seminal fluid** |
| --- | --- | --- | --- |
| **DYS576** | 17 | 17 | 17 |
| **DYS389I** | 13 | 13 | 13 |
| **DYS448** | 19 | 19 | 19 |
| **DYS389II** | 29 | 29 | 29 |
| **DYS19** | 14 | 14 | 14 |
| **DYS391** | 10 | 10 | 10 |
| **DYS481** | 22 | 22 | 22 |
| **DYS549** | 13 | 13 | 13 |
| **DYS533** | 12 | 12 | 12 |
| **DYS438** | 12 | 12 | 12 |
| **DYS437** | 14 | 14 | 14 |
| **DYS570** | 17 | 17 | 17 |
| **DYS635** | 24 | 24 | 24 |
| **DYS390** | 24 | 24 | 24 |
| **DYS439** | 12 | 12 | 12 |
| **DYS392** | 13 | 13 | 13 |
| **DYS643** | 10 | 10 | 10 |
| **DYS393** | 13 | 13 | 13 |
| **DYS458** | 17 | 17 | 17 |
| **DYS385** | 11/14 | 11/14 | 11/14 |
| **DYS456** | 16 | 16 | 16 |
| **Y_GATA_H4** | 12 | 12 | 12 |

sample 2452

PowerPlex Fusion 6C

| ***DNA locus*** | 2452  **Blood** | 2452  **Pre vasectomy semen** | 2452  **Post vasectomy seminal fluid** |
| --- | --- | --- | --- |
| **D3S1358** | 14/15 | 14/15 | 14/15 |
| **D1S1656** | 11/12 | 11/12 | 11/12 |
| **D2S441** | 11/14 | 11/14 | 11/14 |
| **D10S1248** | 13/15 | 13/15 | 13/15 |
| **D13S317** | 9/11 | 9/11 | 9/11 |
| **Penta E** | 12/15 | 12/15 | * |
| **D16S539** | 10/12 | 10/12 | 10/12 |
| **D18S51** | 16/18 | 16/18 | 16/18 |
| **D2S1338** | 19/23 | 19/23 | 19/23 |
| **CSF1PO** | 10/12 | 10/12 | 10/12 |
| **Penta D** | 8/11 | 8/11 | * |
| **TH01** | 9.3 | 9.3 | 9.3 |
| **vWA** | 17/19 | 17/19 | 17/19 |
| **D21S11** | 29/32 | 29/32 | 29/32 |
| **D7S820** | 10/12 | 10/12 | 10/12 |
| **D5S818** | 11 | 11 | 11 |
| **TPOX** | 9 | 9 | * |
| **D8S1179** | 13 | 13 | 13 |
| **D12S391** | 16/24 | 16/24 | 16/24 |
| **D19S433** | 12/14 | 12/14 | 12/14 |
| **SE33** | 18/27.2 | 18/27.2 | 18/27.2 |
| **D22S1045** | 15/16 | 15/16 | * |
| **DYS391** | 11 | 11 | 11 |
| **FGA** | 24/25 | 24/25 | 24/25 |
| **DYS576** | 19 | 19 | 19 |
| **DYS570** | 18 | 18 | 18 |
| **Amelogenina** | XY  (male) | XY  (male) | XY  (male) |

PowerPlex Y23 System

| ***DNA locus* Y STR** | 2452  **Blood** | 2452  **Pre vasectomy semen** | 2452  **Post vasectomy seminal fluid** |
| --- | --- | --- | --- |
| **DYS576** | 19 | 19 | 19 |
| **DYS389I** | 14 | 14 | 14 |
| **DYS448** | 19 | 19 |  |
| **DYS389II** | 30 | 30 | 30 |
| **DYS19** | 14 | 14 | 14 |
| **DYS391** | 11 | 11 | 11 |
| **DYS481** | 20 | 20 | 20 |
| **DYS549** | 13 | 13 | 13 |
| **DYS533** | 13 | 13 | 13 |
| **DYS438** | 12 | 12 | 12 |
| **DYS437** | 15 | 15 |  |
| **DYS570** | 18 | 18 | 18 |
| **DYS635** | 24 | 24 | 24 |
| **DYS390** | 25 | 25 | 25 |
| **DYS439** | 11 | 11 |  |
| **DYS392** | 13 | 13 |  |
| **DYS643** | 10 | 10 |  |
| **DYS393** | 13 | 13 | 13 |
| **DYS458** | 17 | 17 | 17 |
| **DYS385** | 12/14 | 12/14 |  |
| **DYS456** | 15 | 15 | 15 |
| **Y_GATA_H4** | 12 | 12 |  |

sample 2453

PowerPlex Fusion 6C

| ***DNA locus*** | 2453  **Blood** | 2453  **Pre vasectomy semen** | 2453  **Post vasectomy seminal fluid** |
| --- | --- | --- | --- |
| **D3S1358** | 17 | 17 | 17 |
| **D1S1656** | 14/16 | 14/16 | 14/16 |
| **D2S441** | 11/14 | 11/14 | 11/14 |
| **D10S1248** | 14/16 | 14/16 | 14/16 |
| **D13S317** | 8/11 | 8/11 | 8/11 |
| **Penta E** | 7/12 | 7/12 | * |
| **D16S539** | 11 | 11 | 11 |
| **D18S51** | 16/17 | 16/17 | 16/17 |
| **D2S1338** | 19 | 19 | 19 |
| **CSF1PO** | 10 | 10 | 10 |
| **Penta D** | 5/12 | 5/12 | * |
| **TH01** | 6/8 | 6/8 | 6/8 |
| **vWA** | 18 | 18 | 18 |
| **D21S11** | 28/30 | 28/30 | 28/30 |
| **D7S820** | 9.3/12 | 9.3/12 | 9.3 |
| **D5S818** | 11/12 | 11/12 | * |
| **TPOX** | 8/9 | 8/9 | * |
| **D8S1179** | 13/14 | 13/14 | 13/14 |
| **D12S391** | 19/20 | 19/20 | 19/20 |
| **D19S433** | 13/14 | 13/14 | 13/14 |
| **SE33** | 18/30.2 | 18/30.2 | 18/30.2 |
| **D22S1045** | 10 | 10/16 | * |
| **DYS391** | 10 | 10 | 10 |
| **FGA** | 21/22 | 21/22 | 21/22 |
| **DYS576** | 17 | * | 17 |
| **DYS570** | 19 | * | * |
| **Amelogenina** | XY  (male) | XY  (male) | XY  (male) |

PowerPlex Y23 System

| ***DNA locus* Y STR** | 2453  **Blood** | 2453  **Pre vasectomy semen** | 2453  **Post vasectomy seminal fluid** |
| --- | --- | --- | --- |
| **DYS576** | 17 | 17 | * |
| **DYS389I** | 14 | 14 | * |
| **DYS448** | 21 | 21 | * |
| **DYS389II** | 30 | 30 | * |
| **DYS19** | 14 | 14 | * |
| **DYS391** | 10 | 10 | * |
| **DYS481** | 26 | 26 | * |
| **DYS549** | 11 | 11 | * |
| **DYS533** | 12 | 12 | * |
| **DYS438** | 10 | 10 | * |
| **DYS437** | 14 | 14 | * |
| **DYS570** | 19 | 19 | * |
| **DYS635** | 22 | 22 | * |
| **DYS390** | 25 | 25 | * |
| **DYS439** | 11 | 11 | * |
| **DYS392** | 11 | 11 | * |
| **DYS643** | 9 | 9 | * |
| **DYS393** | 12 | 12 | * |
| **DYS458** | 22.2 | 22.2 | * |
| **DYS385** | 12/18 | 12/18 | * |
| **DYS456** | 14 | 14 | * |
| **Y_GATA_H4** | 11 | 11 | * |

sample 2454

PowerPlex Fusion 6C

| ***DNA locus*** | 2454  **Blood** | 2454  **Pre vasectomy semen** | 2454  **Post vasectomy seminal fluid** |
| --- | --- | --- | --- |
| **D3S1358** | 16/17 | 16/17 | 16/17 |
| **D1S1656** | 14/15.3 | 14/15.3 | 14/15.3 |
| **D2S441** | 11/11.3 | 11/11.3 | 11/11.3 |
| **D10S1248** | 13/17 | 13/17 | 13/17 |
| **D13S317** | 11/12 | 11/12 | 11/12 |
| **Penta E** | 12/13 | 12/13 | * |
| **D16S539** | 11/12 | 11/12 | 11/12 |
| **D18S51** | 14/15 | 14/15 | 14/15 |
| **D2S1338** | 17/25 | 17/25 | 17/25 |
| **CSF1PO** | 10/12 | 10/12 | 10/12 |
| **Penta D** | 12/13 | 12/13 | 12/13 |
| **TH01** | 7/9.3 | 7/9.3 | 7/9.3 |
| **vWA** | 15/19 | 15/19 | 15/19 |
| **D21S11** | 31/32.2 | 31/32.2 | 31/32.2 |
| **D7S820** | 8/11 | 8/11 | 8/11 |
| **D5S818** | 12 | 12 | 12 |
| **TPOX** | 8/11 | 8/11 | * |
| **D8S1179** | 12/13 | 12/13 | 12/13 |
| **D12S391** | 18/23 | 18/23 | 18/23 |
| **D19S433** | 12.2/13 | 12.2/13 | 12.2/13 |
| **SE33** | 22.2/27.2 | 22.2/27.2 | 22.2/27.2 |
| **D22S1045** | 15/17 | 15/17 | * |
| **DYS391** | 10 | 10 | 10 |
| **FGA** | 20/21 | 20/21 | 20/21 |
| **DYS576** | 17 | 17 | 17 |
| **DYS570** | 19 | 19 | 19 |
| **Amelogenina** | XY  (male) | XY  (male) | XY  (male) |

PowerPlex Y23 System

| ***DNA locus* Y STR** | 2454  **Blood** | 2454  **Pre vasectomy semen** | 2454  **Post vasectomy seminal fluid** |
| --- | --- | --- | --- |
| **DYS576** | 17 | 17 |  |
| **DYS389I** | 13 | 13 | 13 |
| **DYS448** | 20 | 20 |  |
| **DYS389II** | 30 | 30 |  |
| **DYS19** | 13 | 13 |  |
| **DYS391** | 10 | 10 |  |
| **DYS481** | 24 | 24 |  |
| **DYS549** | 12 | 12 |  |
| **DYS533** | 12 | 12 |  |
| **DYS438** | 10 | 10 |  |
| **DYS437** | 14 | 14 |  |
| **DYS570** | 19 | 19 |  |
| **DYS635** | 21 | 21 |  |
| **DYS390** | 23 | 23 |  |
| **DYS439** | 12 | 12 |  |
| **DYS392** | 11 | 11 |  |
| **DYS643** | 13 | 13 |  |
| **DYS393** | 13 | 13 | 13 |
| **DYS458** | 16 | 16 |  |
| **DYS385** | 16 | 16 |  |
| **DYS456** | 17 | 17 |  |
| **Y_GATA_H4** | 11 | 11 |  |

sample 2455

PowerPlex Fusion 6C

| ***DNA locus*** | 2455  **Blood** | 2455  **Pre vasectomy semen** | 2455  **Post vasectomy seminal fluid** |
| --- | --- | --- | --- |
| **D3S1358** | 17/18 | 17/18 | 17/18 |
| **D1S1656** | 17.3/18.3 | 17.3/18.3 | 17.3/18.3 |
| **D2S441** | 10/13 | 10/13 | 10/13 |
| **D10S1248** | 14/15 | 14/15 | 14/15 |
| **D13S317** | 12/13 | 12/13 | 12/13 |
| **Penta E** | 9/10 | 9/10 | 9/10 |
| **D16S539** | 13 | 13 | 13 |
| **D18S51** | 12/18 | 12/18 | 12/18 |
| **D2S1338** | 18/23 | 18/23 | 18/23 |
| **CSF1PO** | 11/12 | 11/12 | 11/12 |
| **Penta D** | 9 | 9 | 9 |
| **TH01** | 6/7 | 6/7 | 6/7 |
| **vWA** | 16/17 | 16/17 | 16/17 |
| **D21S11** | 28/30 | 28/30 | 28/30 |
| **D7S820** | 12/13 | 12/13 | 12/13 |
| **D5S818** | 9/12 | 9/12 | 9/12 |
| **TPOX** | 10/11 | 10/11 | 10/11 |
| **D8S1179** | 13 | 13 | 13 |
| **D12S391** | 20/23 | 20/23 | 20/23 |
| **D19S433** | 12/13 | 12/13 | 12/13 |
| **SE33** | 15/17 | 15/17 | 15/17 |
| **D22S1045** | 16 | 16 | 16 |
| **DYS391** | 10 | 10 | 10 |
| **FGA** | 19/26 | 19/26 | 19/26 |
| **DYS576** | 17 | 17 | 17 |
| **DYS570** | 19 | 19 | 19 |
| **Amelogenina** | XY  (male) | XY  (male) | XY  (male) |

PowerPlex Y23 System

| ***DNA locus* Y STR** | 2455  **Blood** | 2455  **Pre vasectomy semen** | 2455  **Post vasectomy seminal fluid** |
| --- | --- | --- | --- |
| **DYS576** | 17 | 17 | 17 |
| **DYS389I** | 13 | 13 | 13 |
| **DYS448** | 20 | 20 | 20 |
| **DYS389II** | 30 | 30 | 30 |
| **DYS19** | 15 | 15 | 15 |
| **DYS391** | 10 | 10 | 10 |
| **DYS481** | 26 | 26 | 26 |
| **DYS549** | 11 | 11 | 11 |
| **DYS533** | 12 | 12 | 12 |
| **DYS438** | 11 | 11 | 11 |
| **DYS437** | 14 | 14 | 14 |
| **DYS570** | 19 | 19 | 19 |
| **DYS635** | 21 | 21 | 21 |
| **DYS390** | 21 | 21 | 21 |
| **DYS439** | 12 | 12 | 12 |
| **DYS392** | 11 | 11 | 11 |
| **DYS643** | 12 | 12 | 12 |
| **DYS393** | 14 | 14 | 14 |
| **DYS458** | 18 | 18 | 18 |
| **DYS385** | 16 | 16 | 16 |
| **DYS456** | 15 | 15 | 15 |
| **Y_GATA_H4** | 10 | 10 | 10 |

sample 2456

PowerPlex Fusion 6C

| ***DNA locus*** | 2456  **Blood** | 2456  **Pre vasectomy semen** | 2456  **Post vasectomy seminal fluid** |
| --- | --- | --- | --- |
| **D3S1358** | 14/16 | 14/16 | 14/16 |
| **D1S1656** | 14 | 14 | 14 |
| **D2S441** | 11/11.3 | 11/11.3 | 11/11.3 |
| **D10S1248** | 13/16 | 13/16 | 13/16 |
| **D13S317** | 10/12 | 10/12 | 10/12 |
| **Penta E** | 12/17 | 12/17 | * |
| **D16S539** | 11/12 | 11/12 | 11/12 |
| **D18S51** | 14/15 | 14/15 | 14/15 |
| **D2S1338** | 16/20 | 16/20 | * |
| **CSF1PO** | 12 | 12 | 12 |
| **Penta D** | 9/12 | 9/12 | * |
| **TH01** | 8/9 | 8/9 | 8/9 |
| **vWA** | 15/17 | 15/17 | 15/17 |
| **D21S11** | 30/30.2 | 30/30.2 | 30 |
| **D7S820** | 11/14 | 11/14 | 11/14 |
| **D5S818** | 11 | 11 | 11 |
| **TPOX** | 10/11 | 11 | * |
| **D8S1179** | 15 | 15 | 15 |
| **D12S391** | 17/18 | 17/18 | 17/18 |
| **D19S433** | 14/15 | 14/15 | 14/15 |
| **SE33** | 18/21 | 18/21 | 18/21 |
| **D22S1045** | 16/17 | 16/17 | * |
| **DYS391** | 10 | 10 | 10 |
| **FGA** | 20/24 | 20/24 | 20/24 |
| **DYS576** | 16 | 16 | 16 |
| **DYS570** | 17 | 17 | * |
| **Amelogenina** | XY  (male) | XY  (male) | XY  (male) |

PowerPlex Y23 System

| ***DNA locus* Y STR** | 2456  **Blood** | 2456  **Pre vasectomy semen** | 2456  **Post vasectomy seminal fluid** |
| --- | --- | --- | --- |
| **DYS576** | 16 | 16 |  |
| **DYS389I** | 14 | 14 | 14 |
| **DYS448** | 20 | 20 |  |
| **DYS389II** | 29 | 29 | 29 |
| **DYS19** | 14 | 14 | 14 |
| **DYS391** | 10 | 10 | 10 |
| **DYS481** | 22 | 22 | 22 |
| **DYS549** | 13 | 13 |  |
| **DYS533** | 12 | 12 | 12 |
| **DYS438** | 9 | 9 | 9 |
| **DYS437** | 14 | 14 |  |
| **DYS570** | 17 | 17 | 17 |
| **DYS635** | 21 | 21 | 21 |
| **DYS390** | 23 | 23 | 23 |
| **DYS439** | 11 | 11 |  |
| **DYS392** | 13 | 13 |  |
| **DYS643** | 10 | 10 |  |
| **DYS393** | 12 | 12 | 12 |
| **DYS458** | 18 | 18 | 18 |
| **DYS385** | 14/16 | 14/16 |  |
| **DYS456** | 15 | 15 |  |
| **Y_GATA_H4** | 10 | 10 |  |

sample 2457

PowerPlex Fusion 6C

| ***DNA locus*** | 2457  **Blood** | 2457  **Pre vasectomy semen** | 2457  **Post vasectomy seminal fluid** |
| --- | --- | --- | --- |
| **D3S1358** | 15/16 | 15/16 | 15/16 |
| **D1S1656** | 11/15 | 11/15 | 11/15 |
| **D2S441** | 11/11.3 | 11/11.3 | 11/11.3 |
| **D10S1248** | 13/15 | 13/15 | 13/15 |
| **D13S317** | 11/12 | 12 | 12 |
| **Penta E** | 11/15 | 11/15 | 11/15 |
| **D16S539** | 11/12 | 11/12 | 11/12 |
| **D18S51** | 13/17 | 13/17 | 13/17 |
| **D2S1338** | 17 | 17 | 17 |
| **CSF1PO** | 11/12 | 11/12 | 11/12 |
| **Penta D** | 12/14 | 12/14 | 12/14 |
| **TH01** | 6/9.3 | 6/9.3 | 6/9.3 |
| **vWA** | 16/18 | 16/18 | 16/18 |
| **D21S11** | 29/30/30.2/31.2? | 29/31.2 | 29/31.2 |
| **D7S820** | 10 | 10 | 10 |
| **D5S818** | 12/13? | 12/13 | 12/13 |
| **TPOX** | 8 | 8 | 8 |
| **D8S1179** | 14 | 14 | 14 |
| **D12S391** | 19/22 | 19/22 | 19/22 |
| **D19S433** | 12/15.2 | 12/15.2 | 12/15.2 |
| **SE33** | 17/27.2 | 17/27.2 | 17/27.2 |
| **D22S1045** | 15/16 | 15 | 15/16 |
| **DYS391** | 10/11? | 11 | 11 |
| **FGA** | 17/20? | 20 | 20 |
| **DYS576** | 15/18? | 18 | 18 |
| **DYS570** | 14.2/18? | 18 | 18 |
| **Amelogenina** | XY  (male) | XY  (male) | XY  (male) |

PowerPlex Y23 System

| ***DNA locus* Y STR** | 2457  **Blood** | 2457  **Pre vasectomy semen** | 2457  **Post vasectomy seminal fluid** |
| --- | --- | --- | --- |
| **DYS576** | 18 | 18 | 18 |
| **DYS389I** | 13 | 13 | 13 |
| **1DYS448** | 18 | 18 | 18 |
| **DYS389II** | 29 | 29 | 29 |
| **DYS19** | 14 | 14 | 14 |
| **DYS391** | 11 | 11 | 11 |
| **DYS481** | 22 | 22 | 22 |
| **DYS549** | 12 | 12 | 12 |
| **DYS533** | 12 | 12 | 12 |
| **DYS438** | 12 | 12 | 12 |
| **DYS437** | 14 | 14 | 14 |
| **DYS570** | 18 | 18 | 18 |
| **DYS635** | 23 | 23 | 23 |
| **DYS390** | 24 | 24 | 24 |
| **DYS439** | 12 | 12 | 12 |
| **DYS392** | 13 | 13 | 13 |
| **DYS643** | 10 | 10 | 10 |
| **DYS393** | 13 | 13 | 13 |
| **DYS458** | 16 | 16 | 16 |
| **DYS385** | 11/14 | 11/14 | 11/14 |
| **DYS456** | 16 | 16 | 16 |
| **Y_GATA_H4** | 12 | 12 | 12 |

sample 2458

PowerPlex Fusion 6C

| ***DNA locus*** | 2458  **Blood** | 2458  **Pre vasectomy semen** | 2458  **Post vasectomy seminal fluid** |
| --- | --- | --- | --- |
| **D3S1358** | 15/18 | 15/18 | 15/18 |
| **D1S1656** | 13/15 | 13/15 | 13/15 |
| **D2S441** | 10/11 | 10/11 | 10/11 |
| **D10S1248** | 13/14 | 13/14 | 13/14 |
| **D13S317** | 11/13 | 11/13 | * |
| **Penta E** | 10/12 | 10/12 | * |
| **D16S539** | 9 | 9 | 9 |
| **D18S51** | 12/16 | 12/16 | 12/16 |
| **D2S1338** | 17/22 | 17/22 | 17/22 |
| **CSF1PO** | 10/11 | 10 | 11 |
| **Penta D** | 11/15 | 11/15 | * |
| **TH01** | 8 | 8 | 8 |
| **vWA** | 14/15 | 14/15 | 14/15 |
| **D21S11** | 30/32.2 | 30/32.2 | * |
| **D7S820** | 10 | 10 | * |
| **D5S818** | 11/12 | 11/12 | * |
| **TPOX** | 8 | 8 | * |
| **D8S1179** | 14/15 | 14/15 | 14/15 |
| **D12S391** | 18/18.3 | 18/18.3 | 18/18.3 |
| **D19S433** | 14/15 | 14/15 | 14 |
| **SE33** | 27.2/32.2 | 27.2/32.2 | * |
| **D22S1045** | 15/16 | 15/16 | * |
| **DYS391** | 10 | 10 | 10 |
| **FGA** | 20/21 | 20/21 | 20 |
| **DYS576** | 16 | 16 | * |
| **DYS570** | 17 | 17 | * |
| **Amelogenina** | XY  (male) | XY  (male) | XY  (male) |

PowerPlex Y23 System

| ***DNA locus* Y STR** | 2458  **Blood** | 2458  **Pre vasectomy semen** | 2458  **Post vasectomy seminal fluid** |
| --- | --- | --- | --- |
| **DYS576** | 16 | 16 |  |
| **DYS389I** | 12 | 12 | 12 |
| **DYS448** | 19 | 19 |  |
| **DYS389II** | 28 | 28 |  |
| **DYS19** | 14 | 14 |  |
| **DYS391** | 10 | 10 | 10 |
| **DYS481** | 22 | 22 | 22 |
| **DYS549** | 14 | 14 |  |
| **DYS533** | 12 | 12 |  |
| **DYS438** | 12 | 12 |  |
| **DYS437** | 15 | 15 |  |
| **DYS570** | 17 | 17 |  |
| **DYS635** | 24 | 24 | 24 |
| **DYS390** | 24 | 24 | 24 |
| **DYS439** | 13 | 13 |  |
| **DYS392** | 13 | 13 |  |
| **DYS643** | 10 | 10 |  |
| **DYS393** | 13 | 13 | 13 |
| **DYS458** | 18 | 18 | 18 |
| **DYS385** | 11/14 | 11/14 |  |
| **DYS456** | 16 | 16 |  |
| **Y_GATA_H4** | 12 | 12 |  |

sample 2459

PowerPlex Fusion 6C

| ***DNA locus*** | 2459  **Blood** | 2459  **Pre vasectomy semen** | 2459  **Post vasectomy seminal fluid** |
| --- | --- | --- | --- |
| **D3S1358** | 15/19 | 15/19 | 15/19 |
| **D1S1656** | 12/14 | 12/14 | 12/14 |
| **D2S441** | 14 | 14 | 14 |
| **D10S1248** | 14/15 | 14/15 | 14 |
| **D13S317** | 8/14 | 8/14 | * |
| **Penta E** | 11/12 | 11/12 | * |
| **D16S539** | 11/12 | 11/12 | 11/12 |
| **D18S51** | 15/17 | 15/17 | 15/17 |
| **D2S1338** | 17/19 | 17/19 | 17/19 |
| **CSF1PO** | 11/13 | 11/13 | 11/13 |
| **Penta D** | 12 | 12 | * |
| **TH01** | 9.3 | 9.3 | 9.3 |
| **vWA** | 16/17 | 16/17 | 16/17 |
| **D21S11** | 28/29 | 28/29 | 28/29 |
| **D7S820** | 9/11 | 9/11 | 9 |
| **D5S818** | 9/11 | 9/11 | * |
| **TPOX** | 8/9 | 8/9 | 8 |
| **D8S1179** | 14/15 | 14/15 | 14/15 |
| **D12S391** | 17/19 | 17/19 | 17/19 |
| **D19S433** | 13/15 | 13/15 | 14/15 |
| **SE33** | 16/18 | 16/18 | 18/22/22.2 |
| **D22S1045** | 12/17 | 12/17 | 16 |
| **DYS391** | 10 | 10 | 10 |
| **FGA** | 24 | 24 | 24/OL |
| **DYS576** | 17 | 17 | * |
| **DYS570** | 17 | 17 | * |
| **Amelogenina** | XY  (male) | XY  (male) | XY  (male) |

PowerPlex Y23 System

| ***DNA locus* Y STR** | 2459  **Blood** | 2459  **Pre vasectomy semen** | 2459  **Post vasectomy seminal fluid** |
| --- | --- | --- | --- |
| **DYS576** | * | 17 |  |
| **DYS389I** | * | 13 | 13 |
| **DYS448** | * | 20 |  |
| **DYS389II** | * | 29 |  |
| **DYS19** | * | 14 |  |
| **DYS391** | * | 10 |  |
| **DYS481** | * | 23 | 23 |
| **DYS549** | * | 13 |  |
| **DYS533** | * | 13 |  |
| **DYS438** | * | 9 |  |
| **DYS437** | * | 14 |  |
| **DYS570** | * | 17 |  |
| **DYS635** | * | 23 |  |
| **DYS390** | * | 23 |  |
| **DYS439** | * | 12 |  |
| **DYS392** | * | 11 |  |
| **DYS643** | * | 9 |  |
| **DYS393** | * | 12 | 12 |
| **DYS458** | * | 19 | 19/20.2 |
| **DYS385** | * | 13/16 |  |
| **DYS456** | * | 15 |  |
| **Y_GATA_H4** | * | 11 |  |

sample 2460

PowerPlex Fusion 6C

| ***DNA locus*** | 2460  **Blood** | 2460  **Pre vasectomy semen** | 2460  **Post vasectomy seminal fluid** |
| --- | --- | --- | --- |
| **D3S1358** | 15/18 | 15/18 | 15 |
| **D1S1656** | 12/15 | 12/15 | 12/15 |
| **D2S441** | 12/13.3 | 12/13.3 | 13.3 |
| **D10S1248** | 11/14 | 11/14 | 14 |
| **D13S317** | 11/12 | 11/12 | * |
| **Penta E** | 9/14 | 9/14 | * |
| **D16S539** | 9/11 | 9/11 | 11 |
| **D18S51** | 13/19 | 13/19 | 13/19 |
| **D2S1338** | 18/20 | 18/20 | * |
| **CSF1PO** | 10 | 10 | 10 |
| **Penta D** | 2.2/8 | 2.2/8 | * |
| **TH01** | 7/8 | 7/8 | 7/8 |
| **vWA** | 15/18 | 15/18 | 15 |
| **D21S11** | 27/30 | 27/30 | * |
| **D7S820** | 8/10 | 8/10 | * |
| **D5S818** | 12 | 12 | * |
| **TPOX** | 9/11 | 11 | * |
| **D8S1179** | 13/15 | 13/15 | 13/15 |
| **D12S391** | 18/22 | 18/22 | * |
| **D19S433** | 16.2 | 16.2 | * |
| **SE33** | 17/27.2 | 17/27.2 | * |
| **D22S1045** | 11/15 | 11/15 | * |
| **DYS391** | 10 | 10 | 10 |
| **FGA** | 22/23 | 22/23 | 22 |
| **DYS576** | 15 | 15 | * |
| **DYS570** | 17 | 17 | * |
| **Amelogenina** | XY  (male) | XY  (male) | XY  (male) |

PowerPlex Y23 System

| ***DNA locus* Y STR** | 2460  **Blood** | 2460  **Pre vasectomy semen** | 2460  **Post vasectomy seminal fluid** |
| --- | --- | --- | --- |
| **DYS576** | 15 | 15 |  |
| **DYS389I** | 13 | 13 | 13 |
| **DYS448** | 19 | 19 |  |
| **DYS389II** | 31 | 31 |  |
| **DYS19** | 16 | 16 |  |
| **DYS391** | 10 | 10 |  |
| **DYS481** | 25 | 25 |  |
| **DYS549** | 13 | 13 |  |
| **DYS533** | 12 | 12 |  |
| **DYS438** | 9 | 9 |  |
| **DYS437** | 14 | 14 |  |
| **DYS570** | 17 | 17 |  |
| **DYS635** | 21 | 21 | 21 |
| **DYS390** | 23 | 23 |  |
| **DYS439** | 11 | 11 |  |
| **DYS392** | 14 | 14 |  |
| **DYS643** | 10 | 10 |  |
| **DYS393** | 14 | 14 | 14 |
| **DYS458** | 17 | 17 | 17 |
| **DYS385** | 15/18 | 15/18 |  |
| **DYS456** | 15 | 15 |  |
| **Y_GATA_H4** | 12 | 12 |  |

sample 2461

PowerPlex Fusion 6C

| ***DNA locus*** | 2461  **Blood** | 2461  **Pre vasectomy semen** | 2461  **Post vasectomy seminal fluid** |
| --- | --- | --- | --- |
| **D3S1358** | 16/17 | 16/17 | 16/17 |
| **D1S1656** | 11/12 | 11/12 | 11/12 |
| **D2S441** | 12/15 | 12/15 | 12/15 |
| **D10S1248** | 14 | 14 | 14 |
| **D13S317** | 8/12 | 8/12 | 8/12 |
| **Penta E** | 12 | 12 | * |
| **D16S539** | 12/13 | 12/13 | 12/13 |
| **D18S51** | 16/17 | 16/17 | 16/17 |
| **D2S1338** | 17/20 | 17/20 | 17/20 |
| **CSF1PO** | 10/13 | 10/13 | 10/13 |
| **Penta D** | 11/14 | 11/14 | 14 |
| **TH01** | 6/8 | 6/8 | 6/8 |
| **vWA** | 15 | 15 | 15 |
| **D21S11** | 30/36 | 30/36 | 30/36 |
| **D7S820** | 11/12 | 11/12 | 11/12 |
| **D5S818** | 10/11 | 10/11 | 10/11 |
| **TPOX** | 8/11 | 8/11 | * |
| **D8S1179** | 13/15 | 13/15 | 13/15 |
| **D12S391** | 21/26 | 21/26 | 21/26 |
| **D19S433** | 14/14.2 | 14/14.2 | 14/14.2 |
| **SE33** | 19.2/26.2 | 19.2/26.2 | 19.2/26.2 |
| **D22S1045** | 11/15 | 11/15 | 15 |
| **DYS391** | 11 | 11 | 11 |
| **FGA** | 23/25 | 23/25 | 23/25 |
| **DYS576** | 16 | 16 | 16 |
| **DYS570** | 17 | 17 | * |
| **Amelogenina** | XY  (male) | XY  (male) | XY  (male) |

PowerPlex Y23 System

| ***DNA locus* Y STR** | 2461  **Blood** | 2461  **Pre vasectomy semen** | 2461  **Post vasectomy seminal fluid** |
| --- | --- | --- | --- |
| **DYS576** | 16 | 16 |  |
| **DYS389I** | 13 | 13 | 13 |
| **DYS448** | 19 | 19 |  |
| **DYS389II** | 29 | 29 |  |
| **DYS19** | 16 | 16 |  |
| **DYS391** | 11 | 11 | 11 |
| **DYS481** | 21 | 21 | 21 |
| **DYS549** | 13 | 13 |  |
| **DYS533** | 12 | 12 | 12 |
| **DYS438** | 9 | 9 | 9 |
| **DYS437** | 15 | 15 |  |
| **DYS570** | 17 | 17 | 17 |
| **DYS635** | 24 | 24 | 24 |
| **DYS390** | 23 | 23 |  |
| **DYS439** | 11 | 11 |  |
| **DYS392** | 11 | 11 |  |
| **DYS643** | 11 | 11 |  |
| **DYS393** | 12 | 12 | 12 |
| **DYS458** | 15 | 15 | 15 |
| **DYS385** | 12/16 | 12/16 |  |
| **DYS456** | 15 | 15 |  |
| **Y_GATA_H4** | 11 | 11 |  |

sample 2471

PowerPlex Fusion 6C

| ***DNA locus*** | 2471  **Blood** | 2471  **Pre vasectomy semen** | 2471  **Post vasectomy seminal fluid** |
| --- | --- | --- | --- |
| **D3S1358** | 15/17 | 15/17 | 15/17 |
| **D1S1656** | 12/15 | 12/15 | 12/15 |
| **D2S441** | 10/14 | 10/14 | 10/14 |
| **D10S1248** | 14/15 | 14/15 | 14/15 |
| **D13S317** | 9/11 | 9/11 | 9/11 |
| **Penta E** | 12 | 12 | 12/18 |
| **D16S539** | 9/11 | 9/11 | 9/11 |
| **D18S51** | 13/17 | 13/17 | 13/17 |
| **D2S1338** | 18/22 | 18/22 | 18/22 |
| **CSF1PO** | 12 | 12 | 12 |
| **Penta D** | 12/14 | 12/14 | 12/14 |
| **TH01** | 6/7 | 6/7 | 6/7 |
| **vWA** | 14/16 | 14/16 | 14/16 |
| **D21S11** | 27/28 | 27/28 | 27/28 |
| **D7S820** | 10/11 | 10/11 | 10/11 |
| **D5S818** | 9/10 | 9/10 | 9/10 |
| **TPOX** | 11/12 | 11 | 11/12 |
| **D8S1179** | 13/14 | 13/14 | 13/14 |
| **D12S391** | 18/22 | 18/22 | 18/22 |
| **D19S433** | 14/15 | 14/15 | 14/15 |
| **SE33** | 14/17 | 14/17 | 14/17 |
| **D22S1045** | 11/16 | 11/16 | 11/16 |
| **DYS391** | 10 | 10 | 10 |
| **FGA** | 19/24 | 19/24 | 19/24 |
| **DYS576** | 16 | 16 | 16 |
| **DYS570** | 19 | 19 | 19 |
| **Amelogenina** | XY  (male) | XY  (male) | XY  (male) |

PowerPlex Y23 System

| ***DNA locus* Y STR** | 2471  **Blood** | 2471  **Pre vasectomy semen** | 2471  **Post vasectomy seminal fluid** |
| --- | --- | --- | --- |
| **DYS576** | 16 | 16 | 16 |
| **DYS389I** | 13 | 13 | 13 |
| **DYS448** | 19 | 19 | 19 |
| **DYS389II** | 30 | 30 | 30 |
| **DYS19** | 13 | 13 | 13 |
| **DYS391** | 10 | 10 | 10 |
| **DYS481** | 21 | 21 | 21 |
| **DYS549** | 13 | 13 | 13 |
| **DYS533** | 13 | 13 | 13 |
| **DYS438** | 10 | 10 | 10 |
| **DYS437** | 14 | 14 | 14 |
| **DYS570** | 19 | 19 | 19 |
| **DYS635** | 22 | 22 | 22 |
| **DYS390** | 24 | 24 | 24 |
| **DYS439** | 11 | 11 | 11 |
| **DYS392** | 11 | 11 | 11 |
| **DYS643** | 12 | 12 | 12 |
| **DYS393** | 13 | 13 | 13 |
| **DYS458** | 12 | 12 | 12 |
| **DYS385** | 16/19 | 16/19 | 16/19 |
| **DYS456** | 16 | 16 | 16 |
| **Y_GATA_H4** | 12 | 12 | 12 |

sample 2472

PowerPlex Fusion 6C

| ***DNA locus*** | 2472  **Blood** | 2472  **Pre vasectomy semen** | 2472  **Post vasectomy seminal fluid** |
| --- | --- | --- | --- |
| **D3S1358** | 15 | 15 | 15 |
| **D1S1656** | 13/19.3 | 13/19.3 | 13/19.3 |
| **D2S441** | 11/12 | 11/12 | 11/12 |
| **D10S1248** | 12/13 | 12/13 | 12/13 |
| **D13S317** | 11 | 11 | 11 |
| **Penta E** | 7/12 | 7/12 | 7/12 |
| **D16S539** | 9/10 | 9/10 | 9/10 |
| **D18S51** | 17/20 | 17/20 | 17/20 |
| **D2S1338** | 17/25 | 17/25 | 17/25 |
| **CSF1PO** | 11 | 11 | 11 |
| **Penta D** | 12/13 | 12/13 | 13 |
| **TH01** | 6/7 | 6/7 | 6/7 |
| **vWA** | 15/18 | 15/18 | 15/18 |
| **D21S11** | 28/30 | 28/30 | 28/30 |
| **D7S820** | 10/11 | 10/11 | 10/11 |
| **D5S818** | 13 | 13 | 13 |
| **TPOX** | 9/11 | 9/11 | 9/11 |
| **D8S1179** | 10/15 | 10/15 | 10/15 |
| **D12S391** | 15/21 | 15/21 | 15/21 |
| **D19S433** | 13 | 13 | 13 |
| **SE33** | 25.2/31.2 | 25.2/31.2 | 25.2/31.2 |
| **D22S1045** | 11/16 | 11/16 | 11 |
| **DYS391** | 10 | 10 | 10 |
| **FGA** | 22 | 22 | 22 |
| **DYS576** | 17 | 17 | 17 |
| **DYS570** | 17 | 17 | 17 |
| **Amelogenina** | XY  (male) | XY  (male) | XY  (male) |

PowerPlex Y23 System

| ***DNA locus* Y STR** | 2472  **Blood** | 2472  **Pre vasectomy semen** | 2472  **Post vasectomy seminal fluid** |
| --- | --- | --- | --- |
| **DYS576** | 17 | 17 | 17 |
| **DYS389I** | 13 | 13 | 13 |
| **DYS448** | 21 | 21 | 21 |
| **DYS389II** | 31 | 31 | 31 |
| **DYS19** | 15 | 15 | 15 |
| **DYS391** | 10 | 10 | 10 |
| **DYS481** | 25 | 25 | 25 |
| **DYS549** | 13 | 13 | 13 |
| **DYS533** | 11 | 11 | 11 |
| **DYS438** | 10 | 10 | 10 |
| **DYS437** | 14 | 14 | 14 |
| **DYS570** | 17 | 17 | 17 |
| **DYS635** | 21 | 21 | 21 |
| **DYS390** | 21 | 21 | 21 |
| **DYS439** | 12 | 12 | 12 |
| **DYS392** | 11 | 11 | 11 |
| **DYS643** | 12 | 12 | 12 |
| **DYS393** | 15 | 15 | 15 |
| **DYS458** | 17 | 17 | 17 |
| **DYS385** | 17/18 | 17/18 | 17/18 |
| **DYS456** | 15 | 15 | 15 |
| **Y_GATA_H4** | 11 | 11 | 11 |

sample 2473

PowerPlex Fusion 6C

| ***DNA locus*** | 2473  **Blood** | 2473  **Pre vasectomy semen** | 2473  **Post vasectomy seminal fluid** |
| --- | --- | --- | --- |
| **D3S1358** | 16/17 | 16/17 | 16/17 |
| **D1S1656** | 19.3 | 19.3 | * |
| **D2S441** | 11/14 | 11/14 | 11 |
| **D10S1248** | 14/16 | 14/16 | * |
| **D13S317** | 12/13 | 12/13 | * |
| **Penta E** | 12/15 | 12/15 | * |
| **D16S539** | 11/12 | 11/12 | 11/12 |
| **D18S51** | 16/20 | 16/20 | 20 |
| **D2S1338** | 16/25 | 16/25 | * |
| **CSF1PO** | 10/12 | 10/12 | * |
| **Penta D** | 9/13 | 9/13 | * |
| **TH01** | 6/9 | 6/9 | 6 |
| **vWA** | 14/15 | 14/15 | 14 |
| **D21S11** | 28/31.2 | 28/31.2 | * |
| **D7S820** | 10/12 | 10/12 | * |
| **D5S818** | 9/12 | 9/12 | * |
| **TPOX** | 8 | 8 | * |
| **D8S1179** | 13 | 13 | 13 |
| **D12S391** | 18/21 | 18/21 | 18/21 |
| **D19S433** | 14 | 14 | * |
| **SE33** | 18/28.2 | 18/28.2 | * |
| **D22S1045** | 16/18 | 16/18 | * |
| **DYS391** | 10 | 10 | 10 |
| **FGA** | 21/26 | 21/26 | 21/26 |
| **DYS576** | 19 | 19 | * |
| **DYS570** | 17 | 17 | * |
| **Amelogenina** | XY  (male) | XY  (male) | XY  (male) |

PowerPlex Y23 System

| ***DNA locus* Y STR** | 2473  **Blood** | 2473  **Pre vasectomy semen** | 2473  **Post vasectomy seminal fluid** |
| --- | --- | --- | --- |
| **DYS576** | 19 | 19 |  |
| **DYS389I** | 13 | 13 |  |
| **DYS448** | 18 | 18 |  |
| **DYS389II** | 29 | 29 |  |
| **DYS19** | 14 | 14 |  |
| **DYS391** | 10 | 10 | 10 |
| **DYS481** | 20 | 20 | 20 |
| **DYS549** | 13 | 13 |  |
| **DYS533** | 12 | 12 |  |
| **DYS438** | 12 | 12 |  |
| **DYS437** | 15 | 15 |  |
| **DYS570** | 17 | 17 | 17 |
| **DYS635** | 23 | 23 | 20/23 |
| **DYS390** | 24 | 24 |  |
| **DYS439** | 12 | 12 |  |
| **DYS392** | 13 | 13 |  |
| **DYS643** | 10 | 10 |  |
| **DYS393** | 13 | 13 | 13 |
| **DYS458** | 17 | 17 | 17 |
| **DYS385** | 12/14 | 12/14 |  |
| **DYS456** | 15 | 15 |  |
| **Y_GATA_H4** | 12 | 12 |  |

sample 2475

PowerPlex Fusion 6C

| ***DNA locus*** | 2475  **Blood** | 2475  **Pre vasectomy semen** | 2475  **Post vasectomy seminal fluid** |
| --- | --- | --- | --- |
| **D3S1358** | 15/17 | 15/17 | 15/17 |
| **D1S1656** | 16 | 16 | 16 |
| **D2S441** | 11 | 11 | 11 |
| **D10S1248** | 12/13 | 12/13 | 13 |
| **D13S317** | 8/12 | 8/12 | 8 |
| **Penta E** | 10/16 | 10/16 | * |
| **D16S539** | 9/12 | 9/12 | 9/12 |
| **D18S51** | 12/13 | 12/13 | 12/13 |
| **D2S1338** | 18/19 | 18/19 | 18/19 |
| **CSF1PO** | 11/12 | 11/12 | 11 |
| **Penta D** | 2.2/16 | 2.2/16 | * |
| **TH01** | 7/9.3 | 7/9.3 | 7/9.3 |
| **vWA** | 16/20 | 16/20 | 16/20 |
| **D21S11** | 27/30 | 27/30 | 27/30 |
| **D7S820** | 9/12 | 9/12 | 9 |
| **D5S818** | 10/11 | 10/11 | * |
| **TPOX** | 8 | 8 | * |
| **D8S1179** | 10/13 | 10/13 | 10/13 |
| **D12S391** | 18/19 | 18/19 | 18/19 |
| **D19S433** | 11/14 | 11/14 | 11/14 |
| **SE33** | 16/30.2 | 16/30.2 | 16/30.2 |
| **D22S1045** | 15 | 15 | * |
| **DYS391** | 11 | 11 | 11 |
| **FGA** | 18/24 | 18/24 | 18/24 |
| **DYS576** | 18 | 18 | 18 |
| **DYS570** | 17 | 17 | * |
| **Amelogenina** | XY  (male) | XY  (male) | XY  (male) |

PowerPlex Y23 System

| ***DNA locus* Y STR** | 2475  **Blood** | 2475  **Pre vasectomy semen** | 2475  **Post vasectomy seminal fluid** |
| --- | --- | --- | --- |
| **DYS576** | * | 18 |  |
| **DYS389I** | * | 13 | 13 |
| **DYS448** | * | 19 |  |
| **DYS389II** | * | 29 |  |
| **DYS19** | * | 14 |  |
| **DYS391** | * | 11 | 11 |
| **DYS481** | * | 22 | 22 |
| **DYS549** | * | 13 |  |
| **DYS533** | * | 12 | 12 |
| **DYS438** | * | 12 |  |
| **DYS437** | * | 14 |  |
| **DYS570** | * | 17 | 17 |
| **DYS635** | * | 23 | 23 |
| **DYS390** | * | 25 | 24 |
| **DYS439** | * | 12 |  |
| **DYS392** | * | 13 |  |
| **DYS643** | * | 10 |  |
| **DYS393** | * | 13 | 13 |
| **DYS458** | * | 17 | 17 |
| **DYS385** | * | 11/14 |  |
| **DYS456** | * | 16 |  |
| **Y_GATA_H4** | * | 12 |  |

sample 2476

PowerPlex Fusion 6C

| ***DNA locus*** | 2476  **Blood** | 2476  **Pre vasectomy semen** | 2476  **Post vasectomy seminal fluid** |
| --- | --- | --- | --- |
| **D3S1358** | 15/17 | 15/17 | 15/17 |
| **D1S1656** | 15/16 | 15/16 | 15/16 |
| **D2S441** | 11 | 11 | 11 |
| **D10S1248** | 13/14 | 13/14 | 13 |
| **D13S317** | 8/12 | 8/12 | 12 |
| **Penta E** | 10/16 | 10/16 | * |
| **D16S539** | 10/12 | 10/12 | 10/12 |
| **D18S51** | 12/13 | 12/13 | 12/13 |
| **D2S1338** | 17/19 | 17/19 | * |
| **CSF1PO** | 10/11 | 10/11 | * |
| **Penta D** | 8/12 | 8/12 | * |
| **TH01** | 7/9.3 | 7/9.3 | 7/9.3 |
| **vWA** | 16/17 | 16/17 | 16/17 |
| **D21S11** | 28/29.1/32.2 ? | 28/32.2 | * |
| **D7S820** | 9/12 | 9/12 | * |
| **D5S818** | 10/11 | 10/11 | * |
| **TPOX** | 8 | 8 | * |
| **D8S1179** | 10/14 | 10/14 | 10/14 |
| **D12S391** | 15 | 15 | 15 |
| **D19S433** | 14/15.2 | 14/15.2 | 15.2 |
| **SE33** | 19/30.2 | 19/30.2 | * |
| **D22S1045** | 11 | 11 | * |
| **DYS391** | 11 | 11 | 11 |
| **FGA** | 13/23/26 ? | 23/26 | 23 |
| **DYS576** | 18 | 18 | * |
| **DYS570** | 17 | 17 | * |
| **Amelogenina** | XY  (male) | XY  (male) | XY  (male) |

PowerPlex Y23 System

| ***DNA locus* Y STR** | 2476  **Blood** | 2476  **Pre vasectomy semen** | 2476  **Post vasectomy seminal fluid** |
| --- | --- | --- | --- |
| **DYS576** | 18 | 18 |  |
| **DYS389I** | 13 | 13 |  |
| **DYS448** | 19 | 19 |  |
| **DYS389II** | 29 | 29 |  |
| **DYS19** | 14 | 14 |  |
| **DYS391** | 11 | 11 |  |
| **DYS481** | 22 | 22 |  |
| **DYS549** | 13 | 13 |  |
| **DYS533** | 12 | 12 |  |
| **DYS438** | 12 | 12 |  |
| **DYS437** | 14 | 14 |  |
| **DYS570** | 17 | 17 |  |
| **DYS635** | 23 | 23 |  |
| **DYS390** | 25 | 24 |  |
| **DYS439** | 12 | 12 |  |
| **DYS392** | 13 | 13 |  |
| **DYS643** | 10 | 10 |  |
| **DYS393** | 13 | 13 | 13 |
| **DYS458** | 17 | 17 | 17 |
| **DYS385** | 11/14 | 11/14 |  |
| **DYS456** | 16 | 16 |  |
| **Y_GATA_H4** | 12 | 12 |  |

sample 2477

PowerPlex Fusion 6C

| ***DNA locus*** | 2477  **Blood** | 2477  **Pre vasectomy semen** | 2477  **Post vasectomy seminal fluid** |
| --- | --- | --- | --- |
| **D3S1358** | 15/16 | 15/16 | 15/16 |
| **D1S1656** | 12/17.3 | 12/17.3 | 12/17.3 |
| **D2S441** | 11 | 11 | 11 |
| **D10S1248** | 11/13 | 11/13 | 11/13 |
| **D13S317** | 8/12 | 8/12 | 8/12 |
| **Penta E** | 11/12 | 11/12 | 11/12 |
| **D16S539** | 10/13 | 10/13 | 10/13 |
| **D18S51** | 16/18 | 16/18 | 16/18 |
| **D2S1338** | 16/24 | 16/24 | 16/24 |
| **CSF1PO** | 11/12 | 11/12 | 11/12 |
| **Penta D** | 9/13 | 9/13 | 9/13 |
| **TH01** | 7/9.3 | 7/9.3 | 7/9.3 |
| **vWA** | 16/16.1/17 ? | 17 | 17 |
| **D21S11** | 29 | 29 | 29 |
| **D7S820** | 8/10 | 8/10 | 8/10 |
| **D5S818** | 11/12 | 11/12 | 11/12 |
| **TPOX** | 8/11 | 8/11 | 8/11 |
| **D8S1179** | 12/15 | 12/15 | 12/15 |
| **D12S391** | 18/19 | 18/19 | 18/19 |
| **D19S433** | 14 | 13/14 | 13/14 |
| **SE33** | 23.2/27.2 | 23.2/27.2 | 23.2/27.2 |
| **D22S1045** | 15/16 | 15/16 | 16 |
| **DYS391** | 10 | 10 | 10 |
| **FGA** | 20/24 | 20/24 | 20/24 |
| **DYS576** | 16 | 16 | 16 |
| **DYS570** | 18 | 18 | 18 |
| **Amelogenina** | XY  (male) | XY  (male) | XY  (male) |

PowerPlex Y23 System

| ***DNA locus* Y STR** | 2477  **Blood** | 2477  **Pre vasectomy semen** | 2477  **Post vasectomy seminal fluid** |
| --- | --- | --- | --- |
| **DYS576** | 16 | 16 | 16 |
| **DYS389I** | 12 | 12 | 12 |
| **DYS448** | 20 | 20 | 20 |
| **DYS389II** | 28 | 28 | 28 |
| **DYS19** | 14 | 14 | 14 |
| **DYS391** | 10 | 10 | 10 |
| **DYS481** | 24 | 24 | 24 |
| **DYS549** | 12 | 12 | 12 |
| **DYS533** | 11 | 11 | 11 |
| **DYS438** | 10 | 10 | 10 |
| **DYS437** | 15 | 15 |  |
| **DYS570** | 18 | 18 | 18 |
| **DYS635** | 21 | 21 | 21 |
| **DYS390** | 23 | 23 | 23 |
| **DYS439** | 11 | 11 | 11 |
| **DYS392** | 11 | 11 | 11 |
| **DYS643** | 12 | 12 |  |
| **DYS393** | 13 | 13 | 13 |
| **DYS458** | 15 | 15 | 15 |
| **DYS385** | 13/15 | 13/15 | 13/15 |
| **DYS456** | 14 | 14 | 14 |
| **Y_GATA_H4** | 11 | 11 | 11 |

sample 2478

PowerPlex Fusion 6C

| ***DNA locus*** | 2478  **Blood** | 2478  **Pre vasectomy semen** | 2478  **Post vasectomy seminal fluid** |
| --- | --- | --- | --- |
| **D3S1358** | 16/17 | 16/17 | 16/17 |
| **D1S1656** | 16/16.3 | 16/16.3 | 16/16.3 |
| **D2S441** | 11.3/14 | 11.3/14 | 11.3/14 |
| **D10S1248** | 14 | 14 | 14 |
| **D13S317** | 9/11 | 9/11 | 9/11 |
| **Penta E** | 8/12 | 8/12 | 8 |
| **D16S539** | 10/11 | 10/11 | 10/11 |
| **D18S51** | 12/19 | 12/19 | 12/19 |
| **D2S1338** | 24 | 24 | 24 |
| **CSF1PO** | 10 | 10 | 10 |
| **Penta D** | 8/9 | 8/9 | 8/9 |
| **TH01** | 9/9.3 | 9/9.3 | 9/9.3 |
| **vWA** | 17 | 17 | 17 |
| **D21S11** | 27/29 | 27/29 | 27/29 |
| **D7S820** | 10/11 | 10/11 | 10/11 |
| **D5S818** | 12/13 | 12/13 | 12 |
| **TPOX** | 8/11 | 8 | * |
| **D8S1179** | 14 | 14 | 14 |
| **D12S391** | 18/23 | 18/23 | 18/23 |
| **D19S433** | 11/15 | 11/15 | 11/15 |
| **SE33** | 13/18 | 13/18 | 13/18 |
| **D22S1045** | 11/16 | 11/16 | * |
| **DYS391** | 10 | 10 | 10 |
| **FGA** | 22/24 | 22/24 | 22/24 |
| **DYS576** | 17 | 17 | 17 |
| **DYS570** | 19 | 19 | 19 |
| **Amelogenina** | XY  (male) | XY  (male) | XY  (male) |

PowerPlex Y23 System

| ***DNA locus* Y STR** | 2478  **Blood** | 2478  **Pre vasectomy semen** | 2478  **Post vasectomy seminal fluid** |
| --- | --- | --- | --- |
| **DYS576** | 17 | 17 | 17 |
| **DYS389I** | 14 | 14 | 14 |
| **DYS448** | 20 | 20 | 20 |
| **DYS389II** | 31 | 31 | 31 |
| **DYS19** | 15 | 15 | 15 |
| **DYS391** | 10 | 10 | 10 |
| **DYS481** | 28 | 28 | 28 |
| **DYS549** | 12 | 12 | 12 |
| **DYS533** | 11 | 11 | 11 |
| **DYS438** | 10 | 10 | 10 |
| **DYS437** | 14 | 14 | 14 |
| **DYS570** | 19 | 19 | 19 |
| **DYS635** | 21 | 21 | 21 |
| **DYS390** | 23 | 23 | 23 |
| **DYS439** | 12 | 12 | 12 |
| **DYS392** | 12 | 12 | 12 |
| **DYS643** | 12 | 12 | 12 |
| **DYS393** | 15 | 15 | 15 |
| **DYS458** | 15 | 15 | 15 |
| **DYS385** | 15 | 15 | 15 |
| **DYS456** | 14 | 14 | 14 |
| **Y_GATA_H4** | 10 | 10 | 10 |

sample 2480

PowerPlex Fusion 6C

| ***DNA locus*** | 2480  **Blood** | 2480  **Pre vasectomy semen** | 2480  **Post vasectomy seminal fluid** |
| --- | --- | --- | --- |
| **D3S1358** | 16/18 | 16/18 | 16/18 |
| **D1S1656** | 14/15 | 14/15 | 14/15 |
| **D2S441** | 10/11 | 10/11 | 10/11 |
| **D10S1248** | 11/14 | 11/14 | 11/14 |
| **D13S317** | 8/11 | 8/11 | 8/11 |
| **Penta E** | 7/12 | 7/12 | 7/12 |
| **D16S539** | 12 | 12 | 12 |
| **D18S51** | 12 | 12 | 12 |
| **D2S1338** | 21/25 | 21/25 | 21/25 |
| **CSF1PO** | 10/11 | 10/11 | 10/11 |
| **Penta D** | 11/13 | 11/13 | 11/13 |
| **TH01** | 6/9.3 | 6/9.3 | 6/9.3 |
| **vWA** | 16/17 | 16/17 | 16/17 |
| **D21S11** | 29/31.2 | 29/31.2 | 29/31.2 |
| **D7S820** | 8/10 | 8/10 | 8/10 |
| **D5S818** | 11/12 | 11/12 | 12 |
| **TPOX** | 8/10 | 8/10 | * |
| **D8S1179** | 10/12 | 10/12 | 10/12 |
| **D12S391** | 18/20 | 18/20 | 18/20 |
| **D19S433** | 14/15.2 | 14/15.2 | 14/15.2 |
| **SE33** | 18/25.2 | 18/25.2 | 18/25.2 |
| **D22S1045** | 11/15 | 11/15 | 11 |
| **DYS391** | 10 | 10 | 10 |
| **FGA** | 21/24 | 21/24 | 21/24 |
| **DYS576** | 17 | 17 | 17 |
| **DYS570** | 17 | 17 | * |
| **Amelogenina** | XY  (male) | XY  (male) | XY  (male) |

PowerPlex Y23 System

| ***DNA locus* Y STR** | 2480  **Blood** | 2480  **Pre vasectomy semen** | 2480  **Post vasectomy seminal fluid** |
| --- | --- | --- | --- |
| **DYS576** | 17 | 17 | 17 |
| **DYS389I** | 12 | 12 | 12 |
| **DYS448** | 20 | 20 | 20 |
| **DYS389II** | 28 | 28 | 28 |
| **DYS19** | 15 | 15 | 15 |
| **DYS391** | 10 | 10 | 10 |
| **DYS481** | 23 | 23 | 23 |
| **DYS549** | 13 | 13 | 13 |
| **DYS533** | 12 | 12 | 12 |
| **DYS438** | 9 | 9 | 9 |
| **DYS437** | 16 | 16 | 16 |
| **DYS570** | 17 | 17 | 17 |
| **DYS635** | 21 | 21 | 21 |
| **DYS390** | 23 | 23 | 23 |
| **DYS439** | 12 | 12 | 12 |
| **DYS392** | 11 | 11 | 11 |
| **DYS643** | 10 | 10 | 10 |
| **DYS393** | 12 | 12 | 12 |
| **DYS458** | 17 | 17 | 17 |
| **DYS385** | 14/17 | 14/17 | 14/17 |
| **DYS456** | 13 | 13 | 13 |
| **Y_GATA_H4** | 11 | 11 | 11 |

sample 2488

PowerPlex Fusion 6C

| ***DNA locus*** | 2488  **Blood** | 2488  **Pre vasectomy semen** | 2488  **Post vasectomy seminal fluid** |
| --- | --- | --- | --- |
| **D3S1358** | 15/18 | 15/18 | 15/18 |
| **D1S1656** | 11/17.3 | 11/17.3 | 11/17.3 |
| **D2S441** | 10/11.3 | 10/11.3 | 10/11.3 |
| **D10S1248** | 13 | 13 | 13 |
| **D13S317** | 12/13 | 12/13 | 12/13 |
| **Penta E** | 10/11 | 10/11 | 10/11 |
| **D16S539** | 11 | 11 | 11 |
| **D18S51** | 12/14 | 12/14 | 12/14 |
| **D2S1338** | 16/19 | 16/19 | 16/19 |
| **CSF1PO** | 10/12 | 10/12 | 10/12 |
| **Penta D** | 8/13 | 8/13 | 8/13 |
| **TH01** | 9/9.3 | 9/9.3 | 9/9.3 |
| **vWA** | 17/19 | 17/19 | 17/19 |
| **D21S11** | 29/30 | 29/30 | 29/30 |
| **D7S820** | 8/10 | 8/10 | 8/10 |
| **D5S818** | 7/12 | 7/12 | 7/12 |
| **TPOX** | 9/11 | 11 | 9/11 |
| **D8S1179** | 14/15 | 14/15 | 14/15 |
| **D12S391** | 22 | 22 | 22 |
| **D19S433** | 13 | 13 | 13 |
| **SE33** | 19/24.2 | 19/24.2 | 19/24.2 |
| **D22S1045** | 15 | 15 | 15 |
| **DYS391** | 10 | 10 | 10 |
| **FGA** | 9/21 | 19/21 | 19/21 |
| **DYS576** | 17 | 17 | 17 |
| **DYS570** | 18 | 18 | 18 |
| **Amelogenina** | XY  (male) | XY  (male) | XY  (male) |

PowerPlex Y23 System

| ***DNA locus* Y STR** | 2488  **Blood** | 2488  **Pre vasectomy semen** | 2488  **Post vasectomy seminal fluid** |
| --- | --- | --- | --- |
| **DYS576** | 17 | 17 | 17 |
| **DYS389I** | 13 | 13 | 13 |
| **DYS448** | 20 | 20 | 20 |
| **DYS389II** | 30 | 30 | 30 |
| **DYS19** | 13 | 13 | 13 |
| **DYS391** | 10 | 10 | 10 |
| **DYS481** | 22 | 22 | 22 |
| **DYS549** | 13 | 13 | 13 |
| **DYS533** | 12 | 12 | 12 |
| **DYS438** | 10 | 10 | 10 |
| **DYS437** | 14 |  | 14 |
| **DYS570** | 18 | 18 | 18 |
| **DYS635** | 21 | 21 | 21 |
| **DYS390** | 23 | 23 | 23 |
| **DYS439** | 12 | 11 | 12 |
| **DYS392** | 11 | 11 | 11 |
| **DYS643** | 12 | 12 | 12 |
| **DYS393** | 13 | 13 | 13 |
| **DYS458** | 15 | 15 | 15 |
| **DYS385** | 16/18 | 16/18 | 16/18 |
| **DYS456** | 16 | 16 | 16 |
| **Y_GATA_H4** | 12 | 12 | 12 |

sample 2489

PowerPlex Fusion 6C

| ***DNA locus*** | 2489  **Blood** | 2489  **Pre vasectomy semen** | 2489  **Post vasectomy seminal fluid** |
| --- | --- | --- | --- |
| **D3S1358** | 16/18 | 16/18 | 16/18 |
| **D1S1656** | 13/15 | 13/15 | 13/15 |
| **D2S441** | 12/14 | 12/14 | 12/14 |
| **D10S1248** | 13/14 | 13/14 | 13/14 |
| **D13S317** | 12 | 12 | 12 |
| **Penta E** | 12 | 12 | * |
| **D16S539** | 11/12 | 11/12 | 11/12 |
| **D18S51** | 13/15 | 13/15 | 13/15 |
| **D2S1338** | 22/23 | 22/23 | 22/23 |
| **CSF1PO** | 10/11 | 10/11 | 10 |
| **Penta D** | 9/11 | 9/11 | 9 |
| **TH01** | 7/9.3 | 7/9.3 | 7/9.3 |
| **vWA** | 16 | 16 | 16 |
| **D21S11** | 29/31.2 | 29/31.2 | 29/31.2 |
| **D7S820** | 11 | 11 | 11 |
| **D5S818** | 12 | 12 | 12 |
| **TPOX** | 8/12 | 8/12 | 8 |
| **D8S1179** | 12/15 | 12/15 | 12/15 |
| **D12S391** | 19/19.3 | 19/19.3 | 19/19.3 |
| **D19S433** | 10/14 | 10/14 | 10/14 |
| **SE33** | 15/18 | 15/18 | 15/18 |
| **D22S1045** | 16 | 16 | * |
| **DYS391** | 11 | 11 | 11 |
| **FGA** | 21/22 | 21/22 | 21/22 |
| **DYS576** | 17 | 17 | 17 |
| **DYS570** | 18 | 18 | 18 |
| **Amelogenina** | XY  (male) | XY  (male) | XY  (male) |

PowerPlex Y23 System

| ***DNA locus* Y STR** | 2489  **Blood** | 2489  **Pre vasectomy semen** | 2489  **Post vasectomy seminal fluid** |
| --- | --- | --- | --- |
| **DYS576** | 17 | 17 | 17 |
| **DYS389I** | 13 | 13 | 13 |
| **DYS448** | 17 | 17 | 17 |
| **DYS389II** | 29 | 29 | 29 |
| **DYS19** | 15 | 15 | 15 |
| **DYS391** | 11 | 11 | 11 |
| **DYS481** | 22 | 22 | 22 |
| **DYS549** | 12 | 12 | 12 |
| **DYS533** | 12 | 12 | 12 |
| **DYS438** | 12 | 12 | 12 |
| **DYS437** | 14 | 14 |  |
| **DYS570** | 18 | 18 | 18 |
| **DYS635** | 23 | 23 | 23 |
| **DYS390** | 25 | 25 | 25 |
| **DYS439** | 11 | 11 |  |
| **DYS392** | 13 | 13 |  |
| **DYS643** | 10 | 10 | OL |
| **DYS393** | 13 | 13 | 13 |
| **DYS458** | 17 | 17 | 17 |
| **DYS385** | 11/13 | 11/13 |  |
| **DYS456** | 17 | 17 | 17 |
| **Y_GATA_H4** | 11 | 11 |  |

sample 2490

PowerPlex Fusion 6C

| ***DNA locus*** | 2490  **Blood** | 2490  **Pre vasectomy semen** | 2490  **Post vasectomy seminal fluid** |
| --- | --- | --- | --- |
| **D3S1358** | 15/17 | 15/17 | 15/17 |
| **D1S1656** | 16.3/19.3 | 16.3/19.3 | 16.3/19.3 |
| **D2S441** | 12/12.3 | 12/12.3 | 12/12.3 |
| **D10S1248** | 15/16 | 15/16 | 15/16 |
| **D13S317** | 11/12 | 11/12 | 11/12 |
| **Penta E** | 15 | 15 | 15 |
| **D16S539** | 12/13 | 12/13 | 12/13 |
| **D18S51** | 16/19 | 16/19 | 16/19 |
| **D2S1338** | 19/25 | 19/25 | 19/25 |
| **CSF1PO** | 10/11 | 10/11 | 10/11 |
| **Penta D** | 9/14 | 9/14 | 9/14 |
| **TH01** | 7/8 | 7/8 | 7/8 |
| **vWA** | 17 | 17 | 17 |
| **D21S11** | 29/32.2 | 29/32.2 | 29/32.2 |
| **D7S820** | 11 | 11 | 11 |
| **D5S818** | 12 | 12 | 12 |
| **TPOX** | 8/9 | 8/9 | 8 |
| **D8S1179** | 10/14 | 10/14 | 10/14 |
| **D12S391** | 19 | 19 | 19 |
| **D19S433** | 13/14.2 | 13/14.2 | 13/14.2 |
| **SE33** | 15/28.2 | 15/28.2 | 15/28.2 |
| **D22S1045** | 16/17 | 16/17 | 17 |
| **DYS391** | 12 | 12 | 12 |
| **FGA** | 22 | 22 | 22 |
| **DYS576** | 17 | 17 | 17 |
| **DYS570** | 16 | 16 | 16 |
| **Amelogenina** | XY  (male) | XY  (male) | XY  (male) |

PowerPlex Y23 System

| ***DNA locus* Y STR** | 2490  **Blood** | 2490  **Pre vasectomy semen** | 2490  **Post vasectomy seminal fluid** |
| --- | --- | --- | --- |
| **DYS576** | 17 | 17 | 17 |
| **DYS389I** | 14 | 14 | 14 |
| **DYS448** | 18 | 18 | 18 |
| **DYS389II** | 30 | 30 | 30 |
| **DYS19** | 14 | 14 | 14 |
| **DYS391** | 12 | 12 | 12 |
| **DYS481** | 23 | 23 | 23 |
| **DYS549** | 13 | 13 | 13 |
| **DYS533** | 12 | 12 | 12 |
| **DYS438** | 12 | 12 | 12 |
| **DYS437** | 14 | 14 | 14 |
| **DYS570** | 16 | 16 | 16 |
| **DYS635** | 24 | 24 | 24 |
| **DYS390** | 23 | 23 | 23 |
| **DYS439** | 12 | 12 | 12 |
| **DYS392** | 13 | 13 | 13 |
| **DYS643** | 10 | 10 | 10 |
| **DYS393** | 13 | 13 | 13 |
| **DYS458** | 16 | 16 | 16 |
| **DYS385** | 10/14 | 10/14 | 10/14 |
| **DYS456** | 16 | 16 | 16 |
| **Y_GATA_H4** | 12 | 12 | 12 |

sample 2491

PowerPlex Fusion 6C

| ***DNA locus*** | 2491  **Blood** | 2491  **Pre vasectomy semen** | 2491  **Post vasectomy seminal fluid** |
| --- | --- | --- | --- |
| **D3S1358** | 15 | 15 | 15 |
| **D1S1656** | 13 | 13 | 13 |
| **D2S441** | 12.3/13 | 12.3/13 | 12.3/13 |
| **D10S1248** | 13/16 | 13/16 | 13 |
| **D13S317** | 12 | 12 | * |
| **Penta E** | 5/12 | 5/12 | * |
| **D16S539** | 11/12 | 11/12 | 11/12 |
| **D18S51** | 14/15 | 14/15 | 14/15 |
| **D2S1338** | 21/23 | 21/23 | * |
| **CSF1PO** | 11/12 | 11/12 | * |
| **Penta D** | 12/13 | 12/13 | * |
| **TH01** | 9/9.3 | 9/9.3 | 9/9.3 |
| **vWA** | 15/18 | 15/18 | 18 |
| **D21S11** | 29/31.2 | 29/31.2 | 29 |
| **D7S820** | 9/12 | 9/12 | * |
| **D5S818** | 10/12 | 10/12 | * |
| **TPOX** | 8 | 8 | * |
| **D8S1179** | 12/13 | 12/13 | 12/13 |
| **D12S391** | 17/18 | 17/18 | * |
| **D19S433** | 12/13 | 12/13 | 12 |
| **SE33** | 20/22.2 | 20/22.2 | * |
| **D22S1045** | 16/17 | 17 | * |
| **DYS391** | 11 | 11 | * |
| **FGA** | 19/24 | 19/24 | 19/24 |
| **DYS576** | 18 | 18 | * |
| **DYS570** | 17 | 17 | * |
| **Amelogenina** | XY  (male) | XY  (male) | XY  (male) |

PowerPlex Y23 System

| ***DNA locus* Y STR** | 2491  **Blood** | 2491  **Pre vasectomy semen** | 2491  **Post vasectomy seminal fluid** |
| --- | --- | --- | --- |
| **DYS576** | 18 | 18 |  |
| **DYS389I** | 13 | 13 | 13 |
| **DYS448** | 19 | 19 |  |
| **DYS389II** | 29 | 29 |  |
| **DYS19** | 14 | 14 |  |
| **DYS391** | 11 | 11 |  |
| **DYS481** | 22 | 22 |  |
| **DYS549** | 13 | 13 |  |
| **DYS533** | 13 | 13 |  |
| **DYS438** | 12 | 12 |  |
| **DYS437** | 15 | 15 |  |
| **DYS570** | 17 | 17 |  |
| **DYS635** | 23 | 23 | 23 |
| **DYS390** | 24 | 24 |  |
| **DYS439** | 12 | 12 |  |
| **DYS392** | 13 | 13 |  |
| **DYS643** | 10 | 10 |  |
| **DYS393** | 13 | 13 | 13 |
| **DYS458** | 16 | 16 | 16 |
| **DYS385** | 12/13 | 12/13 |  |
| **DYS456** | 16 | 16 |  |
| **Y_GATA_H4** | 12 | 12 |  |

sample 2492

PowerPlex Fusion 6C

| ***DNA locus*** | 2492  **Blood** | 2492  **Pre vasectomy semen** | 2492  **Post vasectomy seminal fluid** |
| --- | --- | --- | --- |
| **D3S1358** | 16 | 16 | 16 |
| **D1S1656** | 15/18.3 | 15/18.3 | 15/18.3 |
| **D2S441** | 11/11.3 | 11/11.3 | 11/11.3 |
| **D10S1248** | 13/14 | 13/14 | 13/14 |
| **D13S317** | 9/13 | 9/13 | 9/13 |
| **Penta E** | 12/15 | 12/15 | * |
| **D16S539** | 11/12 | 11/12 | 11/12 |
| **D18S51** | 11/19 | 11/19 | 11/19 |
| **D2S1338** | 16/23 | 16/23 | 16/23 |
| **CSF1PO** | 11 | 11 | 11 |
| **Penta D** | 10/11 | 10/11 | 10/11 |
| **TH01** | 8/9.3 | 8/9.3 | 8/9.3 |
| **vWA** | 16/17 | 16/17 | 16/17 |
| **D21S11** | 30/31.2 | 30/31.2 | 30/31.2 |
| **D7S820** | 9/10 | 9/10 | 9/10 |
| **D5S818** | 12/13 | 12/13 | 12/13 |
| **TPOX** | 11 | 11 | * |
| **D8S1179** | 11/13 | 11/13 | 11/13 |
| **D12S391** | 19/21 | 19/21 | 19/21 |
| **D19S433** | 11/14 | 11/14 | 11/14 |
| **SE33** | 18/22.2 | 18/22.2 | 18/22.2 |
| **D22S1045** | 15 | 15 | 15 |
| **DYS391** | 11 | 11 | 11 |
| **FGA** | 20/23 | 20/23 | 20/23 |
| **DYS576** | 17 | 17 | 17 |
| **DYS570** | 18 | 18 | * |
| **Amelogenina** | XY  (male) | XY  (male) | XY  (male) |

PowerPlex Y23 System

| ***DNA locus* Y STR** | 2492  **Blood** | 2492  **Pre vasectomy semen** | 2492  **Post vasectomy seminal fluid** |
| --- | --- | --- | --- |
| **DYS576** | 17 | 17 |  |
| **DYS389I** | 13 | 13 | 13 |
| **DYS448** | 19 | 19 | 19 |
| **DYS389II** | 30 | 30 | 30 |
| **DYS19** | 14 | 14 | 14 |
| **DYS391** | 11 | 11 | 11 |
| **DYS481** | 22 | 22 | 22 |
| **DYS549** | 13 | 13 |  |
| **DYS533** | 12 | 12 | 12 |
| **DYS438** | 12 | 12 | 12 |
| **DYS437** | 15 | 15 |  |
| **DYS570** | 18 | 18 | 18 |
| **DYS635** | 23 | 23 | 23 |
| **DYS390** | 23 | 23 | 23 |
| **DYS439** | 12 | 12 |  |
| **DYS392** | 13 | 13 |  |
| **DYS643** | 10 | 10 |  |
| **DYS393** | 13 | 13 | 13 |
| **DYS458** | 18 | 18 | 18 |
| **DYS385** | 11/14 | 11/14 |  |
| **DYS456** | 16 | 16 | 16 |
| **Y_GATA_H4** | 11 | 11 |  |

sample 2496

PowerPlex Fusion 6C

| ***DNA locus*** | 2496  **Blood** | 2496  **Pre vasectomy semen** | 2496  **Post vasectomy seminal fluid** |
| --- | --- | --- | --- |
| **D3S1358** | 17 | 17 | 17 |
| **D1S1656** | 12/15 | 12/15 | 12/15 |
| **D2S441** | 10 | 10 | 10 |
| **D10S1248** | 13/15 | 13/15 | * |
| **D13S317** | 11/12 | 11/12 | * |
| **Penta E** | 12/13 | 13 | * |
| **D16S539** | 10/11 | 10/11 | 10/11 |
| **D18S51** | 13/19 | 13/19 | 13/19 |
| **D2S1338** | 19/23 | 19/23 | 23 |
| **CSF1PO** | 10/13 | 10/13 | * |
| **Penta D** | 10/13 | 10/13 | * |
| **TH01** | 6 | 6 | 6 |
| **vWA** | 17/19 | 17/19 | 17/19 |
| **D21S11** | 31/31.2 | 31/31.2 | * |
| **D7S820** | 10/11 | 10/11 | 10 |
| **D5S818** | 11 | 11 | * |
| **TPOX** | 8/11 | 8/11 | * |
| **D8S1179** | 13 | 13 | 13 |
| **D12S391** | 17/18 | 17/18 | 17/18 |
| **D19S433** | 14/15 | 14/15 | 14/15 |
| **SE33** | 16/22.2 | 16/22.2 | 16 |
| **D22S1045** | OL | 15/16 | * |
| **DYS391** | 10 | 10 | 10 |
| **FGA** | 23/25 | 23/25 | 23/25 |
| **DYS576** | 19 | 19 | * |
| **DYS570** | 16 | 16 | * |
| **Amelogenina** | XY  (male) | XY  (male) | XY  (male) |

PowerPlex Y23 System

| ***DNA locus* Y STR** | 2496  **Blood** | 2496  **Pre vasectomy semen** | 2496  **Post vasectomy seminal fluid** |
| --- | --- | --- | --- |
| **DYS576** | 19 | 19 | 19 |
| **DYS389I** | 13 | 13 | 13 |
| **DYS448** | 20 | 20 | 20 |
| **DYS389II** | 29 | 29 | 29 |
| **DYS19** | 14 | 14 |  |
| **DYS391** | 10 | 10 | 10 |
| **DYS481** | 23 | 23 | 23 |
| **DYS549** | 12 | 12 | 12 |
| **DYS533** | 12 | 12 | 12 |
| **DYS438** | 12 | 12 | 12 |
| **DYS437** | 15 | 15 |  |
| **DYS570** | 16 | 16 | 16 |
| **DYS635** | 23 | 23 | 23 |
| **DYS390** | 24 | 24 | 24 |
| **DYS439** | 12 | 12 | 12 |
| **DYS392** | 13 | 13 |  |
| **DYS643** | 11 | 11 |  |
| **DYS393** | 12 | 12 | 12 |
| **DYS458** | 17 | 17 | 17 |
| **DYS385** | 11/14 | 11/14 |  |
| **DYS456** | 15 | 15 | 15 |
| **Y_GATA_H4** | 12 | 12 | 12 |

sample 2498

PowerPlex Fusion 6C

| ***DNA locus*** | 2498  **Blood** | 2498  **Pre vasectomy semen** | 2498  **Post vasectomy seminal fluid** |
| --- | --- | --- | --- |
| **D3S1358** | 15/16 | 15/16 | 15/16 |
| **D1S1656** | 16/18.3 | 16/18.3 | 16/18.3 |
| **D2S441** | 14 | 14 | 14 |
| **D10S1248** | 13/15 | 13/15 | 15 |
| **D13S317** | 5/12 | 5/12 | 5/12 |
| **Penta E** | 11/13 | 11/13 | * |
| **D16S539** | 9/11 | 9/11 | 9/11 |
| **D18S51** | 12/20 | 12/20 | 12/20 |
| **D2S1338** | 24 | 24 | 24 |
| **CSF1PO** | 11 | 11 | 11 |
| **Penta D** | 11/13 | 11/13 | 11 |
| **TH01** | 7 | 7 | 7 |
| **vWA** | 16/18 | 16/18 | 16/18 |
| **D21S11** | 31.2/33.2 | 31.2/33.2 | 31.2/33.2 |
| **D7S820** | 8/12 | 8/12 | 8/12 |
| **D5S818** | 11 | 11 | 11 |
| **TPOX** | 8 | 8 | 8 |
| **D8S1179** | 13/15 | 13/15 | 13/15 |
| **D12S391** | 18/19 | 18/19 | 18/19 |
| **D19S433** | 12/14 | 12/14 | 12/14 |
| **SE33** | 16/22 | 16/22 | 16/22 |
| **D22S1045** | 15/16 | 15/16 | * |
| **DYS391** | 10 | 10 | 10 |
| **FGA** | 22/24 | 22/24 | 22/24 |
| **DYS576** | 20 | 20 | 20 |
| **DYS570** | 17 | 17 | * |
| **Amelogenina** | XY  (male) | XY  (male) | XY  (male) |

PowerPlex Y23 System

| ***DNA locus* Y STR** | 2498  **Blood** | 2498  **Pre vasectomy semen** | 2498  **Post vasectomy seminal fluid** |
| --- | --- | --- | --- |
| **DYS576** | 20 | 20 | 20 |
| **DYS389I** | 12 | 12 | 12 |
| **DYS448** | 19 | 19 | 19 |
| **DYS389II** | 27 | 27 | 27 |
| **DYS19** | 14 | 14 | 14 |
| **DYS391** | 10 | 10 | 10 |
| **DYS481** | 22 | 22 | 22 |
| **DYS549** | 14 | 14 | 14 |
| **DYS533** | 12 | 12 | 12 |
| **DYS438** | 12 | 12 |  |
| **DYS437** | 15 | 15 |  |
| **DYS570** | 17 | 17 | 17 |
| **DYS635** | 23 | 23 | 23 |
| **DYS390** | 25 | 25 | 25 |
| **DYS439** | 12 | 12 | 12 |
| **DYS392** | 13 | 13 | 13 |
| **DYS643** | 11 | 11 |  |
| **DYS393** | 14 | 14 | 14 |
| **DYS458** | 17 | 17 | 17 |
| **DYS385** | 10/15 | 10/15 | 10 |
| **DYS456** | 17 | 17 | 17 |
| **Y_GATA_H4** | 12 | 12 |  |

sample 2499

PowerPlex Fusion 6C

| ***DNA locus*** | 2499  **Blood** | 2499  **Pre vasectomy semen** | 2499  **Post vasectomy seminal fluid** |
| --- | --- | --- | --- |
| **D3S1358** | 15/16 | 15/16 | 15/16 |
| **D1S1656** | 17.3 | 17.3 | 17.3 |
| **D2S441** | 11/14 | 11/14 | 11/14 |
| **D10S1248** | 11/13 | 11/13 | 13 |
| **D13S317** | 11/14 | 11/14 | 11 |
| **Penta E** | 7/19 | 7/19 | * |
| **D16S539** | 9/12 | 9/12 | 9/12 |
| **D18S51** | 12/14 | 12/14 | 12/14 |
| **D2S1338** | 18 | 18 | 18 |
| **CSF1PO** | 10 | 10 | 10 |
| **Penta D** | 8/9 | 8/9 | * |
| **TH01** | 9/9.3 | 9/9.3 | 9/9.3 |
| **vWA** | 17/19 | 17/19 | 17/19 |
| **D21S11** | 29/31 | 29/31 | 29 |
| **D7S820** | 9/10 | 9/10 | 9/10 |
| **D5S818** | 11/12 | 11/12 | * |
| **TPOX** | 8/10 | 8/10 | * |
| **D8S1179** | 12/14 | 12/14 | 12/14 |
| **D12S391** | 20/21 | 20/21 | 20/21 |
| **D19S433** | 12/14 | 12/14 | 12/14 |
| **SE33** | 13/17 | 13/17 | 13/17 |
| **D22S1045** | 16/17 | 16/17 | * |
| **DYS391** | 12 | 12 | 12 |
| **FGA** | 21 | 21 | 21 |
| **DYS576** | 16 | 16 | 16 |
| **DYS570** | 17 | 17 | * |
| **Amelogenina** | XY  (male) | XY  (male) | XY  (male) |

PowerPlex Y23 System

| ***DNA locus* Y STR** | 2499  **Blood** | 2499  **Pre vasectomy semen** | 2499  **Post vasectomy seminal fluid** |
| --- | --- | --- | --- |
| **DYS576** | 16 | 16 | 16 |
| **DYS389I** | 13 | 13 | 13 |
| **DYS448** | 19 | 19 | 19 |
| **DYS389II** | 29 | 29 | 29 |
| **DYS19** | 14 | 14 | 14 |
| **DYS391** | 12 | 12 | 12 |
| **DYS481** | 23 | 23 | 23 |
| **DYS549** | 11 | 11 | 11 |
| **DYS533** | 12 | 12 | 12 |
| **DYS438** | 12 | 12 | 12 |
| **1DYS437** | 15 | 15 | 15 |
| **DYS570** | 17 | 17 | 17 |
| **DYS635** | 23 | 23 | 23 |
| **DYS390** | 24 | 24 | 24 |
| **DYS439** | 13 | 13 | 13 |
| **DYS392** | 13 | 13 | 13 |
| **DYS643** | 10 | 10 | 10 |
| **DYS393** | 13 | 13 | 13 |
| **DYS458** | 18 | 18 | 18 |
| **DYS385** | 11/15 | 11/15 | 11/15 |
| **DYS456** | 15 | 15 | 15 |
| **Y_GATA_H4** | 12 | 12 | 12 |

sample 2500

PowerPlex Fusion 6C

| ***DNA locus*** | 2500  **Blood** | 2500  **Pre vasectomy semen** | 2500  **Post vasectomy seminal fluid** |
| --- | --- | --- | --- |
| **D3S1358** | 16/18 | 16/18 | 16/18 |
| **D1S1656** | 15 | 15 | 15 |
| **D2S441** | 11/14 | 11/14 | 11/14 |
| **D10S1248** | 11/13 | 11/13 | 11 |
| **D13S317** | 11/12 | 11/12 | 11/12 |
| **Penta E** | 15 | 15 | * |
| **D16S539** | 12/13 | 12/13 | 12/13 |
| **D18S51** | 16/18 | 16/18 | 16/18 |
| **D2S1338** | 20/22 | 20/22 | 20 |
| **CSF1PO** | 10 | 10 | 10 |
| **Penta D** | 8/11 | 8/11 | * |
| **TH01** | 9/9.3 | 9/9.3 | 9/9.3 |
| **vWA** | 15/17 | 15/17 | 15/17 |
| **D21S11** | 29/31 | 29/31 | 29/31 |
| **D7S820** | 10 | 10 | 10 |
| **D5S818** | 13/14 | 13/14 | * |
| **TPOX** | 8/9 | 8/9 | * |
| **D8S1179** | 15/16 | 15/16 | 15/16 |
| **D12S391** | 18 | 18 | 18 |
| **D19S433** | 14/15 | 14/15 | 15 |
| **SE33** | 14/29.2 | 14/29.2 | 14 |
| **D22S1045** | 16/17 | 16/17 | * |
| **DYS391** | 10 | 10 | 10 |
| **FGA** | 20 | 20 | 20 |
| **DYS576** | 17 | 17 | 17 |
| **DYS570** | 18 | 18 | 18 |
| **Amelogenina** | XY  (male) | XY  (male) | XY  (male) |

PowerPlex Y23 System

| ***DNA locus* Y STR** | 2500  **Blood** | 2500  **Pre vasectomy semen** | 2500  **Post vasectomy seminal fluid** |
| --- | --- | --- | --- |
| **DYS576** | 17 | 17 | 17 |
| **DYS389I** | 14 | 14 | 14 |
| **DYS448** | 21 | 21 | 21 |
| **DYS389II** | 31 | 31 | 31 |
| **DYS19** | 15 | 15 | 15 |
| **DYS391** | 10 | 10 | 10 |
| **DYS481** | 21 | 21 | 21 |
| **DYS549** | 12 | 12 | 12 |
| **DYS533** | 11 | 11 | 11 |
| **DYS438** | 10 | 10 | 10 |
| **DYS437** | 16 | 16 | 16 |
| **DYS570** | 18 | 18 | 18 |
| **DYS635** | 21 | 21 | 21 |
| **DYS390** | 22 | 22 | 22 |
| **DYS439** | 12 | 12 | 12 |
| **DYS392** | 11 | 11 | 11 |
| **DYS643** | 11 | 11 |  |
| **DYS393** | 14 | 14 | 14 |
| **DYS458** | 19 | 19 | 19 |
| **DYS385** | 13 | 13 | 13 |
| **DYS456** | 13 | 13 | 13 |
| **Y_GATA_H4** | 11 | 11 | 11 |

sample 2501

PowerPlex Fusion 6C

| ***DNA locus*** | 2501  **Blood** | 2501  **Pre vasectomy semen** | 2501  **Post vasectomy seminal fluid** |
| --- | --- | --- | --- |
| **D3S1358** | 15/16 | 15/16 | 15/16 |
| **D1S1656** | 12 | 12 | 12 |
| **D2S441** | 11.3/14 | 11.3/14 | 11.3/14 |
| **D10S1248** | 14/16 | 14/16 | 14/16 |
| **D13S317** | 12/13 | 12/13 | 12/13 |
| **Penta E** | 10/14 | 10/14 | 10/14 |
| **D16S539** | 9/11 | 9/11 | 9/11 |
| **D18S51** | 13/16 | 13/16 | 13/16 |
| **D2S1338** | 16/22 | 16/22 | 16/22 |
| **CSF1PO** | 10/12 | 10/12 | 10/12 |
| **Penta D** | 9/13 | 9/13 | 9/13 |
| **TH01** | 7/9.3 | 7/9.3 | 7/9.3 |
| **vWA** | 18/19 | 18/19 | 18/19 |
| **D21S11** | 32.2 | 32.2 | 32.2 |
| **D7S820** | 11 | 11 | 11 |
| **D5S818** | 12 | 12 | 12 |
| **TPOX** | 8/11 | 8/11 | 8 |
| **D8S1179** | 10/12 | 10/12 | 10/12 |
| **D12S391** | 19/24 | 19/24 | 19/24 |
| **D19S433** | 12/14 | 12/14 | 12/14 |
| **SE33** | 20/28.2 | 20/28.2 | 20/28.2 |
| **D22S1045** | 11/15 | 11/15 | 11/15 |
| **DYS391** | 11 | 11 | 11 |
| **FGA** | 21/22 | 21/22 | 21/22 |
| **DYS576** | 19 | 19 | 19 |
| **DYS570** | 17 | 17 | 17 |
| **Amelogenina** | XY  (male) | XY  (male) | XY  (male) |

PowerPlex Y23 System

| ***DNA locus* Y STR** | 2501  **Blood** | 2501  **Pre vasectomy semen** | 2501  **Post vasectomy seminal fluid** |
| --- | --- | --- | --- |
| **DYS576** | 19 | 19 | 19 |
| **DYS389I** | 12 | 12 | 12 |
| **DYS448** | 19 | 19 | 19 |
| **DYS389II** | 28 | 28 | 28 |
| **DYS19** | 15 | 15 | 15 |
| **DYS391** | 11 | 11 | 11 |
| **DYS481** | 22 | 22 | 22 |
| **DYS549** | 13 | 13 | 13 |
| **DYS533** | 12 | 12 | 12 |
| **DYS438** | 12 | 12 | 12 |
| **DYS437** | 15 | 15 | 15 |
| **DYS570** | 17 | 17 | 17 |
| **DYS635** | 23 | 23 | 23 |
| **DYS390** | 25 | 25 | 25 |
| **DYS439** | 12 | 12 | 12 |
| **DYS392** | 13 | 13 | 13 |
| **DYS643** | 10 | 10 | 10 |
| **DYS393** | 12 | 12 | 12 |
| **DYS458** | 17 | 17 | 17 |
| **DYS385** | 11/14 | 11/14 | 11/14 |
| **DYS456** | 15 | 15 | 15 |
| **Y_GATA_H4** | 12 | 12 | 12 |

sample 2502

PowerPlex Fusion 6C

| ***DNA locus*** | 2502  **Blood** | 2502  **Pre vasectomy semen** | 2502  **Post vasectomy seminal fluid** |
| --- | --- | --- | --- |
| **D3S1358** | 15/17 | 15/17 | 15/17 |
| **D1S1656** | 14/15 | 14/15 | 14/15 |
| **D2S441** | 11 | 11 | 11 |
| **D10S1248** | 14/15 | 14/15 | 14/15 |
| **D13S317** | 10/11 | 10/11 | 10/11 |
| **Penta E** | 11/15 | 11/15 | 11/15 |
| **D16S539** | 9/12 | 9/12 | 9/12 |
| **D18S51** | 13/20 | 13/20 | 13/20 |
| **D2S1338** | 19/25 | 19/25 | 19/25 |
| **CSF1PO** | 11/12 | 11/12 | 11/12 |
| **Penta D** | 9/13 | 9/13 | 9/13 |
| **TH01** | 8/9 | 8/9 | 8/9 |
| **vWA** | 15/17 | 15/17 | 15/17 |
| **D21S11** | 27/28 | 27/28 | 27/28 |
| **D7S820** | 10 | 10 | 10 |
| **D5S818** | 11/12 | 11/12 | 11/12 |
| **TPOX** | 8 | 8 | 8 |
| **D8S1179** | 11/15 | 11/15 | 11/15 |
| **D12S391** | 18/19 | 18/19 | 18/19 |
| **D19S433** | 13/14 | 13/14 | 13/14 |
| **SE33** | 14/21 | 14/21 | 14/21 |
| **D22S1045** | 15/16 | 15/16 | 16 |
| **DYS391** | 11 | 11 | 11 |
| **FGA** | 21/22 | 21/22 | 21/22 |
| **DYS576** | 19 | 19 | 19 |
| **DYS570** | 18 | 18 | 18 |
| **Amelogenina** | XY  (male) | XY  (male) | XY  (male) |

PowerPlex Y23 System

| ***DNA locus* Y STR** | 2502  **Blood** | 2502  **Pre vasectomy semen** | 2502  **Post vasectomy seminal fluid** |
| --- | --- | --- | --- |
| **DYS576** | 19 | 19 | 19 |
| **DYS389I** | 13 | 13 | 13 |
| **DYS448** | 19 | 19 | 19 |
| **DYS389II** | 29 | 29 | 29 |
| **DYS19** | 14 | 14 | 14 |
| **DYS391** | 11 | 1 | 11 |
| **DYS481** | 22 | 22 | 22 |
| **DYS549** | 11 | 11 | 11 |
| **DYS533** | 11 | 11 | 11 |
| **DYS438** | 12 | 12 | 12 |
| **DYS437** | 15 | 15 | 15 |
| **DYS570** | 17 | 18 | 18 |
| **DYS635** | 23 | 23 | 23 |
| **DYS390** | 25 | 25 | 25 |
| **DYS439** | 13 | 13 | 13 |
| **DYS392** | 13 | 13 | 13 |
| **DYS643** | 10 | 10 | 10 |
| **DYS393** | 11 | 11 | 11 |
| **DYS458** | 16 | 16 | 16 |
| **DYS385** | 11/15 | 11/15 | 11/15 |
| **DYS456** | 15 | 15 | 15 |
| **Y_GATA_H4** | 12 | 12 | 12 |

sample 2503

PowerPlex Fusion 6C

| ***DNA locus*** | 2503  **Blood** | 2503  **Pre vasectomy semen** | 2503  **Post vasectomy seminal fluid** |
| --- | --- | --- | --- |
| **D3S1358** | 14/17 | 14/17 | 14/17 |
| **D1S1656** | 15/15.3 | 15/15.3 | 15/15.3 |
| **D2S441** | 11 | 11 | 11 |
| **D10S1248** | 13/15 | 13/15 | 13/15 |
| **D13S317** | 8/12 | 8/12 | 8/12 |
| **Penta E** | 7/14 | 7/14 | 7/14 |
| **D16S539** | 12 | 12 | 12 |
| **D18S51** | 13/15 | 13/15 | 13/15 |
| **D2S1338** | 19/23 | 19/23 | 19/23 |
| **CSF1PO** | 11/13 | 11/13 | 11/13 |
| **Penta D** | 9/13 | 9/13 | 9/13 |
| **TH01** | 9/9.3 | 9/9.3 | 9/9.3 |
| **vWA** | 14/16 | 14/16 | 14/16 |
| **D21S11** | 30/31.2 | 30/31.2 | 30/31.2 |
| **D7S820** | 10/11 | 10/11 | 10/11 |
| **D5S818** | 12 | 12 | 12 |
| **TPOX** | 10/11 | * | 10/11 |
| **D8S1179** | 13/15 | 13/15 | 13/15 |
| **D12S391** | 17/19 | 17/19 | 17/19 |
| **D19S433** | 13.2/14.2 | 13.2/14.2 | 13.2/14.2 |
| **SE33** | 16/18 | 16/18 | 16/18 |
| **D22S1045** | 16 | 16 | 16 |
| **DYS391** | 11 | 11 | 11 |
| **FGA** | 23/24.2 | 23/24.2 | 23/24.2 |
| **DYS576** | 18 | 18 | 18 |
| **DYS570** | 18 | 18 | 18 |
| **Amelogenina** | XY  (male) | XY  (male) | XY  (male) |

PowerPlex Y23 System

| ***DNA locus* Y STR** | 2503  **Blood** | 2503  **Pre vasectomy semen** | 2503  **Post vasectomy seminal fluid** |
| --- | --- | --- | --- |
| **DYS576** | 18 | 18 | 18 |
| **DYS389I** | 13 | 13 | 13 |
| **DYS448** | 19 | 19 | 19 |
| **DYS389II** | 29 | 29 | 29 |
| **DYS19** | 14 | 14 | 14 |
| **DYS391** | 11 | 11 | 11 |
| **DYS481** | 23 | 23 | 23 |
| **DYS549** | 12 | 12 | 12 |
| **DYS533** | 13 | 13 | 13 |
| **DYS438** | 12 | 12 | 12 |
| **DYS437** | 15 | 15 |  |
| **DYS570** | 18 | 18 | 18 |
| **DYS635** | 23 | 23 | 23 |
| **DYS390** | 24 | 24 | 24 |
| **DYS439** | 12 | 12 | 12 |
| **DYS392** | 13 | 13 | 13 |
| **DYS643** | 10 | 10 | 10 |
| **DYS393** | 13 | 13 | 13 |
| **DYS458** | 18 | 18 | 18 |
| **DYS385** | 12/14 | 12/14 | 12/14 |
| **DYS456** | 15 | 15 | 15 |
| **Y_GATA_H4** | 12 | 12 | 12 |

sample 2505

PowerPlex Fusion 6C

| ***DNA locus*** | 2505  **Blood** | 2505  **Pre vasectomy semen** | 2505  **Post vasectomy seminal fluid** |
| --- | --- | --- | --- |
| **D3S1358** | 14/16 | 14/16 | 14/16 |
| **D1S1656** | 15/16 | 15/16 | 15/16 |
| **D2S441** | 11/11.3 | 11/11.3 | 11/11.3 |
| **D10S1248** | 13/15 | 13/15 | 13/15 |
| **D13S317** | 11 | 11 | 11 |
| **Penta E** | 5/16 | 5/16 | 5 |
| **D16S539** | 12/13 | 12/13 | 12/13 |
| **D18S51** | 16/17 | 16/17 | 16/17 |
| **D2S1338** | 18/27 | 18/27 | 18/27 |
| **CSF1PO** | 10/11 | 10/11 | 10/11 |
| **Penta D** | 5/11 | 5/11 | 5/11 |
| **TH01** | 6/9.3 | 6/9.3 | 6/9.3 |
| **vWA** | 15/16 | 15/16 | 15/16 |
| **D21S11** | 30/33.2 | 30/33.2 | 30/33.2 |
| **D7S820** | 10 | 10 | 10 |
| **D5S818** | 12 | 11/12 | 12 |
| **TPOX** | 9/12 | 9/12 | * |
| **D8S1179** | 13 | 13 | 13 |
| **D12S391** | 18 | 18 | 18 |
| **D19S433** | 13/14.2 | 13/14.2 | 13/14.2 |
| **SE33** | 14/OL | 14/OL | 14/OL |
| **D22S1045** | 11/15 | 11/15 | * |
| **DYS391** | 10 | 10 | 10 |
| **FGA** | 23/25 | 23/25 | 23/25 |
| **DYS576** | 17 | 17 | 17 |
| **DYS570** | 19 | 19 | 19 |
| **Amelogenina** | XY  (male) | XY  (male) | XY  (male) |

PowerPlex Y23 System

| ***DNA locus* Y STR** | 2505  **Blood** | 2505  **Pre vasectomy semen** | 2505  **Post vasectomy seminal fluid** |
| --- | --- | --- | --- |
| **DYS576** | 17 | 17 | 17 |
| **DYS389I** | 13 | 13 | 13 |
| **DYS448** | 21 | 21 | 21 |
| **DYS389II** | 29 | 29 | 29 |
| **DYS19** | 14 | 14 | 14 |
| **DYS391** | 10 | 10 | 10 |
| **DYS481** | 23 | 23 | 23 |
| **DYS549** | 13 | 13 | 13 |
| **DYS533** | 11 | 11 | 11 |
| **DYS438** | 9 | 9 | 9 |
| **DYS437** | 15 | 15 | 15 |
| **DYS570** | OL | 19 | 19 |
| **DYS635** | 24 | 24 | 24 |
| **DYS390** | 24 | 24 | 24 |
| **DYS439** | 12 | 12 | 12 |
| **DYS392** | 11 | 11 | 11 |
| **DYS643** | 10 | 10 | 10 |
| **DYS393** | OL | 12 | 12 |
| **DYS458** | 17 | 17 | 17 |
| **DYS385** | 14/15 | 14/15 | 14/15 |
| **DYS456** | 17 | 17 | 17 |
| **Y_GATA_H4** | 12 | 12 | 12 |

sample 2512

PowerPlex Fusion 6C

| ***DNA locus*** | 2512  **Blood** | 2512  **Pre vasectomy semen** | 2512  **Post vasectomy seminal fluid** |
| --- | --- | --- | --- |
| **D3S1358** | 17/18 | 17/18 | 17/18 |
| **D1S1656** | 13/16.3 | 13/16.3 | 13/16.3 |
| **D2S441** | 10 | 10 | 10 |
| **D10S1248** | 14 | 14 | 14 |
| **D13S317** | 11/13 | 11/13 | 11/13 |
| **Penta E** | 13/15 | 13/15 | 13/15 |
| **D16S539** | 10/11 | 10/11 | 10/11 |
| **D18S51** | 14/15 | 14/15 | 14/15 |
| **D2S1338** | 19 | 19 | 19 |
| **CSF1PO** | 11/13 | 11/13 | 11/13 |
| **Penta D** | 9/14 | 9/14 | 9/14 |
| **TH01** | 9.3 | 9.3 | 9.3 |
| **vWA** | 16/18 | 16/18 | 16/18 |
| **D21S11** | 29/30.2 | 29/30.2 | 29/30.2 |
| **D7S820** | 10/11 | 10/11 | 10/11 |
| **D5S818** | 13 | 13 | 13 |
| **TPOX** | 9/10 | 9/10 | 9/10 |
| **D8S1179** | 12/13 | 12/13 | 12/13 |
| **D12S391** | 18/19 | 18/19 | 18/19 |
| **D19S433** | 12.2/13 | 12.2/13 | 12.2/13 |
| **SE33** | 28.2/29.2 | 28.2/29.2 | 28.2/29.2 |
| **D22S1045** | 13/16 | 13/16 | 13/16 |
| **DYS391** | 11 | 11 | 11 |
| **FGA** | 21/23 | 21/23 | 21/23 |
| **DYS576** | 19 | 19 | 19 |
| **DYS570** | 16 | 16 | 16 |
| **Amelogenina** | XY  (male) | XY  (male) | XY  (male) |

PowerPlex Y23 System

| ***DNA locus* Y STR** | 2512  **Blood** | 2512  **Pre vasectomy semen** | 2512  **Post vasectomy seminal fluid** |
| --- | --- | --- | --- |
| **DYS576** | 19 | 19 | 15 |
| **DYS389I** | 13 | 13 | 13 |
| **DYS448** | 19 | 19 |  |
| **DYS389II** | 28 | 28 |  |
| **DYS19** | 14 | 14 |  |
| **DYS391** | 11 | 11 | 11 |
| **DYS481** | 22 | 22 |  |
| **DYS549** | 13 | 13 |  |
| **DYS533** | 12 | 12 |  |
| **DYS438** | 12 | 12 |  |
| **DYS437** | 15 | 15 |  |
| **DYS570** | 16 | 16 | 14.2/16 |
| **DYS635** | 23 | 23 | 23 |
| **DYS390** | 24 | 24 |  |
| **DYS439** | 13 | 13 |  |
| **DYS392** | 13 | 13 |  |
| **DYS643** | 10 | 10 |  |
| **DYS393** | 13 | 13 | 13 |
| **DYS458** | 17 | 17 |  |
| **DYS385** | 12/15 | 12/15 |  |
| **DYS456** | 15 | 15 |  |
| **Y_GATA_H4** | 11 | 11 |  |

sample 2514

PowerPlex Fusion 6C

| ***DNA locus*** | 2514  **Blood** | 2514  **Pre vasectomy semen** | 2514  **Post vasectomy seminal fluid** |
| --- | --- | --- | --- |
| **D3S1358** | 15/18 | 15/18 | 15/18 |
| **D1S1656** | 13/15 | 13/15 | 13/15 |
| **D2S441** | 12/14 | 12/14 | 12/14 |
| **D10S1248** | 14/15 | 14/15 | 14/15 |
| **D13S317** | 10/11 | 10/11 | 10/11 |
| **Penta E** | 9/12 | 9/12 | 9/12 |
| **D16S539** | 11/12 | 11/12 | 11/12 |
| **D18S51** | 13/16 | 13/16 | 13/16 |
| **D2S1338** | 17/19 | 17/19 | 17/19 |
| **CSF1PO** | 11/13 | 11/13 | 11/13 |
| **Penta D** | 9/11 | 9/11 | 9/11 |
| **TH01** | 7/9.3 | 7/9.3 | 7/9.3 |
| **vWA** | 16/17 | 16/17 | 16/17 |
| **D21S11** | 30.1/31/32.1/32.2? | 31/32.2 | 31/32.2 |
| **D7S820** | 12 | 12 | 12 |
| **D5S818** | 10/11 ? | 10/11 | 10/11 |
| **TPOX** | 8/11 | 8/11 | 8/11 |
| **D8S1179** | 11/13 | 11/13 | 11/13 |
| **D12S391** | 17.3/18/18.3/22 ? | 18/22 | 18/22 |
| **D19S433** | 13/14/15/16.2 ? | 13/14 | 13/14 |
| **SE33** | 17/18/20/21 ? | 18/21 | 18/21 |
| **D22S1045** | 15/16/17 ? | 15/17 | 15/17 |
| **DYS391** | 10 | 10 | 10 |
| **FGA** | 24/25 ? | 24/25 | 24/25 |
| **DYS576** | 16/18/19 ? | 18 | 18 |
| **DYS570** | 17 | 17 | 17 |
| **Amelogenina** | XY  (male) | XY  (male) | XY  (male) |

PowerPlex Y23 System

| ***DNA locus* Y STR** | 2514  **Blood** | 2514  **Pre vasectomy semen** | 2514  **Post vasectomy seminal fluid** |
| --- | --- | --- | --- |
| **DYS576** | 18 | 18 | 18 |
| **DYS389I** | 13 | 13 | 13 |
| **DYS448** | 20 | 20 | 20 |
| **DYS389II** | 31 | 31 |  |
| **DYS19** | 13 | 13 |  |
| **DYS391** | 10 | 10 | 10 |
| **DYS481** | 25 | 25 | 25 |
| **DYS549** | 12 | 12 | 12 |
| **DYS533** | 10 | 10 | 10 |
| **DYS438** | 10 | 10 |  |
| **DYS437** | 14 | 14 |  |
| **DYS570** | 17 | 17 | 17 |
| **DYS635** | 24 | 24 | 24 |
| **DYS390** | 24 | 24 | 24 |
| **DYS439** | 12 | 12 |  |
| **DYS392** | 11 | 11 |  |
| **DYS643** | 12 | 12 |  |
| **DYS393** | 13 | 13 | 13 |
| **DYS458** | 19 | 19 | 19 |
| **DYS385** | 16/17 | 16/17 |  |
| **DYS456** | 15 | 15 |  |
| **Y_GATA_H4** | 11 | 11 |  |

sample 2515

PowerPlex Fusion 6C

| ***DNA locus*** | 2515  **Blood** | 2515  **Pre vasectomy semen** | 2515  **Post vasectomy seminal fluid** |
| --- | --- | --- | --- |
| **D3S1358** | 17 | 17 | 17 |
| **D1S1656** | 11/13 | 11/13 | 11/13 |
| **D2S441** | 11/11.3 | 11/11.3 | 11/11.3 |
| **D10S1248** | 14/16 | 14/16 | 14/16 |
| **D13S317** | 9/10 | 9/10 | 9/10 |
| **Penta E** | 7/14 | 7/14 | 7/14 |
| **D16S539** | 9/13 | 9/13 | 9/13 |
| **D18S51** | 12/13 | 12/13 | 12/13 |
| **D2S1338** | 17/25 | 17/25 | 17/25 |
| **CSF1PO** | 11/12 | 11/12 | 11/12 |
| **Penta D** | 12 | 12 | 12 |
| **TH01** | 9/9.3 | 9/9.3 | 9/9.3 |
| **vWA** | 16 | 16 | 16 |
| **D21S11** | 29/32.2 | 29/32.2 | 29/32.2 |
| **D7S820** | 8/13 | 8/13 | 8/13 |
| **D5S818** | 11/12 | 11/12 | 11 |
| **TPOX** | 8 | 8 | 8 |
| **D8S1179** | 12/14 | 12/14 | 12/14 |
| **D12S391** | 20 | 20 | 20 |
| **D19S433** | 13/14 | 13/14 | 13/14 |
| **SE33** | 13/18 | 13/18 | 13/18 |
| **D22S1045** | 15/16 | 15/16 | 15/16 |
| **DYS391** | 9 | 9 | 9 |
| **FGA** | 20/24 | 20/24 | 20/24 |
| **DYS576** | 17 | 17 | 17 |
| **DYS570** | 23 | 23 | 23 |
| **Amelogenina** | XY  (male) | XY  (male) | XY  (male) |

PowerPlex Y23 System

| ***DNA locus* Y STR** | 2515  **Blood** | 2515  **Pre vasectomy semen** | 2515  **Post vasectomy seminal fluid** |
| --- | --- | --- | --- |
| **DYS576** | 17 | 17 | 17 |
| **DYS389I** | 14 | 14 | 14 |
| **DYS448** | 20 | 20 | 20 |
| **DYS389II** | 30 | 30 | 30 |
| **DYS19** | 13 | 13 | 13 |
| **DYS391** | 9 | 9 | 9 |
| **DYS481** | 27 | 27 | 27 |
| **DYS549** | 11 | 11 | 11 |
| **DYS533** | 11 | 11 | 11 |
| **DYS438** | 10 | 10 | 10 |
| **DYS437** | 14 | 14 | 14 |
| **DYS570** | 23 | 23 | 23 |
| **DYS635** | 21 | 21 | 21 |
| **DYS390** | 24 | 24 | 24 |
| **DYS439** | 10 | 10 | 10 |
| **DYS392** | 11 | 11 | 11 |
| **DYS643** | 12 | 12 | 12 |
| **DYS393** | 13 | 13 | 13 |
| **DYS458** | 18 | 18 | 18 |
| **DYS385** | 13/14 | 13/14 | 13/14 |
| **DYS456** | 14 | 14 | 14 |
| **Y_GATA_H4** | 12 | 12 | 12 |

sample 2517

PowerPlex Fusion 6C

| ***DNA locus*** | 2517  **Blood** | 2517  **Pre vasectomy semen** | 2517  **Post vasectomy seminal fluid** |
| --- | --- | --- | --- |
| **D3S1358** | 16 | 16 | 16 |
| **D1S1656** | 16 | 16 | 16 |
| **D2S441** | 10/11 | 10/11 | 10/11 |
| **D10S1248** | 12/15 | 12/15 | 12 |
| **D13S317** | 9/11 | 9/11 | 9 |
| **Penta E** | 7/17 | 7/17 | 7 |
| **D16S539** | 9/13 | 9/13 | 9/13 |
| **D18S51** | 15/20 | 15/20 | 15/20 |
| **D2S1338** | 21/23 | 21/23 | 21/23 |
| **CSF1PO** | 10/11 | 10/11 | * |
| **Penta D** | 7/10 | 7/10 | * |
| **TH01** | 6/7 | 6/7 | 6/7 |
| **vWA** | 15/16 | 15/16 | 15/16 |
| **D21S11** | 29/30 | 29/30 | 29/30 |
| **D7S820** | 11/12 | 11/12 | 11/12 |
| **D5S818** | 10/11 | 10/11 | * |
| **TPOX** | 11 | 11 | * |
| **D8S1179** | 14/15 | 14/15 | 14/15 |
| **D12S391** | 18/18.3 | 18/18.3 | 18/18.3 |
| **D19S433** | 13.2/15 | 13.2/15 | 13.2/15 |
| **SE33** | 20/23.2 | 20/23.2 | 20/23.2 |
| **D22S1045** | 15/16 | 15/16 | * |
| **DYS391** | 10 | 10 | 10 |
| **FGA** | 23/27 | 23/27 | 23/27 |
| **DYS576** | 17 | 17 | * |
| **DYS570** | 17 | 17 | * |
| **Amelogenina** | XY  (male) | XY  (male) | XY  (male) |

PowerPlex Y23 System

| ***DNA locus* Y STR** | 2517  **Blood** | 2517  **Pre vasectomy semen** | 2517  **Post vasectomy seminal fluid** |
| --- | --- | --- | --- |
| **DYS576** | * | 17 | 17 |
| **DYS389I** | * | 13 | 13 |
| **DYS448** | * | 21 | 21 |
| **DYS389II** | * | 30 | 30 |
| **DYS19** | * | 15 | 15 |
| **DYS391** | * | 10 | 10 |
| **DYS481** | * | 27 | 27 |
| **DYS549** | * | 11 | 11 |
| **DYS533** | * | 11 | 11 |
| **DYS438** | * | 11 | 11 |
| **DYS437** | * | 14 |  |
| **DYS570** | * | 17 | 17 |
| **DYS635** | * | 21 | 21 |
| **DYS390** | * | 21 | 21 |
| **DYS439** | * | 12 | 12 |
| **DYS392** | * | 11 | 11 |
| **DYS643** | * | 13 | 13 |
| **DYS393** | * | 14 | 14 |
| **DYS458** | * | 17 | 17 |
| **DYS385** | * | 17/18 | 17/18 |
| **DYS456** | * | 17 | 17 |
| **Y_GATA_H4** | * | 11 | 11 |

sample 2518

PowerPlex Fusion 6C

| ***DNA locus*** | 2518  **Blood** | 2518  **Pre vasectomy semen** | 2518  **Post vasectomy seminal fluid** |
| --- | --- | --- | --- |
| **D3S1358** | 15 | 15 | 15 |
| **D1S1656** | 16/17.3 | 16/17.3 | 16/17.3 |
| **D2S441** | 10/11 | 10/11 | 10/11 |
| **D10S1248** | 15 | 15 | 15 |
| **D13S317** | 9/12 | 9/12 | 9 |
| **Penta E** | 12/17 | 12/17 | * |
| **D16S539** | 11 | 11 | 11 |
| **D18S51** | 15/20 | 15/20 | 15/20 |
| **D2S1338** | 17 | 17 | 17 |
| **CSF1PO** | 10/13 | 10/13 | 10/13 |
| **Penta D** | 9/15 | 9/15 | * |
| **TH01** | 6 | 6 | 6 |
| **vWA** | 16 | 16 | 16 |
| **D21S11** | 29/32.2 | 29/32.2 | 29/32.2 |
| **D7S820** | 10/12 | 10/12 | 10/12 |
| **D5S818** | 9/11 | 9/11 | 11 |
| **TPOX** | 8/11 | 8/11 | * |
| **D8S1179** | 12/15 | 12/15 | 12/15 |
| **D12S391** | 17.3/18/19 ? | 18/19 | 18/19 |
| **D19S433** | 13/15 | 13/15 | 13/15 |
| **SE33** | 17/27.2 | 17/27.2 | 17/27.2 |
| **D22S1045** | 11/15 | 11/15 | * |
| **DYS391** | 10 | 10 | 10 |
| **FGA** | 21/22 | 21/22 | 21/22 |
| **DYS576** | 17 | 17 | 17 |
| **DYS570** | 18 | 18 | * |
| **Amelogenina** | XY  (male) | XY  (male) | XY  (male) |

PowerPlex Y23 System

| ***DNA locus* Y STR** | 2518  **Blood** | 2518  **Pre vasectomy semen** | 2518  **Post vasectomy seminal fluid** |
| --- | --- | --- | --- |
| **DYS576** | 17 | 17 | 17 |
| **DYS389I** | 14 | 14 | 14 |
| **DYS448** | 21 | 21 | 21 |
| **DYS389II** | 31 | 31 | 31 |
| **DYS19** | 15 | 15 |  |
| **DYS391** | 10 | 10 | 10 |
| **DYS481** | 25 | 25 | 25 |
| **DYS549** | 11 | 11 | 11 |
| **DYS533** | 12 | 12 | 12 |
| **DYS438** | 10 | 10 | 10 |
| **DYS437** | 15 | 15 | 15 |
| **DYS570** | 18 | 18 | 18 |
| **DYS635** | 21 | 21 | 21 |
| **DYS390** | 23 | 23 | 23 |
| **DYS439** | 12 | 12 | 12 |
| **DYS392** | 12 | 12 | 12 |
| **DYS643** | 12 | 12 | 12 |
| **DYS393** | 14 | 14 | 14 |
| **DYS458** | 16 | 16 | 16 |
| **DYS385** | 16 | 16 | 16 |
| **DYS456** | 13 | 13 | 13 |
| **Y_GATA_H4** | 12 | 12 | 12 |

sample 2548

PowerPlex Fusion 6C

| ***DNA locus*** | 2548  **Blood** | 2548  **Pre vasectomy semen** | 2548  **Post vasectomy seminal fluid** |
| --- | --- | --- | --- |
| **D3S1358** | 15/17 | 15/17 | 15/17 |
| **D1S1656** | 16/17.3 | 16/17.3 | 16/17.3 |
| **D2S441** | 10 | 10 | 10 |
| **D10S1248** | 14/15 | 14/15 | 14/15 |
| **D13S317** | 11/12 | 11/12 | 11/12 |
| **Penta E** | 5/13 | 5/13 | 5/13 |
| **D16S539** | 9/14 | 9/14 | 9/14 |
| **D18S51** | 13/16 | 13/16 | 13/16 |
| **D2S1338** | 17/19 | 17/19 | 17/19 |
| **CSF1PO** | 10/12 | 10/12 | 10/12 |
| **Penta D** | 9/13 | 9/13 | 9/13 |
| **TH01** | 7/9.3 | 7/9.3 | 7/9.3 |
| **vWA** | 15/16 | 15/16 | 15/16 |
| **D21S11** | 28/32.2 | 28/32.2 | 28/32.2 |
| **D7S820** | 8/11 | 8/11 | 8/11 |
| **D5S818** | 10/11 | 10/11 | 10/11 |
| **TPOX** | 8 | 8 | 8 |
| **D8S1179** | 14/15 | 14/15 | 14/15 |
| **D12S391** | 20/OL ? | 20/OL | 20/OL |
| **D19S433** | 13/15 | 13/15 | 13/15 |
| **SE33** | 17/18 | 17/18 | 17/18 |
| **D22S1045** | 15/16 | 15/16 | 15 |
| **DYS391** | 10 | 10 | 10 |
| **FGA** | 22/24 | 22/24 | 22/24 |
| **DYS576** | 17 | 17 | 17 |
| **DYS570** | 17 | 17 | 17 |
| **Amelogenina** | XY  (male) | XY  (male) | XY  (male) |

PowerPlex Y23 System

| ***DNA locus* Y STR** | 2548  **Blood** | 2548  **Pre vasectomy semen** | 2548  **Post vasectomy seminal fluid** |
| --- | --- | --- | --- |
| **DYS576** | 17 | 17 | 17 |
| **DYS389I** | 13 | 13 | 13 |
| **DYS448** | 20 | 20 | 20 |
| **DYS389II** | 30 | 30 | 30 |
| **DYS19** | 16 | 16 | 16 |
| **DYS391** | 10 | 10 | 10 |
| **DYS481** | 22 | 22 | 22 |
| **DYS549** | 13 | 13 | 13 |
| **DYS533** | 12 | 12 | 12 |
| **DYS438** | 12 | 12 | 12 |
| **DYS437** | 14 | 14 | 14 |
| **DYS570** | 17 | 17 | 17 |
| **DYS635** | 23 | 23 | 23 |
| **DYS390** | 24 | 24 | 24 |
| **DYS439** | 10 | 10 | 10 |
| **DYS392** | 11 | 11 | 11 |
| **DYS643** | 10 | 10 | 10 |
| **DYS393** | 14 | 14 | 14 |
| **DYS458** | 16 | 16 | 16 |
| **DYS385** | 11/15 | 11/15 | 11/15 |
| **DYS456** | 18 | 18 | 18 |
| **Y_GATA_H4** | 11 | 11 | 11 |

sample 2550

PowerPlex Fusion 6C

| ***DNA locus*** | 2550  **Blood** | 2550  **Pre vasectomy semen** | 2550  **Post vasectomy seminal fluid** |
| --- | --- | --- | --- |
| **D3S1358** | 14/15 | 14/15 | 14/15 |
| **D1S1656** | 15/17.3 | 15/17.3 | 15/17.3 |
| **D2S441** | 11/14 | 11/14 | 11/14 |
| **D10S1248** | 14/15 | 14/15 | 14/15 |
| **D13S317** | 11/12 | 11/12 | 11/12 |
| **Penta E** | 8/15 | 8/15 | 8/15 |
| **D16S539** | 11/12 | 11/12 | 11/12 |
| **D18S51** | 15/17 | 15/17 | 15/17 |
| **D2S1338** | 17/20 | 17/20 | 17/20 |
| **CSF1PO** | 11/12 | 11/12 | 11/12 |
| **Penta D** | 9/12 | 9/12 | 12 |
| **TH01** | 6 | 6 | 6 |
| **vWA** | 17/18 | 17/18 | 17/18 |
| **D21S11** | 30 | 30 | 30 |
| **D7S820** | 8/9 | 8/9 | 8/9 |
| **D5S818** | 11/13 | 11/13 | 11 |
| **TPOX** | 7/11 | 7/11 | 7 |
| **D8S1179** | 13/14 | 13/14 | 13/14 |
| **D12S391** | 19/20 | 19/20 | 19/20 |
| **D19S433** | 14 | 14 | 14 |
| **SE33** | 26.2/30.2 | 26.2/30.2 | 26.2/30.2 |
| **D22S1045** | 15/16 | 15/16 | * |
| **DYS391** | 11 | 11 | 11 |
| **FGA** | 22 ? | 22 | 22 |
| **DYS576** | 18 | 18 | 18 |
| **DYS570** | 18 | 18 | 18 |
| **Amelogenina** | XY  (male) | XY  (male) | XY  (male) |

PowerPlex Y23 System

| ***DNA locus* Y STR** | 2550  **Blood** | 2550  **Pre vasectomy semen** | 2550  **Post vasectomy seminal fluid** |
| --- | --- | --- | --- |
| **DYS576** | 18 | 18 | 18 |
| **DYS389I** | 14 | 14 | 14 |
| **DYS448** | 18 | 18 | 18 |
| **DYS389II** | 31 | 31 | 31 |
| **DYS19** | 14 | 14 | 14 |
| **DYS391** | 11 | 11 | 11 |
| **DYS481** | 22 | 22 | 22 |
| **DYS549** | 13 | 13 | 13 |
| **DYS533** | 12 | 12 | 12 |
| **DYS438** | 12 | 12 | 12 |
| **DYS437** | 14 | 14 |  |
| **DYS570** | 18 | 18 | 18 |
| **DYS635** | 23 | 23 | 23 |
| **DYS390** | 24 | 24 | 24 |
| **DYS439** | 12 | 12 | 12 |
| **DYS392** | 13 | 13 | 13 |
| **DYS643** | 10 | 10 |  |
| **DYS393** | 13 | 13 | 13 |
| **DYS458** | 18 | 18 | 18 |
| **DYS385** | 12/14 | 12/14 | 12/14 |
| **DYS456** | 15 | 15 | 15 |
| **Y_GATA_H4** | 11 | 11 | 11 |

sample 2551

PowerPlex Fusion 6C

| ***DNA locus*** | 2551  **Blood** | 2551  **Pre vasectomy semen** | 2551  **Post vasectomy seminal fluid** |
| --- | --- | --- | --- |
| **D3S1358** | 15/17 | 15/17 | 15/17 |
| **D1S1656** | 12/14 | 12/14 | 12/14 |
| **D2S441** | 11 | 11 | 11 |
| **D10S1248** | 13/15 | 13/15 | 13/15 |
| **D13S317** | 11/13 | 11/13 | 11/13 |
| **Penta E** | 10/11 | 10/11 | 10/11 |
| **D16S539** | 11/13 | 11/13 | 11/13 |
| **D18S51** | 16/18 | 16/18 | 16/18 |
| **D2S1338** | 17/23 | 17/23 | 17/23 |
| **CSF1PO** | 11 | 11 | 11 |
| **Penta D** | 9/12 | 9/12 | 9/12 |
| **TH01** | 6/10.3 | 6/10.3 | 6/10.3 |
| **vWA** | 15/17 | 15/17 | 15/17 |
| **D21S11** | 28/31.2 | 28/31.2 | 28/31.2 |
| **D7S820** | 10/12 | 10/12 | 10/12 |
| **D5S818** | 9/13 | 9/13 | 9/13 |
| **TPOX** | 11 | 11 | 11 |
| **D8S1179** | 12/15 | 12/15 | 12/15 |
| **D12S391** | 19/23 | 19/23 | 19/23 |
| **D19S433** | 13/15 | 13/15 | 13/15 |
| **SE33** | 14/29.2 | 14/29.2 | 14/29.2 |
| **D22S1045** | 12/16 | 12/16 | 16 |
| **DYS391** | 11 | 11 | 11 |
| **FGA** | 20/22 | 20/22 | 20/22 |
| **DYS576** | 18 | 18 | 18 |
| **DYS570** | 19 | 19 | 19 |
| **Amelogenina** | XY  (male) | XY  (male) | XY  (male) |

PowerPlex Y23 System

| ***DNA locus* Y STR** | 2551  **Blood** | 2551  **Pre vasectomy semen** | 2551  **Post vasectomy seminal fluid** |
| --- | --- | --- | --- |
| **DYS576** | 18 | 18 | 18 |
| **DYS389I** | 13 | 13 | 13 |
| **DYS448** | 20 | 20 | 20 |
| **DYS389II** | 30 | 30 | 30 |
| **DYS19** | 17 | 17 | 17 |
| **DYS391** | 11 | 11 | 11 |
| **DYS481** | 27 | 27 | 27 |
| **DYS549** | 11 | 11 | 11 |
| **DYS533** | 12 | 12 | 12 |
| **DYS438** | 10 | 10 | 10 |
| **DYS437** | 14 | 14 | 14 |
| **DYS570** | 19 | 19 | 19 |
| **DYS635** | 21 | 21 | 21 |
| **DYS390** | 24 | 24 | 24 |
| **DYS439** | 11 | 11 | 11 |
| **DYS392** | 11 | 11 | 11 |
| **DYS643** | 12 | 12 | 12 |
| **DYS393** | 13 | 13 | 13 |
| **DYS458** | 17 | 17 | 17 |
| **DYS385** | 12/17 | 12/17 | 12/17 |
| **DYS456** | 16 | 16 | 16 |
| **Y_GATA_H4** | 11 | 11 | 11 |

sample 2552

PowerPlex Fusion 6C

| ***DNA locus*** | 2552  **Blood** | 2552  **Pre vasectomy semen** | 2552  **Post vasectomy seminal fluid** |
| --- | --- | --- | --- |
| **D3S1358** | 15/16 | 16 | 16 |
| **D1S1656** | 14/18.3 | 14/18.3 | 14/18.3 |
| **D2S441** | 11/14 | 11/14 | 11/14 |
| **D10S1248** | 12/13 | 12/13 | 13 |
| **D13S317** | 12 | 12 | 12 |
| **Penta E** | 8/9 | 8/9 | * |
| **D16S539** | 9/12 | 9/12 | 9/12 |
| **D18S51** | 15/20 ? | 15/20 | 15/20 |
| **D2S1338** | 17/18 ? | 17/18 | 17/18 |
| **CSF1PO** | 8/12 | 8/12 | * |
| **Penta D** | 13 | 13 | 13 |
| **TH01** | 7/8 ? | 7/8 | 7/8 |
| **vWA** | 15/16/17 ? | 16/17 | 16/17 |
| **D21S11** | 29.1/31.2/32.1/32.2? | 31.2/32.2 | 32.2 |
| **D7S820** | 9/11 | 9/11 | * |
| **D5S818** | 11/13 | 11/13 | * |
| **TPOX** | 6/8 | 6/8 | * |
| **D8S1179** | 11/15 | 11/15 | 11/15 |
| **D12S391** | 17/22 ? | 17/22 | 17/22 |
| **D19S433** | 13/14 | 13/14 | 13/14 |
| **SE33** | 14/25.2 | 14/25.2 | 25.2 |
| **D22S1045** | 16 | 16 | * |
| **DYS391** | 11 | 11 | 11 |
| **FGA** | 15/19/19/24 ? | 19/24 | 19/24 |
| **DYS576** | 18 | 18 | * |
| **DYS570** | 19 | 19 | * |
| **Amelogenina** | XY  (male) | XY  (male) | XY  (male) |

PowerPlex Y23 System

| ***DNA locus* Y STR** | 2552  **Blood** | 2552  **Pre vasectomy semen** | 2552  **Post vasectomy seminal fluid** |
| --- | --- | --- | --- |
| **DYS576** | * | 18 | 18 |
| **DYS389I** | * | 13 | 13 |
| **DYS448** | * | 19 | 19 |
| **DYS389II** | * | 29 | 29 |
| **DYS19** | * | 14 |  |
| **DYS391** | * | 11 | 11 |
| **DYS481** | * | 22 | 22 |
| **DYS549** | * | 13 | 13 |
| **DYS533** | * | 12 | 12 |
| **DYS438** | * | 12 | 12 |
| **DYS437** | * | 14 | 14 |
| **DYS570** | * | 19 | 19 |
| **DYS635** | * | 24 | 24 |
| **DYS390** | * | 24 | 24 |
| **DYS439** | * | 13 | 13 |
| **DYS392** | * | 13 |  |
| **DYS643** | * | 11 |  |
| **DYS393** | * | 13 | 13 |
| **DYS458** | * | 18 | 18 |
| **DYS385** | * | 12/15 | 12/15 |
| **DYS456** | * | 16 | 16 |
| **Y_GATA_H4** | * | 12 |  |

sample 2553

PowerPlex Fusion 6C

| ***DNA locus*** | 2553  **Blood** | 2553  **Pre vasectomy semen** | 2553  **Post vasectomy seminal fluid** |
| --- | --- | --- | --- |
| **D3S1358** | 17 | 17 | 17 |
| **D1S1656** | 15/17.3 | 15/17.3 | 15/17.3 |
| **D2S441** | 14 | 14 | 14 |
| **D10S1248** | 13/15 | 13/15 | 13/15 |
| **D13S317** | 11 | 11 | 11 |
| **Penta E** | 5/7 | 5/7 | * |
| **D16S539** | 9 | 9 | 9 |
| **D18S51** | 14/17 | 14/17 | 14/17 |
| **D2S1338** | 19/22 | 19/22 | 19 |
| **CSF1PO** | 11 | 11 | * |
| **Penta D** | 8/9 | 8/9 | 9 |
| **TH01** | 7/9.3 | 7/9.3 | 7/9.3 |
| **vWA** | 17/18 | 17/18 | 17/18 |
| **D21S11** | 32.2 | 32.2 | 32.2 |
| **D7S820** | 10/11 | 10/11 |  |
| **D5S818** | 12 | 12 | * |
| **TPOX** | 10/12 | 10/12 | * |
| **D8S1179** | 12/14 | 12/14 | 12/14 |
| **D12S391** | 15/19 | 15/19 | 15/19 |
| **D19S433** | 13/15 | 13/15 | 13/15 |
| **SE33** | 19/32.2 | 19/32.2 | 19/32.2 |
| **D22S1045** | 16/17 | 16 | * |
| **DYS391** | 9 | 9 | 9 |
| **FGA** | 22 | 22 | 22 |
| **DYS576** | 15 | 15 | 15 |
| **DYS570** | 19 | 19 | * |
| **Amelogenina** | XY  (male) | XY  (male) | XY  (male) |

PowerPlex Y23 System

| ***DNA locus* Y STR** | 2553  **Blood** | 2553  **Pre vasectomy semen** | 2553  **Post vasectomy seminal fluid** |
| --- | --- | --- | --- |
| **DYS576** | 15 | 15 | 15 |
| **DYS389I** | 13 | 13 | 13 |
| **DYS448** | 20 | 20 | 20 |
| **DYS389II** | 30 | 30 | 30 |
| **DYS19** | 15 | 15 |  |
| **DYS391** | 9 | 9 | 9 |
| **DYS481** | 26 | 26 | 26 |
| **DYS549** | 12 | 12 | 12 |
| **DYS533** | 12 | 12 | 12 |
| **DYS438** | 11 | 11 |  |
| **DYS437** | 14 | 14 |  |
| **DYS570** | 19 | 19 | 19 |
| **DYS635** | 23 | 23 | 23 |
| **DYS390** | 21 | 21 | 21 |
| **DYS439** | 11 | 11 | 11 |
| **DYS392** | 11 | 11 | 11 |
| **DYS643** | 13 |  |  |
| **DYS393** | 14 | 14 | 14 |
| **DYS458** | 18 | 18 | 18 |
| **DYS385** | 15/16 | 15/16 | 15/16 |
| **DYS456** | 15 | 15 | 15 |
| **Y_GATA_H4** | 11 | 11 |  |

sample 2554

PowerPlex Fusion 6C

| ***DNA locus*** | 2554  **Blood** | 2554  **Pre vasectomy semen** | 2554  **Post vasectomy seminal fluid** |
| --- | --- | --- | --- |
| **D3S1358** | 17/18 | 17/18 | 17/18 |
| **D1S1656** | 14/16.3 | 14/16.3 | 14/16.3 |
| **D2S441** | 10/14 | 10/14 | 10/14 |
| **D10S1248** | 14/15 | 14/15 | 14/15 |
| **D13S317** | 12/13 | 12/13 | 12/13 |
| **Penta E** | 13/14 | 13/14 | 13/14 |
| **D16S539** | 11/12 | 11/12 | 11/12 |
| **D18S51** | 17/18 | 17/18 | 17/18 |
| **D2S1338** | 17/19 | 17/19 | 17/19 |
| **CSF1PO** | 10/11 | 10/11 | 10/11 |
| **Penta D** | 9/11 | 9/11 | 9/11 |
| **TH01** | 6/8 | 6/8 | 6/8 |
| **vWA** | 17/18 | 17/18 | 17/18 |
| **D21S11** | 28/31 | 28/31 | 28/31 |
| **D7S820** | 10 | 10 | 10 |
| **D5S818** | 11 | 11 | 11 |
| **TPOX** | 8/10 | 8/10 | 8/10 |
| **D8S1179** | 12/15 | 12/15 | 12/15 |
| **D12S391** | 17/19 | 17/19 | 17/19 |
| **D19S433** | 13/16 | 13/16 | 13/16 |
| **SE33** | 15/23.2 | 15/23.2 | 15/23.2 |
| **D22S1045** | 16 | 16 | 16 |
| **DYS391** | 10 | 10 | 10 |
| **FGA** | 20/23 | 20/23 | 20/23 |
| **DYS576** | 19 | 19 | 19 |
| **DYS570** | 19 | 19 | 19 |
| **Amelogenina** | XY  (male) | XY  (male) | XY  (male) |

PowerPlex Y23 System

| ***DNA locus* Y STR** | 2554  **Blood** | 2554  **Pre vasectomy semen** | 2554  **Post vasectomy seminal fluid** |
| --- | --- | --- | --- |
| **DYS576** | 19 | 19 | 19 |
| **DYS389I** | 13 | 13 | 13 |
| **DYS448** | 19 | 19 | 19 |
| **DYS389II** | 29 | 29 | 29 |
| **DYS19** | 14 | 14 | 14 |
| **DYS391** | 10 | 10 | 10 |
| **DYS481** | 23 | 23 | 23 |
| **DYS549** | 13 | 13 | 13 |
| **DYS533** | 12 | 12 | 12 |
| **DYS438** | 12 | 12 | 12 |
| **DYS437** | 15 |  | 15 |
| **DYS570** | 19 | 19 | 19 |
| **DYS635** | 23 | 23 | 23 |
| **DYS390** | 25 | 25 | 25 |
| **DYS439** | 11 | 11 | 11 |
| **DYS392** | 13 | 13 | 13 |
| **DYS643** | 10 | 10 | 10 |
| **DYS393** | 13 | 13 | 13 |
| **DYS458** | 16 | 16 | 16 |
| **DYS385** | 11/14 | 11/14 | 11/14 |
| **DYS456** | 16 | 16 | 16 |
| **Y_GATA_H4** | 11 | 11 | 11 |

sample 2556

PowerPlex Fusion 6C

| ***DNA locus*** | 2556  **Blood** | 2556  **Pre vasectomy semen** | 2556  **Post vasectomy seminal fluid** |
| --- | --- | --- | --- |
| **D3S1358** | 17 | 17 | 17 |
| **D1S1656** | 15/16 | 15/16 | 15/16 |
| **D2S441** | 11/14 | 11/14 | 11/14 |
| **D10S1248** | 14/15 | 14/15 | 14/15 |
| **D13S317** | 8/12 | 8/12 | 8/12 |
| **Penta E** | 11/12 | 11/12 | * |
| **D16S539** | 12/13 | 12/13 | 12/13 |
| **D18S51** | 15/18 | 15/18 | 15/18 |
| **D2S1338** | 17/24 | 17/24 | 17/24 |
| **CSF1PO** | 10/11 | 10/11 | 10 |
| **Penta D** | 13/15 | 13/15 | 13 |
| **TH01** | 6/7 | 6/7 | 6/7 |
| **vWA** | 17 | 17 | 17 |
| **D21S11** | 28/30 | 28/30 | 28/30 |
| **D7S820** | 11/12 | 11/12 | 11/12 |
| **D5S818** | 11/12 | 11/12 | * |
| **TPOX** | 8/10 | 8/10 | 8/10 |
| **D8S1179** | 13/16 | 13/16 | 13/16 |
| **D12S391** | 16/18 | 16/18 | 16/18 |
| **D19S433** | 15/15.2 | 15/15.2 | 15/15.2 |
| **SE33** | 23.2/30.2 | 23.2/30.2 | 23.2/30.2 |
| **D22S1045** | 16/17 | 16/17 | 17 |
| **DYS391** | 10 | 10 | 10 |
| **FGA** | 20/22 | 20/22 | 20/22 |
| **DYS576** | 16 | 16 | 16 |
| **DYS570** | 17 | 17 | * |
| **Amelogenina** | XY  (male) | XY  (male) | XY  (male) |

PowerPlex Y23 System

| ***DNA locus* Y STR** | 2556  **Blood** | 2556  **Pre vasectomy semen** | 2556  **Post vasectomy seminal fluid** |
| --- | --- | --- | --- |
| **DYS576** | 16 | 16 | 16 |
| **DYS389I** | 14 | 14 | 14 |
| **DYS448** | 19 | 19 | 19 |
| **DYS389II** | 31 | 31 | 31 |
| **DYS19** | 14 | 14 | 14 |
| **DYS391** | 10 | 10 | 10 |
| **DYS481** | 24 | 24 | 24 |
| **DYS549** | 12 | 12 | 12 |
| **DYS533** | 12 | 12 | 12 |
| **DYS438** | 9 | 9 | 9 |
| **DYS437** | 14 | 14 | 14 |
| **DYS570** | 17 | 17 | 17 |
| **DYS635** | 21 | 21 | 21 |
| **DYS390** | 23 | 23 | 23 |
| **DYS439** | 12 | 12 | 12 |
| **DYS392** | 13 | 13 | 13 |
| **DYS643** | 10 | 10 | 10 |
| **DYS393** | 13 | 13 | 13 |
| **DYS458** | 18 | 18 | 18 |
| **DYS385** | 14/15 | 1/154 | 14/15 |
| **DYS456** | 12 | 12 | 12 |
| **Y_GATA_H4** | 11 | 11 | 11 |

sample 2557

PowerPlex Fusion 6C

| ***DNA locus*** | 2557  **Blood** | 2557  **Pre vasectomy semen** | 2557  **Post vasectomy seminal fluid** |
| --- | --- | --- | --- |
| **D3S1358** | 15 | 15 | 15 |
| **D1S1656** | 17.3 | 17.3 | * |
| **D2S441** | 11/11.3 | 11/11.3 | * |
| **D10S1248** | 13/14 | 13/14 | * |
| **D13S317** | 11/12 | 11/12 | * |
| **Penta E** | 11/12 | 11/12 | * |
| **D16S539** | 9/10 | 9/10 | 9/10 |
| **D18S51** | 9/17 | 9/17 | 9/17 |
| **D2S1338** | 17/22 | 17/22 | * |
| **CSF1PO** | 11 | 11 | * |
| **Penta D** | 9 | 9 | * |
| **TH01** | 8/9 | 8/9 | 8/9 |
| **vWA** | 16/18 | 16/18 | 16 |
| **D21S11** | 29 | 29 | * |
| **D7S820** | 10/11 | 10/11 | * |
| **D5S818** | 11/12 | 11/12 | * |
| **TPOX** | 8/11 | 8/11 | * |
| **D8S1179** | 13 | 13 | 13 |
| **D12S391** | 17/18 | 17/18 | 17/18 |
| **D19S433** | 14 | 14 | 14 |
| **SE33** | 17/23.2 | 17/23.2 | * |
| **D22S1045** | 11 | 11/x | * |
| **DYS391** | 10 | 10 | 10 |
| **FGA** | 20/26 | 20/26 | 20/26 |
| **DYS576** | 17 | 17 | * |
| **DYS570** | 18 | 18 | * |
| **Amelogenina** | XY  (male) | XY  (male) | XY  (male) |

PowerPlex Y23 System

| ***DNA locus* Y STR** | 2557  **Blood** | 2557  **Pre vasectomy semen** | 2557  **Post vasectomy seminal fluid** |
| --- | --- | --- | --- |
| **DYS576** | 17 | 17 | 17 |
| **DYS389I** | 12 | 12 |  |
| **DYS448** | 19 | 19 |  |
| **DYS389II** | 28 | 28 |  |
| **DYS19** | 14 | 14 |  |
| **DYS391** | 10 | 10 | 10 |
| **DYS481** | 22 | 22 | 22 |
| **DYS549** | 13 | 13 |  |
| **DYS533** | 12 | 12 |  |
| **DYS438** | 12 | 12 |  |
| **DYS437** | 15 |  |  |
| **DYS570** | 18 | 18 |  |
| **DYS635** | 23 | 23 |  |
| **DYS390** | 23 | 23 |  |
| **DYS439** | 13 | 13 |  |
| **DYS392** | 13 | 13 |  |
| **DYS643** | 10 | 10 |  |
| **DYS393** | 13 | 13 | 13 |
| **DYS458** | 18 | 18 | 18 |
| **DYS385** | 11/14 | 11/14 | 14 |
| **DYS456** | 16 | 16 |  |
| **Y_GATA_H4** | 11 | 11 |  |

sample 2558

PowerPlex Fusion 6C

| ***DNA locus*** | 2558  **Blood** | 2558  **Pre vasectomy semen** | 2558  **Post vasectomy seminal fluid** |
| --- | --- | --- | --- |
| **D3S1358** | 16/17 | 16/17 | 16/17 |
| **D1S1656** | 11/16.3 | 11/16.3 | 11/16.3 |
| **D2S441** | 11 | 11 | 11 |
| **D10S1248** | 15/16 | 15/16 | 15/16 |
| **D13S317** | OL | 11 | 11 |
| **Penta E** | 7/15 | 7/15 | 7/15 |
| **D16S539** | 9/11 | 9/11 | 9/11 |
| **D18S51** | 12/13 | 12/13 | 12/13 |
| **D2S1338** | 17/26 | 17/26 | 17/26 |
| **CSF1PO** | 7 | 7/8 | 7/8 |
| **Penta D** | 10/12 | 10/12 | 10/12 |
| **TH01** | 7/8 | 7/8 | 7/8 |
| **vWA** | 15/16 | 15/16 | 15/16 |
| **D21S11** | 29.1/30/34 | 30/34 | 30/34 |
| **D7S820** | 8/9 | 8/9 | 8/9 |
| **D5S818** | OL/12/13 | 12/13 | 12/13 |
| **TPOX** | 8 | 8 | 8 |
| **D8S1179** | 14/15 | 14/15 | 14/15 |
| **D12S391** | 16/18 | 16/18 | 16/18 |
| **D19S433** | 12/13.2 | 12/13.2 | 12/13.2 |
| **SE33** | 20/24.2 | 20/24.2 | 20/24.2 |
| **D22S1045** | 16/18 | 16/18 | 16/18 |
| **DYS391** | 10 | 10 | 10 |
| **FGA** | 25 | 25 | 25 |
| **DYS576** | 16 | 16 | 16 |
| **DYS570** | 21 | 21 | 21 |
| **Amelogenina** | XY  (male) | XY  (male) | XY  (male) |

PowerPlex Y23 System

| ***DNA locus* Y STR** | 2558  **Blood** | 2558  **Pre vasectomy semen** | 2558  **Post vasectomy seminal fluid** |
| --- | --- | --- | --- |
| **DYS576** | 16 | 16 | 16 |
| **DYS389I** | 12 | 12 | 12 |
| **DYS448** | 19 | 19 | 19 |
| **DYS389II** | 28 | 28 | 28 |
| **DYS19** | 14 | 14 | 14 |
| **DYS391** | 10 | 10 | 10 |
| **DYS481** | 26 | 26 | 26 |
| **DYS549** | 12 | 12 | 12 |
| **DYS533** | 11 | 11 | 11 |
| **DYS438** | 10 | 10 | 10 |
| **DYS437** | 15 | 15 | 15 |
| **DYS570** | 21 | 21 | 21 |
| **DYS635** | 22 | 22 | 22 |
| **DYS390** | 24 | 24 | 24 |
| **DYS439** | 11 | 11 | 11 |
| **DYS392** | 13 | 13 | 13 |
| **DYS643** | 12 | 12 | 12 |
| **DYS393** | 13 | 13 | 13 |
| **DYS458** | 16 | 16 | 16 |
| **DYS385** | 14/15 | 14/15 | 14/15 |
| **DYS456** | 14 | 14 | 14 |
| **Y_GATA_H4** | 11 | 11 | 11 |

sample 2561

PowerPlex Fusion 6C

| ***DNA locus*** | 2561  **Blood** | 2561  **Pre vasectomy semen** | 2561  **Post vasectomy seminal fluid** |
| --- | --- | --- | --- |
| **D3S1358** | 17/19 | 17/19 | 17/19 |
| **D1S1656** | 14/15 | 14/15 | 14/15 |
| **D2S441** | 10/11 | 10/11 | 10/11 |
| **D10S1248** | 13/14 | 13/14 | 13/14 |
| **D13S317** | 8/13 | 8/13 | 8/13 |
| **Penta E** | 11/13 | 11/13 | 13 |
| **D16S539** | 11/12 | 11/12 | 11/12 |
| **D18S51** | 12 | 12 | 12 |
| **D2S1338** | 24 | 24 | 24 |
| **CSF1PO** | 10/11 | 10/11 | 10/11 |
| **Penta D** | 9/12 | 9/12 | 9/12 |
| **TH01** | 7/8 | 7/8 | 7/8 |
| **vWA** | 18 | 18 | 18 |
| **D21S11** | 28/29 | 28/29 | 28/29 |
| **D7S820** | 11/13 | 11/13 | 11/13 |
| **D5S818** | 11 | 11 | 11 |
| **TPOX** | 8/11 | 8/11 | 8/11 |
| **D8S1179** | 10/14 | 10/14 | 10/14 |
| **D12S391** | 18/21 | 18/21 | 18/21 |
| **D19S433** | 13/15 | 13/15 | 13/15 |
| **SE33** | 15/18 | 15/18 | 15/18 |
| **D22S1045** | 15/16 | 15/16 | 16 |
| **DYS391** | 10 | 10 | 10 |
| **FGA** | 24/25 | 24/25 | 24/25 |
| **DYS576** | 17 | 17 | 17 |
| **DYS570** | 19 | 19 | 19 |
| **Amelogenina** | XY  (male) | XY  (male) | XY  (male) |

PowerPlex Y23 System

| ***DNA locus* Y STR** | 2561  **Blood** | 2561  **Pre vasectomy semen** | 2561  **Post vasectomy seminal fluid** |
| --- | --- | --- | --- |
| **DYS576** | 17 | 17 | 17 |
| **DYS389I** | 12 | 12 | 12 |
| **DYS448** | 20 | 20 | 20 |
| **DYS389II** | 29 | 29 | 29 |
| **DYS19** | 14 | 14 |  |
| **DYS391** | 10 | 10 | 10 |
| **DYS481** | 25 | 25 | 25 |
| **DYS549** | 12 | 12 | 12 |
| **DYS533** | 12 | 12 | 12 |
| **DYS438** | 10 | 10 | 10 |
| **DYS437** | 16 | 16 |  |
| **DYS570** | OL | 19 | 19 |
| **DYS635** | 22 | 22 | 22 |
| **DYS390** | 22 | 22 | 22 |
| **DYS439** | 11 | 11 | 11 |
| **DYS392** | 11 | 11 | 7 |
| **DYS643** | 13 | 13 | 13 |
| **DYS393** | OL | 12 | 12 |
| **DYS458** | 15 | 15 | 15 |
| **DYS385** | 13/16 | 13/16 | 13/16 |
| **DYS456** | 14 | 14 | 14 |
| **Y_GATA_H4** | 11 | 11 | 11 |

sample 2562

PowerPlex Fusion 6C

| ***DNA locus*** | 2562  **Blood** | 2562  **Pre vasectomy semen** | 2562  **Post vasectomy seminal fluid** |
| --- | --- | --- | --- |
| **D3S1358** | 15/17 | 15/17 | 15/17 |
| **D1S1656** | 15/17.3 | 15/17.3 | 15/17.3 |
| **D2S441** | 11/11.3 | 11/11.3 | 11/11.3 |
| **D10S1248** | 16 | 16 | 16 |
| **D13S317** | 12/14 | 12/14 | 12/14 |
| **Penta E** | 13/16 | 13/16 | 13/16 |
| **D16S539** | 9/11 | 9/11 | 9/11 |
| **D18S51** | 13/14 | 13/14 | 13/14 |
| **D2S1338** | 16/25 | 16/25 | 16/25 |
| **CSF1PO** | 11/12 | 11/12 | 11/12 |
| **Penta D** | 10/13 | 10/13 | 10/13 |
| **TH01** | 6/7 | 6/7 | 6/7 |
| **vWA** | 16/17 | 16/17 | 16/17 |
| **D21S11** | 32.2 | 32.2 | 32.2 |
| **D7S820** | 12/14 | 12/14 | 12/14 |
| **D5S818** | 11 | 11 | 11 |
| **TPOX** | 8/11 | 8/11 | 11 |
| **D8S1179** | 14 | 14 | 14 |
| **D12S391** | 18/22 | 18/22 | 18/22 |
| **D19S433** | 15/16 | 15/16 | 15/16 |
| **SE33** | 15/17 | 15/17 | 15/17 |
| **D22S1045** | 15 | 15 | 15 |
| **DYS391** | 10 | 10 | 10 |
| **FGA** | 21/24 | 21/24 | 21/24 |
| **DYS576** | 19 | 19 | 19 |
| **DYS570** | 18 | 18 | 18 |
| **Amelogenina** | XY  (male) | XY  (male) | XY  (male) |

PowerPlex Y23 System

| ***DNA locus* Y STR** | 2562  **Blood** | 2562  **Pre vasectomy semen** | 2562  **Post vasectomy seminal fluid** |
| --- | --- | --- | --- |
| **DYS576** | 19 | 19 | 19 |
| **DYS389I** | 14 | 14 | 14 |
| **DYS448** | 20 | 20 | 20 |
| **DYS389II** | 31 | 31 | 31 |
| **DYS19** | 14 | 14 | 14 |
| **DYS391** | 10 | 10 | 10 |
| **DYS481** | 23 | 23 | 23 |
| **DYS549** | 13 | 13 | 13 |
| **DYS533** | 12 | 12 | 12 |
| **DYS438** | 10 | 10 | 10 |
| **DYS437** | 14 | 14 | 14 |
| **DYS570** | 18 | 18 | 18 |
| **DYS635** | 20 | 20 | 20 |
| **DYS390** | 23 | 23 | 23 |
| **DYS439** | 12 | 12 | 12 |
| **DYS392** | 11 | 11 | 11 |
| **DYS643** | 12 | 12 | 12 |
| **DYS393** | 13 | 13 | 13 |
| **DYS458** | 16 | 16 | 16 |
| **DYS385** | 18/20 | 18/20 | 18/20 |
| **DYS456** | 15 | 15 | 15 |
| **Y_GATA_H4** | 13 | 13 | 13 |

sample 2563

PowerPlex Fusion 6C

| ***DNA locus*** | 2563  **Blood** | 2563  **Pre vasectomy semen** | 2563  **Post vasectomy seminal fluid** |
| --- | --- | --- | --- |
| **D3S1358** | 17 | 17 | 17 |
| **D1S1656** | 14.3/15 | 14.3/15 | 14.3/15 |
| **D2S441** | 11/14 | 11/14 | 11/14 |
| **D10S1248** | 11/14 | 11/14 | 11/14 |
| **D13S317** | 12/13 | 12/13 | 12/13 |
| **Penta E** | 9/12 | 9/12 | * |
| **D16S539** | 9/10 | 9/10 | 9/10 |
| **D18S51** | 13/16 | 13/16 | 13/16 |
| **D2S1338** | 20/23 | 20/23 | 20/23 |
| **CSF1PO** | 10 | 10 | 10 |
| **Penta D** | 5/9.2 | 5/9.2 | 5/9.2 |
| **TH01** | 9/9.3 | 9/9.3 | 9/9.3 |
| **vWA** | 17/19 | 17/19 | 17/19 |
| **D21S11** | 30 | 30 | 30 |
| **D7S820** | 10/11 | 10/11 | 10/11 |
| **D5S818** | 11/12 | 11/12 | 11 |
| **TPOX** | 8/11 | 8 | * |
| **D8S1179** | 11/14 | 11/14 | 11/14 |
| **D12S391** | 18/19 | 18/19 | 18/19 |
| **D19S433** | 13/14 | 13/14 | 13/14 |
| **SE33** | 15/28.2 | 15/28.2 | 15/28.2 |
| **D22S1045** | 11/16 | 11/16 | 11/16 |
| **DYS391** | 11 | 11 | 11 |
| **FGA** | 22/23 | 22/23 | 22/23 |
| **DYS576** | 18 | 18 | 18 |
| **DYS570** | 18 | 18 | * |
| **Amelogenina** | XY  (male) | XY  (male) | XY  (male) |

PowerPlex Y23 System

| ***DNA locus* Y STR** | 2563  **Blood** | 2563  **Pre vasectomy semen** | 2563  **Post vasectomy seminal fluid** |
| --- | --- | --- | --- |
| **DYS576** | 18 | 18 | 18 |
| **DYS389I** | 13 | 13 | 13 |
| **DYS448** | 20 | 20 | 20 |
| **DYS389II** | 29 | 29 | 29 |
| **DYS19** | 14 | 14 | 14 |
| **DYS391** | 11 | 11 | 11 |
| **DYS481** | 25 | 25 | 25 |
| **DYS549** | 12 | 12 | 12 |
| **DYS533** | 12 | 12 | 12 |
| **DYS438** | 10 | 10 | 10 |
| **DYS437** | 14 | 14 | 14 |
| **DYS570** | 18 | 18 | 18 |
| **DYS635** | 21 | 21 | 21 |
| **DYS390** | 23 | 23 | 23 |
| **DYS439** | 11 | 11 | 11 |
| **DYS392** | 11 | 11 | 11 |
| **DYS643** | 9 | 9 | 9 |
| **DYS393** | 12 | 12 | 12 |
| **DYS458** | 18.2 | 18.2 | 18.2 |
| **DYS385** | 13 | 13 | 13 |
| **DYS456** | 13 | 13 | 13 |
| **Y_GATA_H4** | 12 | 12 | 12 |

sample 2564

PowerPlex Fusion 6C

| ***DNA locus*** | 2564  **Blood** | 2564  **Pre vasectomy semen** | 2564  **Post vasectomy seminal fluid** |
| --- | --- | --- | --- |
| **D3S1358** | 14/15 | 14/15 | 14/15 |
| **D1S1656** | 14 | 14 | 14 |
| **D2S441** | 10/11 | 10/11 | 10/11 |
| **D10S1248** | 15/16 | 15/16 | 15/16 |
| **D13S317** | 12/13 | 12/13 | 12/13 |
| **Penta E** | 10/13 | 10/13 | 10/13 |
| **D16S539** | 9/10 | 9/10 | 9/10 |
| **D18S51** | 16/17 | 16/17 | 16/17 |
| **D2S1338** | 23/25 | 23/25 | 23/25 |
| **CSF1PO** | 8/10 | 8/10 | 8/10 |
| **Penta D** | 12 | 12 | 12 |
| **TH01** | 6/7 | 6/7 | 6/7 |
| **vWA** | 15/18 | 15/18 | 15/18 |
| **D21S11** | 30 | 30 | 30 |
| **D7S820** | 8/11 | 8/11 | 8/11 |
| **D5S818** | 12/13 | 12/13 | 12/13 |
| **TPOX** | 8/9 | 8/9 | 8 |
| **D8S1179** | 12/14 | 12/14 | 12/14 |
| **D12S391** | 15/22 | 15/22 | 15/22 |
| **D19S433** | 13/16 | 13/16 | 13/16 |
| **SE33** | 12.2/19 | 12.2/19 | 12.2/19 |
| **D22S1045** | 15/16 | 15/16 | 15/16 |
| **DYS391** | 10 | 10 | 10 |
| **FGA** | 20/21 | 20/21 | 20/21 |
| **DYS576** | 16 | 16 | 16 |
| **DYS570** | 18 | 18 | 18 |
| **Amelogenina** | XY  (male) | XY  (male) | XY  (male) |

PowerPlex Y23 System

| ***DNA locus* Y STR** | 2564  **Blood** | 2564  **Pre vasectomy semen** | 2564  **Post vasectomy seminal fluid** |
| --- | --- | --- | --- |
| **DYS576** | 16 | 16 | 16 |
| **DYS389I** | 12 | 12 | 12 |
| **DYS448** | 20 | 20 | 20 |
| **DYS389II** | 28 | 28 | 28 |
| **DYS19** | 14 | 14 | 14 |
| **DYS391** | 10 | 10 | 10 |
| **DYS481** | 28 | 28 | 28 |
| **DYS549** | 12 | 12 | 12 |
| **DYS533** | 11 | 11 | 11 |
| **DYS438** | 10 | 10 | 10 |
| **DYS437** | 16 | 16 |  |
| **DYS570** | 18 | 18 | 18 |
| **DYS635** | 23 | 23 | 23 |
| **DYS390** | 23 | 23 | 23 |
| **DYS439** | 11 | 11 | 11 |
| **DYS392** | 11 | 11 | 11 |
| **DYS643** | 12 | 12 | 12 |
| **DYS393** | 13 | 13 | 13 |
| **DYS458** | 16 | 16 | 16 |
| **DYS385** | 13/14 | 13/14 | 13/14 |
| **DYS456** | 14 | 14 | 14 |
| **Y_GATA_H4** | 11 | 11 | 11 |

sample 2565

PowerPlex Fusion 6C

| ***DNA locus*** | 2565  **Blood** | 2565  **Pre vasectomy semen** | 2565  **Post vasectomy seminal fluid** |
| --- | --- | --- | --- |
| **D3S1358** | 17/18 | 17/18 | 17/18 |
| **D1S1656** | 13/20.3 | 13/20.3 | 13/20.3 |
| **D2S441** | 10/11 | 10/11 | 10/11 |
| **D10S1248** | 14/16 | 14/16 | 14/16 |
| **D13S317** | 8/9 | 8/9 | 8/9 |
| **Penta E** | 9/12 | 9/12 | * |
| **D16S539** | 11 | 11 | 11 |
| **D18S51** | 13/19 | 13/19 | 13/19 |
| **D2S1338** | 23 | 23 | 23 |
| **CSF1PO** | 10/11 | 10/11 | 10 |
| **Penta D** | 9/11 | 9/11 | 11 |
| **TH01** | 7/9.3 | 7/9.3 | 7/9.3 |
| **vWA** | 14/16 | 14/16 | 14/16 |
| **D21S11** | 27/30 | 27/30 | 27/30 |
| **D7S820** | 9/10 | 9/10 | * |
| **D5S818** | 12 | 12 | 12 |
| **TPOX** | 8/11 | 8/11 | * |
| **D8S1179** | 14 | 14 | 14 |
| **D12S391** | 18/19 | 18/19 | 18/19 |
| **D19S433** | 13/16 | 13/16 | 13/16 |
| **SE33** | 14/20 | 14/20 | 14/20 |
| **D22S1045** | 15/16 | 15/16 | 17 |
| **DYS391** | 11 | 11 | 11 |
| **FGA** | 23.2/24 | 23.2/24 | 23.2/24 |
| **DYS576** | 17 | 17 | * |
| **DYS570** | 18 | 18 | * |
| **Amelogenina** | XY  (male) | XY  (male) | XY  (male) |

PowerPlex Y23 System

| ***DNA locus* Y STR** | 2565  **Blood** | 2565  **Pre vasectomy semen** | 2565  **Post vasectomy seminal fluid** |
| --- | --- | --- | --- |
| **DYS576** | 17 | 17 | 17 |
| **DYS389I** | 12 | 12 | 12 |
| **DYS448** | 21 | 21 | 21 |
| **DYS389II** | 29 | 29 | 29 |
| **DYS19** | 16 | 16 | 16 |
| **DYS391** | 11 | 11 | 11 |
| **DYS481** | 21 | 21 | 21 |
| **DYS549** | 11 | 11 | 11 |
| **DYS533** | 10 | 10 | 10 |
| **DYS438** | 10 | 10 | 10 |
| **DYS437** | 16 | 16 | 16 |
| **DYS570** | 18 | 18 | 18 |
| **DYS635** | 23 | 23 | 23 |
| **DYS390** | 21 | 21 | 21 |
| **DYS439** | 12 | 12 | 12 |
| **DYS392** | 11 | 11 | 11 |
| **DYS643** | 12 | 12 |  |
| **DYS393** | 13 | 13 | 13 |
| **DYS458** | 16 | 16 | 16 |
| **DYS385** | 15 | 15 | 15 |
| **DYS456** | 14 | 14 | 14 |
| **Y_GATA_H4** | 11 | 11 | 11 |

sample 2567

PowerPlex Fusion 6C

| ***DNA locus*** | 2567  **Blood** | 2567  **Pre vasectomy semen** | 2567  **Post vasectomy seminal fluid** |
| --- | --- | --- | --- |
| **D3S1358** | 17/18 | 17/18 | 17/18 |
| **D1S1656** | 12/16.3 | 12/16.3 | 12/16.3 |
| **D2S441** | 11 | 11 | 11 |
| **D10S1248** | 13/14 | 13/14 | 13/14 |
| **D13S317** | 8/11 | 8/11 | 8/11 |
| **Penta E** | 11/14 | 11/14 | * |
| **D16S539** | 9/11 | 9/11 | 9/11 |
| **D18S51** | 14/20 | 14/20 | 14/20 |
| **D2S1338** | 17/25 | 17/25 | 17/25 |
| **CSF1PO** | 12 | 12 | 12 |
| **Penta D** | 9/13 | 9/13 | 13 |
| **TH01** | 7/9 | 7/9 | 7/9 |
| **vWA** | 16 | 16 | 16 |
| **D21S11** | 29/31.2 | 29/31.2 | 29/31.2 |
| **D7S820** | 8/11 | 8/11 | 8/11 |
| **D5S818** | 10/12 | 10/12 | 10/12 |
| **TPOX** | 8/11 | 8/11 | * |
| **D8S1179** | 10/14 | 10/14 | 10/14 |
| **D12S391** | 20/22 | 20/22 | 20/22 |
| **D19S433** | 13/13.2 | 13/13.2 | 13/13.2 |
| **SE33** | 18/19 | 18/19 | 18/19 |
| **D22S1045** | 15 | 15 | * |
| **DYS391** | 11 | 11 | 11 |
| **FGA** | 20/25 | 20/25 | 20/25 |
| **DYS576** | 17 | 17 | 17 |
| **DYS570** | 19 | 19 | * |
| **Amelogenina** | XY  (male) | XY  (male) | XY  (male) |

PowerPlex Y23 System

| ***DNA locus* Y STR** | 2567  **Blood** | 2567  **Pre vasectomy semen** | 2567  **Post vasectomy seminal fluid** |
| --- | --- | --- | --- |
| **DYS576** | 17 | 17 | 17 |
| **DYS389I** | 12 | 12 | 12 |
| **DYS448** | 19 | 19 | 19 |
| **DYS389II** | 28 | 28 |  |
| **DYS19** | 15 | 15 |  |
| **DYS391** | 11 | 11 | 11 |
| **DYS481** | 23 | 23 | 23 |
| **DYS549** | 13 | 13 | 13 |
| **DYS533** | 12 | 12 | 12 |
| **DYS438** | 9 | 9 | 9 |
| **DYS437** | 16 | 16 |  |
| **DYS570** | 19 | 19 | 19 |
| **DYS635** | 21 | 21 |  |
| **DYS390** | 24 | 24 |  |
| **DYS439** | 12 | 12 |  |
| **DYS392** | 11 | 11 |  |
| **DYS643** | 9 | 9 |  |
| **DYS393** | 12 | 12 | 12 |
| **DYS458** | 15 | 15 | 15 |
| **DYS385** | 13/16 | 13/16 | 13/16 |
| **DYS456** | 13 | 13 | 13 |
| **Y_GATA_H4** | 11 | 11 |  |

sample 2568

PowerPlex Fusion 6C

| ***DNA locus*** | 2568  **Blood** | 2568  **Pre vasectomy semen** | 2568  **Post vasectomy seminal fluid** |
| --- | --- | --- | --- |
| **D3S1358** | 15/16 | 15/16 | 15/16 |
| **D1S1656** | 17.3/18.3 | 17.3/18.3 | 17.3/18.3 |
| **D2S441** | 10/14 | 10/14 | 10/14 |
| **D10S1248** | 14/15 | 14/15 | 14/15 |
| **D13S317** | 9/12 | 9/12 | 9/12 |
| **Penta E** | 7 | 7 | 7 |
| **D16S539** | 11 | 11 | 11 |
| **D18S51** | 15/18 | 15/18 | 15/18 |
| **D2S1338** | 19/22 | 19/22 | 19/22 |
| **CSF1PO** | 12 | 12 | 12 |
| **Penta D** | 10/14 | 10/14 | 10 |
| **TH01** | 7/9 | 7/9 | 7/9 |
| **vWA** | 17/19 | 17/19 | 17/19 |
| **D21S11** | 27/28 | 27/28 | 27/28 |
| **D7S820** | 11 | 11 | 11 |
| **D5S818** | 7/13 | 7/13 | 7/13 |
| **TPOX** | 9 | 9 | * |
| **D8S1179** | 10/14 | 10/14 | 10/14 |
| **D12S391** | 18 | 18 | 18 |
| **D19S433** | 12/13 | 12/13 | 12/13 |
| **SE33** | 19/26.2 | 19/26.2 | 19/26.2 |
| **D22S1045** | 15/17 | 17 | * |
| **DYS391** | 10 | 10 | 10 |
| **FGA** | 19/22 | 19/22 | 19/22 |
| **DYS576** | 18 | 18 | 18 |
| **DYS570** | 16 | 16 | 16 |
| **Amelogenina** | XY  (male) | XY  (male) | XY  (male) |

PowerPlex Y23 System

| ***DNA locus* Y STR** | 2568  **Blood** | 2568  **Pre vasectomy semen** | 2568  **Post vasectomy seminal fluid** |
| --- | --- | --- | --- |
| **DYS576** | 18 | 18 | 18 |
| **DYS389I** | 13 | 13 | 13 |
| **DYS448** | 20 | 20 | 20 |
| **DYS389II** | 30 | 30 | 30 |
| **DYS19** | 15 | 15 | 15 |
| **DYS391** | 10 | 10 | 10 |
| **DYS481** | 25 | 25 | 25 |
| **DYS549** | 11 | 11 | 11 |
| **DYS533** | 11 | 11 | 11 |
| **DYS438** | 12 | 12 | 12 |
| **DYS437** | 13 | 13 |  |
| **DYS570** | 16 | 16 | 16 |
| **DYS635** | 22 | 22 | 22 |
| **DYS390** | 21 | 21 | 21 |
| **DYS439** | 12 | 12 | 12 |
| **DYS392** | 11 | 11 | 11 |
| **DYS643** | 13 | 13 | OL |
| **DYS393** | 15 | 15 | 15 |
| **DYS458** | 15 | 15 | 15 |
| **DYS385** | 16/17 | 16/17 | 16/17 |
| **DYS456** | 16 | 16 | 16 |
| **Y_GATA_H4** | 11 | 11 | 11 |

sample 2603

PowerPlex Fusion 6C

| ***DNA locus*** | 2603  **Blood** | 2603  **Pre vasectomy semen** | 2603  **Post vasectomy seminal fluid** |
| --- | --- | --- | --- |
| **D3S1358** | 15/16 | 15/16 | 15/16 |
| **D1S1656** | 11/18.3 | 11/18.3 | 11/18.3 |
| **D2S441** | 9/14 | 9/14 | 9/14 |
| **D10S1248** | 13/14 | 13/14 | 13/14 |
| **D13S317** | 9/12 | 9/12 | 9/12 |
| **Penta E** | 11/17 | 11/17 | 11 |
| **D16S539** | 9/11 | 9/11 | 9/11 |
| **D18S51** | 9/14 | 9/14 | 9/14 |
| **D2S1338** | 17/23 | 17/23 | 17/23 |
| **CSF1PO** | 10/12 | 10/12 | 10/12 |
| **Penta D** | 11/12 | 11/12 | 11 |
| **TH01** | 8 | 7.3/8 | 8 |
| **vWA** | 16/19 | 16/19 | 16/19 |
| **D21S11** | 31.2/32.2 | 31.2/32.2 | 31.2/32.2 |
| **D7S820** | 8 | 8 | 8 |
| **D5S818** | 12/13 | 12/13 | 12/13 |
| **TPOX** | 9/10 | 9/10 | 9/10 |
| **D8S1179** | 10/16 | 10/16 | 10/16 |
| **D12S391** | 15/17.3 | 15/17.3 | 15/17.3 |
| **D19S433** | 13/13.2 | 13/13.2 | 13/13.2 |
| **SE33** | 21/22 | 21/22 | 21/22 |
| **D22S1045** | 16 | 16 | 16 |
| **DYS391** | 10 | 10 | 10 |
| **FGA** | 24/29 | 24/29 | 24/29 |
| **DYS576** | 18 | 18 | 18 |
| **DYS570** | 18 | 18 | 18 |
| **Amelogenina** | XY  (male) | XY  (male) | XY  (male) |

PowerPlex Y23 System

| ***DNA locus* Y STR** | 2603  **Blood** | 2603  **Pre vasectomy semen** | 2603  **Post vasectomy seminal fluid** |
| --- | --- | --- | --- |
| **DYS576** | 18 | 18 | 18 |
| **DYS389I** | 13 | 13 | 13 |
| **DYS448** | 19 | 19 | 19 |
| **DYS389II** | 29 | 29 | 29 |
| **DYS19** | 14 | 14 | 14 |
| **DYS391** | 10 | 10 | 10 |
| **DYS481** | 22 | 22 | 22 |
| **DYS549** | 12 | 12 | 12 |
| **DYS533** | 12 | 12 | 12 |
| **DYS438** | 12 | 12 | 12 |
| **DYS437** | 15 | 15 | 15 |
| **DYS570** | 18 | 18 | 18 |
| **DYS635** | 23 | 23 | 23 |
| **DYS390** | 24 | 24 | 24 |
| **DYS439** | 12 | 12 | 12 |
| **DYS392** | 13 | 13 | 13 |
| **DYS643** | 9 | 9 | 9 |
| **DYS393** | 13 | 13 | 13 |
| **DYS458** | 17 | 17 | 17 |
| **DYS385** | 11/14 | 11/14 | 11/14 |
| **DYS456** | 16 | 16 | 16 |
| **Y_GATA_H4** | 11 | 11 | 11 |

sample 2610

PowerPlex Fusion 6C

| ***DNA locus*** | 2610  **Blood** | 2610  **Pre vasectomy semen** | 2610  **Post vasectomy seminal fluid** |
| --- | --- | --- | --- |
| **D3S1358** | 16 | 16 | 16 |
| **D1S1656** | 16.3/17.3 | 16.3/17.3 | 16.3/17.3 |
| **D2S441** | 10/13 | 10/13 | 10/13 |
| **D10S1248** | 11/14 | 11/14 | 11/14 |
| **D13S317** | 11/12 | 11/12 | 11/12 |
| **Penta E** | 8/13 | 8/13 | 8/13 |
| **D16S539** | 9/13 | 9/13 | 9/13 |
| **D18S51** | 18 | 18 | 18 |
| **D2S1338** | 19/26 | 19/26 | 19/26 |
| **CSF1PO** | 11 | 11 | 11 |
| **Penta D** | 10/13 | 10/13 | 10/13 |
| **TH01** | 6/7 | 6/7 | 6/7 |
| **vWA** | 15/17 | 15/17 | 15/17 |
| **D21S11** | 29/32 | 29/32 | 29/32 |
| **D7S820** | 10 | 10 | 10 |
| **D5S818** | 11/12 | 11/12 | 11/12 |
| **TPOX** | 8/9 | 8/9 | 8/9 |
| **D8S1179** | 13/16 | 13/16 | 13/16 |
| **D12S391** | 15/18 | 15/18 | 15/18 |
| **D19S433** | 13 | 13 | 13 |
| **SE33** | 16/19 | 16/19 | 16/19 |
| **D22S1045** | 16 | 16 | 16 |
| **DYS391** | 10 | 10 | 10 |
| **FGA** | 24 | 24 | 24 |
| **DYS576** | 15 | 15 | 15 |
| **DYS570** | 20 | 20 | 20 |
| **Amelogenina** | XY  (male) | XY  (male) | XY  (male) |

PowerPlex Y23 System

| ***DNA locus* Y STR** | 2610  **Blood** | 2610  **Pre vasectomy semen** | 2610  **Post vasectomy seminal fluid** |
| --- | --- | --- | --- |
| **DYS576** | 15 | 15 | 15 |
| **DYS389I** | 13 | 13 | 13 |
| **DYS448** | 21 | 21 | 21 |
| **DYS389II** | 30 | 30 | 30 |
| **DYS19** | 15 | 15 | 15 |
| **DYS391** | 10 | 10 | 10 |
| **DYS481** | 28 | 28 | 28 |
| **DYS549** | 10 | 10 | 10 |
| **DYS533** | 11 | 11 | 11 |
| **DYS438** | 11 | 11 | 11 |
| **DYS437** | 14 | 14 | 14 |
| **DYS570** | 20 | 20 | 20 |
| **DYS635** | 21 | 21 | 21 |
| **DYS390** | 21 | 21 | 21 |
| **DYS439** | 11 | 11 | 11 |
| **DYS392** | 11 | 11 | 11 |
| **DYS643** | 13 | 13 | 13 |
| **DYS393** | 13 | 13 | 13 |
| **DYS458** | 15 | 15 | 15 |
| **DYS385** | 16/18 | 16/18 | 16/18 |
| **DYS456** | 15 | 15 | 15 |
| **Y_GATA_H4** | 12 | 12 | 12 |

sample 2612

PowerPlex Fusion 6C

| ***DNA locus*** | 2612  **Blood** | 2612  **Pre vasectomy semen** | 2612  **Post vasectomy seminal fluid** |
| --- | --- | --- | --- |
| **D3S1358** | 17 | 17 | 17 |
| **D1S1656** | 14 | 14 | 14 |
| **D2S441** | 11.3/14 | 11.3/14 | 11.3/14 |
| **D10S1248** | 13/14 | 13/14 | 13/14 |
| **D13S317** | 11 | 11 | 11 |
| **Penta E** | 5/10 | 5/10 | 5/10 |
| **D16S539** | 10/12 | 10/12 | 10/12 |
| **D18S51** | 13/15 | 13/15 | 13/15 |
| **D2S1338** | 20 | 20 | 20 |
| **CSF1PO** | 7/10 | 7/10 | 7/10 |
| **Penta D** | 9/11 | 9/11 | 9/11 |
| **TH01** | 7 | 7 | 7 |
| **vWA** | 14/15 | 14/15 | 14/15 |
| **D21S11** | 30/32.2 | 30/32.2 | 30/32.2 |
| **D7S820** | 9/10 | 9/10 | 9/10 |
| **D5S818** | 12/13 | 12/13 | 12/13 |
| **TPOX** | 6/11 | 6/11 | 6/11 |
| **D8S1179** | 12/14 | 12/14 | 12/14 |
| **D12S391** | 18/19 | 18/19 | 18/19 |
| **D19S433** | 15 | 15 | 15 |
| **SE33** | OL/OL | 29.2/30.2 | 29.2/30.2 |
| **D22S1045** | 15 | 15 | 15 |
| **DYS391** | 10 | 10 | 10 |
| **FGA** | 20/23 | 20/23 | 20/23 |
| **DYS576** | 15 | 15 | 15 |
| **DYS570** | 17 | 17 | 17 |
| **Amelogenina** | XY  (male) | XY  (male) | XY  (male) |

PowerPlex Y23 System

| ***DNA locus* Y STR** | 2612  **Blood** | 2612  **Pre vasectomy semen** | 2612  **Post vasectomy seminal fluid** |
| --- | --- | --- | --- |
| **DYS576** | 15 | 15 | 15 |
| **DYS389I** | 15 | 15 | 15 |
| **DYS448** | 19 | 19 | 19 |
| **DYS389II** | 32 | 32 | 32 |
| **DYS19** | 14 | 14 | 14 |
| **DYS391** | 10 | 10 | 10 |
| **DYS481** | 23 | 23 | 23 |
| **DYS549** | 12 | 12 | 12 |
| **DYS533** | 12 | 12 | 12 |
| **DYS438** | 9 | 9 | 9 |
| **DYS437** | 14 | 14 | 14 |
| **DYS570** | 17 | 17 | 17 |
| **DYS635** | 20 | 20 | 20 |
| **DYS390** | 23 | 23 | 23 |
| **DYS439** | 11 | 11 | 11 |
| **DYS392** | 13 | 13 | 13 |
| **DYS643** | 10 | 10 | 10 |
| **DYS393** | 13 | 13 | 13 |
| **DYS458** | 16 | 16 | 16 |
| **DYS385** | 14/16 | 14/16 | 14/16 |
| **DYS456** | 15 | 15 | 15 |
| **Y_GATA_H4** | 11 | 11 | 11 |

sample 2613

PowerPlex Fusion 6C

| ***DNA locus*** | 2613  **Blood** | 2613  **Pre vasectomy semen** | 2613  **Post vasectomy seminal fluid** |
| --- | --- | --- | --- |
| **D3S1358** | 15/17 | 15/17 | 15/17 |
| **D1S1656** | 13/17.3 | 13/17.3 | 13/17.3 |
| **D2S441** | 11/15 | 11/15 | 11/15 |
| **D10S1248** | 13 | 13 | 13 |
| **D13S317** | 11/14 | 11/14 | 11/14 |
| **Penta E** | 12/17 | 12/17 | * |
| **D16S539** | 9/12 | 9/12 | 9/12 |
| **D18S51** | 17/18 | 17/18 | 17/18 |
| **D2S1338** | 17/20 | 17/20 | 17/20 |
| **CSF1PO** | 10/11 | 10/11 | 10/11 |
| **Penta D** | 9 | 9 | 9 |
| **TH01** | 9/9.3 | 9/9.3 | 9/9.3 |
| **vWA** | 15/18 | 15/18 | 15/18 |
| **D21S11** | 29/31.2 | 29/31.2 | 29/31.2 |
| **D7S820** | 10/11 | 10/11 | 10/11 |
| **D5S818** | 10/11 | 10/11 | 10/11 |
| **TPOX** | 8/9 | 8/9 | * |
| **D8S1179** | 11/16 | 11/16 | 11/16 |
| **D12S391** | 18/19 | 18/19 | 18/19 |
| **D19S433** | 14 | 14 | 14 |
| **SE33** | 18 | 18 | 18 |
| **D22S1045** | 15 | 15 | 15 |
| **DYS391** | 10 | 10 | 10 |
| **FGA** | 21/23 | 21/23 | 21/23 |
| **DYS576** | 17 | 17 | 17 |
| **DYS570** | 18 | 18 | 18 |
| **Amelogenina** | XY  (male) | XY  (male) | XY  (male) |

PowerPlex Y23 System

| ***DNA locus* Y STR** | 2613  **Blood** | 2613  **Pre vasectomy semen** | 2613  **Post vasectomy seminal fluid** |
| --- | --- | --- | --- |
| **DYS576** | 17 | 17 | 17 |
| **DYS389I** | 13 | 13 | 13 |
| **DYS448** | 21 | 21 | 21 |
| **DYS389II** | 29 | 29 | 29 |
| **DYS19** | 15 | 15 | 15 |
| **DYS391** | 10 | 10 | 10 |
| **DYS481** | 27 | 27 | 27 |
| **DYS549** | 12 | 12 | 12 |
| **DYS533** | 10 | 10 | 10 |
| **DYS438** | 10 | 10 | 10 |
| **DYS437** | 14 | 14 | 14 |
| **DYS570** | 18 | 18 | 18 |
| **DYS635** | 21 | 21 | 21 |
| **DYS390** | 23 | 23 | 23 |
| **DYS439** | 11 | 11 | 11 |
| **DYS392** | 11 | 11 | 11 |
| **DYS643** | 9 | 9 | 9 |
| **DYS393** | 14 | 14 | 14 |
| **DYS458** | 20.2 | 20.2 | 20.2 |
| **DYS385** | 13/19 | 13/19 | 13/19 |
| **DYS456** | 15 | 15 | 15 |
| **Y_GATA_H4** | 11 | 11 | 11 |

sample 2614

PowerPlex Fusion 6C

| ***DNA locus*** | 2614  **Blood** | 2614  **Pre vasectomy semen** | 2614  **Post vasectomy seminal fluid** |
| --- | --- | --- | --- |
| **D3S1358** | 14/15 | 14/15 | 14/15 |
| **D1S1656** | 11/13 | 11/13 | 11/13 |
| **D2S441** | 10/11 | 10/11 | 10/11 |
| **D10S1248** | 13/14 | 13/14 | 13/14 |
| **D13S317** | 11 | 11 | 11 |
| **Penta E** | 7/14 | 7/14 | 7/14 |
| **D16S539** | 10/12 | 10/12 | 10/12 |
| **D18S51** | 12/14 | 12/14 | 12/14 |
| **D2S1338** | 22/25 | 22/25 | 22/25 |
| **CSF1PO** | 12 | 12 | 12 |
| **Penta D** | 11 | 11 | 11 |
| **TH01** | 6/7 | 6/7 | 6/7 |
| **vWA** | 16/18 | 16/18 | 16/18 |
| **D21S11** | 29/30.2 | 29/30.2 | 29/30.2 |
| **D7S820** | 8/10 | 8/10 | 8/10 |
| **D5S818** | 13 | 13 | 13 |
| **TPOX** | 9/11 | 9/11 | 9/11 |
| **D8S1179** | 13 | 13 | 13 |
| **D12S391** | 17/25 | 17/25 | 17/25 |
| **D19S433** | 14 | 14 | 14 |
| **SE33** | 27.2/28.2 | 27.2/28.2 | 27.2/28.2 |
| **D22S1045** | 14/15 | 14/15 | 14/15 |
| **DYS391** | 11 | 11 | 11 |
| **FGA** | 23/24 | 23/24 | 23/24 |
| **DYS576** | 16 | 16 | 16 |
| **DYS570** | 18 | 18 | 18 |
| **Amelogenina** | XY  (male) | XY  (male) | XY  (male) |

PowerPlex Y23 System

| ***DNA locus* Y STR** | 2614  **Blood** | 2614  **Pre vasectomy semen** | 2614  **Post vasectomy seminal fluid** |
| --- | --- | --- | --- |
| **DYS576** | 16 | 16 | 16 |
| **DYS389I** | 13 | 13 | 13 |
| **DYS448** | 19 | 19 | 19 |
| **DYS389II** | 29 | 29 | 29 |
| **DYS19** | 15 | 15 | 15 |
| **DYS391** | 11 | 11 | 11 |
| **DYS481** | 22 | 22 | 22 |
| **DYS549** | 12 | 12 | 12 |
| **DYS533** | 13 | 13 | 13 |
| **DYS438** | 12 | 12 | 12 |
| **DYS437** | 15 | 15 | 15 |
| **DYS570** | 18 | 18 | 18 |
| **DYS635** | 23 | 23 | 23 |
| **DYS390** | 24 | 24 | 24 |
| **DYS439** | 13 | 13 | 13 |
| **DYS392** | 13 | 13 | 13 |
| **DYS643** | 10 | 10 | 10 |
| **DYS393** | 13 | 13 | 13 |
| **DYS458** | 17 | 17 | 17 |
| **DYS385** | 11/14 | 11/14 | 11/14 |
| **DYS456** | 15 | 15 | 15 |
| **Y_GATA_H4** | 10 | 10 | 10 |

sample 2615

PowerPlex Fusion 6C

| ***DNA locus*** | 2615  **Blood** | 2615  **Pre vasectomy semen** | 2615  **Post vasectomy seminal fluid** |
| --- | --- | --- | --- |
| **D3S1358** | 14/15 | 14/15 | 14/15 |
| **D1S1656** | 14/17 | 14/17 | 14/17 |
| **D2S441** | 10/11 | 10/11 | 10/11 |
| **D10S1248** | 13/16 | 13/16 | 13/16 |
| **D13S317** | 9/11 | 9/11 | 9/11 |
| **Penta E** | 11/17 | 11/17 | 11/17 |
| **D16S539** | 9/12 | 9/12 | 9/12 |
| **D18S51** | 14/17 | 14/17 | 14/17 |
| **D2S1338** | 24/25 | 24/25 | 24/25 |
| **CSF1PO** | 10/12 | 10/12 | 10/12 |
| **Penta D** | 9/13 | 9/13 | 9/13 |
| **TH01** | 6/7 | 6/7 | 6/7 |
| **vWA** | 16/19 | 16/19 | 16/19 |
| **D21S11** | 28/30 | 28/30 | 28/30 |
| **D7S820** | 10 | 10 | 10 |
| **D5S818** | 10/12 | 10/12 | 10/12 |
| **TPOX** | 10/11 | 10/11 | 11 |
| **D8S1179** | 14/16 | 14/16 | 14/16 |
| **D12S391** | 17.3/23 | 17.3/23 | 17.3/23 |
| **D19S433** | 12.2/13 | 12.2/13 | 12.2/13 |
| **SE33** | 15/27.2 | 15/27.2 | 15/27.2 |
| **D22S1045** | 15/17 | 15/17 | 17 |
| **DYS391** | 11 | 11 | 11 |
| **FGA** | 20/23 | 20/23 | 20/23 |
| **DYS576** | 18 | 18 | 18 |
| **DYS570** | 17 | 17 | 17 |
| **Amelogenina** | XY  (male) | XY  (male) | XY  (male) |

PowerPlex Y23 System

| ***DNA locus* Y STR** | 2615  **Blood** | 2615  **Pre vasectomy semen** | 2615  **Post vasectomy seminal fluid** |
| --- | --- | --- | --- |
| **DYS576** | 18 | 18 | 18 |
| **DYS389I** | 13 | 13 | 13 |
| **DYS448** | 19 | 19 | 19 |
| **DYS389II** | 29 | 29 | 29 |
| **DYS19** | 14 | 14 | 14 |
| **DYS391** | 11 | 11 | 11 |
| **DYS481** | 22 | 22 | 22 |
| **DYS549** | 12 | 12 | 12 |
| **DYS533** | 12 | 12 | 12 |
| **DYS438** | 12 | 12 | 12 |
| **DYS437** | 15 | 15 | 15 |
| **DYS570** | 17 | 17 | 17 |
| **DYS635** | 24 | 24 | 24 |
| **DYS390** | 24 | 24 | 24 |
| **DYS439** | 12 | 12 | 12 |
| **DYS392** | 13 | 13 | 13 |
| **DYS643** | 10 | 10 | 10 |
| **DYS393** | 13 | 13 | 13 |
| **DYS458** | 17 | 17 | 17 |
| **DYS385** | 11/14 | 11/14 | 11/14 |
| **DYS456** | 16 | 16 | 16 |
| **Y_GATA_H4** | 13 | 13 | 13 |

sample 2616

PowerPlex Fusion 6C

| ***DNA locus*** | 2616  **Blood** | 2616  **Pre vasectomy semen** | 2616  **Post vasectomy seminal fluid** |
| --- | --- | --- | --- |
| **D3S1358** | 14/16 | 14/16 | 14/16 |
| **D1S1656** | 14/15 | 14/15 | 14/15 |
| **D2S441** | 10/14 | 10/14 | 10/14 |
| **D10S1248** | 15 | 15 | 15 |
| **D13S317** | 11/12 | 11/12 | 11/12 |
| **Penta E** | 16/17 | 16/17 | 16/17 |
| **D16S539** | 9/11 | 9/11 | 9/11 |
| **D18S51** | 12/18 | 12/18 | 12/18 |
| **D2S1338** | 17/26 | 17/26 | 17/26 |
| **CSF1PO** | 12 | 12 | 12 |
| **Penta D** | 9/10 | 9/10 | 9/10 |
| **TH01** | 6/9 | 6/9 | 6/9 |
| **vWA** | 18 | 18 | 18 |
| **D21S11** | 27/35 | 27/35 | 27/35 |
| **D7S820** | 10/12 | 10/12 | 10/12 |
| **D5S818** | 12 | 12 | 12 |
| **TPOX** | 8/11 | 8 | 8/11 |
| **D8S1179** | 13/14 | 13/14 | 13/14 |
| **D12S391** | 17/18 | 17/18 | 17/18 |
| **D19S433** | 13/14 | 13/14 | 13/14 |
| **SE33** | 21 | 21 | 21 |
| **D22S1045** | 10/16 | 10/16 | 10/16 |
| **DYS391** | 11 | 11 | 11 |
| **FGA** | 19/23 | 19/23 | 19/23 |
| **DYS576** | 17 | 17 | 17 |
| **DYS570** | 17 | 17 | 17 |
| **Amelogenina** | XY  (male) | XY  (male) | XY  (male) |

PowerPlex Y23 System

| ***DNA locus* Y STR** | 2616  **Blood** | 2616  **Pre vasectomy semen** | 2616  **Post vasectomy seminal fluid** |
| --- | --- | --- | --- |
| **DYS576** | 17 | 17 | 17 |
| **DYS389I** | 14 | 14 | 14 |
| **DYS448** | 19 | 19 | 19 |
| **DYS389II** | 31 | 31 | 31 |
| **DYS19** | 14 | 14 | 14 |
| **DYS391** | 11 | 11 | 11 |
| **DYS481** | 23 | 23 | 23 |
| **DYS549** | 13 | 13 | 13 |
| **DYS533** | 12 | 12 | 12 |
| **DYS438** | 12 | 12 | 12 |
| **DYS437** | 15 | 15 | 15 |
| **DYS570** | 17 | 17 | 17 |
| **DYS635** | 23 | 23 | 23 |
| **DYS390** | 24 | 24 | 24 |
| **DYS439** | 12 | 12 | 12 |
| **DYS392** | 13 | 13 | 13 |
| **DYS643** | 10 | 10 | 10 |
| **DYS393** | 13 | 13 | 13 |
| **DYS458** | 18 | 18 | 18 |
| **DYS385** | 11/13 | 11/13 | 11/13 |
| **DYS456** | 16 | 16 | 16 |
| **Y_GATA_H4** | 12 | 12 | 12 |

sample 2618

PowerPlex Fusion 6C

| ***DNA locus*** | 2618  **Blood** | 2618  **Pre vasectomy semen** | 2618  **Post vasectomy seminal fluid** |
| --- | --- | --- | --- |
| **D3S1358** | 14/16 | 14/16 | 14/16 |
| **D1S1656** | 12/14 | 12/14 | 12/14 |
| **D2S441** | 11/12 | 11/12 | 11/12 |
| **D10S1248** | 13/15 | 13/15 | 13/15 |
| **D13S317** | 11 | 11 | 11 |
| **Penta E** | 11/17 | 11/17 | 11/17 |
| **D16S539** | 10/13 | 10/13 | 10/13 |
| **D18S51** | 15/16 | 15/16 | 15/16 |
| **D2S1338** | 19/24 | 19/24 | 19/24 |
| **CSF1PO** | 10/12 | 10/12 | 10/12 |
| **Penta D** | 8/12 | 8/12 | 8/12 |
| **TH01** | 6 | 6 | 6 |
| **vWA** | 16 | 16 | 16 |
| **D21S11** | 29/30 | 29/30 | 29/30 |
| **D7S820** | 9/12 | 9/12 | 9/12 |
| **D5S818** | 12 | 12 | 12 |
| **TPOX** | 8/9 | 8/9 | 9 |
| **D8S1179** | 15 | 15 | 15 |
| **D12S391** | 20/24 | 20/24 | 20/24 |
| **D19S433** | 14 | 14 | 14 |
| **SE33** | 14/24.2 | 14/24.2 | 14/24.2 |
| **D22S1045** | 16 | 16 | 16 |
| **DYS391** | 10 | 10 | 10 |
| **FGA** | 20/21 | 20/21 | 20/21 |
| **DYS576** | 18 | 18 | 18 |
| **DYS570** | 17 | 17 | 17 |
| **Amelogenina** | XY  (male) | XY  (male) | XY  (male) |

PowerPlex Y23 System

| ***DNA locus* Y STR** | 2618  **Blood** | 2618  **Pre vasectomy semen** | 2618  **Post vasectomy seminal fluid** |
| --- | --- | --- | --- |
| **DYS576** | 18 | 18 | 18 |
| **DYS389I** | 12 | 12 | 12 |
| **DYS448** | 21 | 21 | 21 |
| **DYS389II** | 28 | 28 | 28 |
| **DYS19** | 16 | 16 | 16 |
| **DYS391** | 10 | 10 | 10 |
| **DYS481** | 23 | 23 | 23 |
| **DYS549** | 13 | 13 | 13 |
| **DYS533** | 9 | 9 | 9 |
| **DYS438** | 11 | 11 | 11 |
| **DYS437** | 16 | 16 | 16 |
| **DYS570** | 17 | 17 | 17 |
| **DYS635** | 20 | 20 | 20 |
| **DYS390** | 22 | 22 | 22 |
| **DYS439** | 11 | 11 | 11 |
| **DYS392** | 11 | 11 | 11 |
| **DYS643** | OL | 11 | 11 |
| **DYS393** | 14 | 14 | 14 |
| **DYS458** | 17 | 17 | 17 |
| **DYS385** | 14 | 14 | 14 |
| **DYS456** | 15 | 15 | 15 |
| **Y_GATA_H4** | OL | 14 | 14 |

sample 2619

PowerPlex Fusion 6C

| ***DNA locus*** | 2619  **Blood** | 2619  **Pre vasectomy semen** | 2619  **Post vasectomy seminal fluid** |
| --- | --- | --- | --- |
| **D3S1358** | 17/18 | 17/18 | 17/18 |
| **D1S1656** | 11/17.3 | 11/17.3 | 11/17.3 |
| **D2S441** | 11/14 | 11/14 | 11/14 |
| **D10S1248** | 14/15 | 14/15 | 14/15 |
| **D13S317** | 11 | 11 | 11 |
| **Penta E** | 10/13 | 10/13 | * |
| **D16S539** | 11/12 | 11/12 | 11/12 |
| **D18S51** | 15/17 | 15/17 | 15/17 |
| **D2S1338** | 20 | 20 | 20 |
| **CSF1PO** | 11/12 | 11/12 | 11/12 |
| **Penta D** | 8/13 | 8/13 | 8/13 |
| **TH01** | 6/9.3 | 6/9.3 | 6/9.3 |
| **vWA** | 15/17 | 15/17 | 15/17 |
| **D21S11** | 27/32 | 27/32 | 27/32 |
| **D7S820** | 11/13 | 11/13 | 11/13 |
| **D5S818** | 11/13 | 11/13 | 13 |
| **TPOX** | 10/11 | 11 | 10 |
| **D8S1179** | 13/16 | 13/16 | 13/16 |
| **D12S391** | 15/16 | 15/16 | 15/16 |
| **D19S433** | 15 | 15 | 15 |
| **SE33** | 27.2/30.2 | 27.2/30.2 | 27.2/30.2 |
| **D22S1045** | 15/16 | 15/16 | * |
| **DYS391** | 10 | 10 | 10 |
| **FGA** | 23/24 | 23/24 | 23/24 |
| **DYS576** | 18 | 18 | 18 |
| **DYS570** | 16 | 16 | 16 |
| **Amelogenina** | XY  (male) | XY  (male) | XY  (male) |

PowerPlex Y23 System

| ***DNA locus* Y STR** | 2619  **Blood** | 2619  **Pre vasectomy semen** | 2619  **Post vasectomy seminal fluid** |
| --- | --- | --- | --- |
| **DYS576** | 18 | 18 | 18 |
| **DYS389I** | 13 | 13 | 13 |
| **DYS448** | 19 | 19 | 19 |
| **DYS389II** | 31 | 31 | 31 |
| **DYS19** | 13 | 13 | 13 |
| **DYS391** | 10 | 10 | 10 |
| **DYS481** | 26 | 26 | 26 |
| **DYS549** | 13 | 13 | 13 |
| **DYS533** | 11 | 11 | 11 |
| **DYS438** | 11 | 11 | 11 |
| **DYS437** | 13 | 13 | 13 |
| **DYS570** | 16 | 16 | 16 |
| **DYS635** | 22 | 22 | 22 |
| **DYS390** | 24 | 24 | 24 |
| **DYS439** | 12 | 12 | 12 |
| **DYS392** | 14 | 14 | 14 |
| **DYS643** | 10 | 10 | 10 |
| **DYS393** | 13 | 13 | 13 |
| **DYS458** | 16 | 16 | 16 |
| **DYS385** | 15/16 | 15/16 | 15/16 |
| **DYS456** | 15 | 15 | 15 |
| **Y_GATA_H4** | 11 | 11 | 11 |

sample 2620

PowerPlex Fusion 6C

| ***DNA locus*** | 2620  **Blood** | 2620  **Pre vasectomy semen** | 2620  **Post vasectomy seminal fluid** |
| --- | --- | --- | --- |
| **D3S1358** | 15/16 | 15/16 | 15/16 |
| **D1S1656** | 15/15.3 | 15/15.3 | 15/15.3 |
| **D2S441** | 14/15 | 14/15 | 14/15 |
| **D10S1248** | 13/16 | 13/16 | 13/16 |
| **D13S317** | 11/13 | 11/13 | 11/13 |
| **Penta E** | 7/10 | 7/10 | 10 |
| **D16S539** | 12 | 12 | 12 |
| **D18S51** | 19/21 | 19/21 | 19/21 |
| **D2S1338** | 17/20 | 17/20 | 17/20 |
| **CSF1PO** | 10 | 10 | 10 |
| **Penta D** | 12/13 | 12/13 | 12/13 |
| **TH01** | 6/7 | 6/7 | 6/7 |
| **vWA** | 17/19 | 17/19 | 17/19 |
| **D21S11** | 27/31 | 27/31 | 27/31 |
| **D7S820** | 8/10 | 8/10 | 8/10 |
| **D5S818** | 13 | 13 | 13 |
| **TPOX** | 8/12 | 8/12 | 8 |
| **D8S1179** | 11/12 | 11/12 | 11/12 |
| **D12S391** | 16/20 | 16/20 | 16/20 |
| **D19S433** | 13/16.2 | 13/16.2 | 13/16.2 |
| **SE33** | 17/23.2 | 17/23.2 | 17/23.2 |
| **D22S1045** | 15/18 | 15/18 | * |
| **DYS391** | 11 | 11 | 11 |
| **FGA** | 19/28 | 19/28 | 19/28 |
| **DYS576** | 17 | 17 | 17 |
| **DYS570** | 17 | 17 | 17 |
| **Amelogenina** | XY  (male) | XY  (male) | XY  (male) |

PowerPlex Y23 System

| ***DNA locus* Y STR** | 2620  **Blood** | 2620  **Pre vasectomy semen** | 2620  **Post vasectomy seminal fluid** |
| --- | --- | --- | --- |
| **DYS576** | 17 | 17 | 17 |
| **DYS389I** | 13 | 13 | 13 |
| **DYS448** | 20 | 20 | 20 |
| **DYS389II** | 29 | 29 | 29 |
| **DYS19** | 14 | 14 | 14 |
| **DYS391** | 11 | 11 | 11 |
| **DYS481** | 22 | 22 | 22 |
| **DYS549** | 13 | 13 | 13 |
| **DYS533** | 12 | 12 | 12 |
| **DYS438** | 12 | 12 | 12 |
| **DYS437** | 15 | 15 |  |
| **DYS570** | 17 | 17 | 17 |
| **DYS635** | 23 | 23 | 23 |
| **DYS390** | 24 | 24 | 24 |
| **DYS439** | 12 | 12 | 12 |
| **DYS392** | 13 | 13 | 13 |
| **DYS643** | 10 | 10 | 10 |
| **DYS393** | 13 | 13 | 13 |
| **DYS458** | 17 | 17 | 17 |
| **DYS385** | 11/14 | 11/14 | 11/14 |
| **DYS456** | 16 | 16 | 16 |
| **Y_GATA_H4** | 11 | 11 | 11 |

sample 2622

PowerPlex Fusion 6C

| ***DNA locus*** | 2622  **Blood** | 2622  **Pre vasectomy semen** | 2622  **Post vasectomy seminal fluid** |
| --- | --- | --- | --- |
| **D3S1358** | 16/17 | 16/17 | 16/17 |
| **D1S1656** | 14.3/16.3 | 14.3/16.3 | 14.3/16.3 |
| **D2S441** | 12 | 12 | 12 |
| **D10S1248** | 14 | 14 | 14 |
| **D13S317** | 11 | 11 | 11 |
| **Penta E** | 9/10 | 9/10 | 10 |
| **D16S539** | 10/12 | 10/12 | 10/12 |
| **D18S51** | 15/16 | 15/16 | 15/16 |
| **D2S1338** | 20/22 | 20/22 | 20/22 |
| **CSF1PO** | 11/13 | 11/13 | 11/13 |
| **Penta D** | 5/13 | 5/13 | 5/13 |
| **TH01** | 6/9 | 6/9 | 6/9 |
| **vWA** | 14/17 | 14/17 | 14/17 |
| **D21S11** | 28/31.2 | 28/31.2 | 28/31.2 |
| **D7S820** | 10 | 10 | 10 |
| **D5S818** | 12 | 12 | 12 |
| **TPOX** | 8/11 | 8/11 | 8 |
| **D8S1179** | 11 | 11 | 11 |
| **D12S391** | 17/19 | 17/19 | 17/19 |
| **D19S433** | 13 | 13 | 13 |
| **SE33** | 18/20 | 18/20 | 18/20 |
| **D22S1045** | 14/15 | 14/15 | * |
| **DYS391** | 11 | 11 | 11 |
| **FGA** | 23/24 | 23/24 | 23/24 |
| **DYS576** | 18 | 18 | 18 |
| **DYS570** | 17 | 17 | * |
| **Amelogenina** | XY  (male) | XY  (male) | XY  (male) |

PowerPlex Y23 System

| ***DNA locus* Y STR** | 2622  **Blood** | 2622  **Pre vasectomy semen** | 2622  **Post vasectomy seminal fluid** |
| --- | --- | --- | --- |
| **DYS576** | 18 | 18 | 18 |
| **DYS389I** | 12 | 12 | 12 |
| **DYS448** | 19 | 19 | 19 |
| **DYS389II** | 26 | 26 | 26 |
| **DYS19** | 15 | 15 | 15 |
| **DYS391** | 11 | 11 | 11 |
| **DYS481** | 22 | 22 | 22 |
| **DYS549** | 13 | 13 | 13 |
| **DYS533** | 11 | 11 | 11 |
| **DYS438** | 12 | 12 | 12 |
| **DYS437** | 15 | 15 | 15 |
| **DYS570** | 17 | 17 | 17 |
| **DYS635** | 24 | 24 | 24 |
| **DYS390** | 24 | 24 | 24 |
| **DYS439** | 10 | 10 | 10 |
| **DYS392** | 13 | 13 | 13 |
| **DYS643** | 10 | 10 | 10 |
| **DYS393** | 13 | 13 | 13 |
| **DYS458** | 16 | 16 | 16 |
| **DYS385** | 12/14 | 12/14 | 12/14 |
| **DYS456** | 15 | 15 | 15 |
| **Y_GATA_H4** | 12 | 12 | 12 |

sample 2624

PowerPlex Fusion 6C

| ***DNA locus*** | 2624  **Blood** | 2624  **Pre vasectomy semen** | 2624  **Post vasectomy seminal fluid** |
| --- | --- | --- | --- |
| **D3S1358** | 15/18 | 15/18 | 15/18 |
| **D1S1656** | 10/15 | 10/15 | 10/15 |
| **D2S441** | 14/15 | 14/15 | 14/15 |
| **D10S1248** | 13/15 | 13/15 | 13/15 |
| **D13S317** | 9/13 | 9/13 | * |
| **Penta E** | 17 | 7/17 | * |
| **D16S539** | 11/13 | 11/13 | 11/13 |
| **D18S51** | 17 | 17 | 17 |
| **D2S1338** | 17/22 | 17/22 | 17 |
| **CSF1PO** | 10/12 | 11/12 | 12 |
| **Penta D** | 8/10 | 8/10 | * |
| **TH01** | 7/9 | 7/9 | 7/9 |
| **vWA** | 15/20 | 15/20 | 15/20 |
| **D21S11** | 28/30 | 28/30 | 28/30 |
| **D7S820** | 8 | 8 | 8 |
| **D5S818** | 12 | 12 | * |
| **TPOX** | 8/11 | 8/11 | * |
| **D8S1179** | 13/14 | 13/14 | 13/14 |
| **D12S391** | 21/24 | 21/24 | 21/24 |
| **D19S433** | 13 | 13 | 13 |
| **SE33** | 16/17 | 16/17 | * |
| **D22S1045** | 15 | 15 | * |
| **DYS391** | 10 | 10 | 10 |
| **FGA** | 19/23 | 19/23 | 19/23 |
| **DYS576** | 18 | 18 | * |
| **DYS570** | 18 | 18 | * |
| **Amelogenina** | XY  (male) | XY  (male) | XY  (male) |

PowerPlex Y23 System

| ***DNA locus* Y STR** | 2624  **Blood** | 2624  **Pre vasectomy semen** | 2624  **Post vasectomy seminal fluid** |
| --- | --- | --- | --- |
| **DYS576** | 18 | 18 | 18 |
| **DYS389I** | 13 | 13 | 13 |
| **DYS448** | 21 | 21 | 21 |
| **DYS389II** | 30 | 30 |  |
| **DYS19** | 16 | 16 | 16 |
| **DYS391** | 10 | 10 | 10 |
| **DYS481** | 25 | 25 | 25 |
| **DYS549** | 11 | 11 | 11 |
| **DYS533** | 11 | 11 | 11 |
| **DYS438** | 11 | 11 | 11 |
| **DYS437** | 14 | 14 |  |
| **DYS570** | 18 | 18 | 18 |
| **DYS635** | 21 | 21 | 21 |
| **DYS390** | 20 | 20 | 20 |
| **DYS439** | 13 | 13 | 13 |
| **DYS392** | 11 | 11 |  |
| **DYS643** | 13 | 15 |  |
| **DYS393** | 15 | 16 | 15 |
| **DYS458** | 16 | 16 | 16 |
| **DYS385** | 16/18 | 16/18 | 16/18 |
| **DYS456** | 15 | 15 |  |
| **Y_GATA_H4** | 11 | 11 | 11 |

sample 2626

PowerPlex Fusion 6C

| ***DNA locus*** | 2626  **Blood** | 2626  **Pre vasectomy semen** | 2626  **Post vasectomy seminal fluid** |
| --- | --- | --- | --- |
| **D3S1358** | 14/18 | 14/18 | 14/18 |
| **D1S1656** | 15/18.3 | 15/18.3 | 15/18.3 |
| **D2S441** | 10/11 | 10/11 | 10/11 |
| **D10S1248** | 14/15 | 14/15 | 14/15 |
| **D13S317** | 11 | 11 | 11 |
| **Penta E** | 15/19 | 15/19 | 15/19 |
| **D16S539** | 11/12 | 11/12 | 11/12 |
| **D18S51** | 16/17 | 16/17 | 16/17 |
| **D2S1338** | 18/25 | 18/25 | 18/25 |
| **CSF1PO** | 12 | 8/12 | 8/12 |
| **Penta D** | 9/11 | 9/11 | 9/11 |
| **TH01** | 9 | 9 | 9 |
| **vWA** | 15/17 | 15/17 | 15/17 |
| **D21S11** | 29/30 | 29/30 | 29/30 |
| **D7S820** | 8/11 | 8/11 | 8/11 |
| **D5S818** | 11/13 | 11/13 | 11/13 |
| **TPOX** | 6/9 | 6/9 | 6/9 |
| **D8S1179** | 14 | 14 | 14 |
| **D12S391** | 16/18 | 16/18 | 16/18 |
| **D19S433** | 12/15 | 12/15 | 12/15 |
| **SE33** | 16/23.2 | 16/23.2 | 16/23.2 |
| **D22S1045** | 11/16 | 11/16 | 11/16 |
| **DYS391** | 11 | 11 | 11 |
| **FGA** | 20/23 | 20/23 | 20/23 |
| **DYS576** | 18 | 18 | 18 |
| **DYS570** | 17 | 17 | 17 |
| **Amelogenina** | XY  (male) | XY  (male) | XY  (male) |

PowerPlex Y23 System

| ***DNA locus* Y STR** | 2626  **Blood** | 2626  **Pre vasectomy semen** | 2626  **Post vasectomy seminal fluid** |
| --- | --- | --- | --- |
| **DYS576** | 18 | 18 | 18 |
| **DYS389I** | 13 | 13 | 13 |
| **DYS448** | 19 | 19 | 19 |
| **DYS389II** | 29 | 29 | 29 |
| **DYS19** | 14 | 14 | 14 |
| **DYS391** | 11 | 11 | 11 |
| **DYS481** | 22 | 22 | 22 |
| **DYS549** | 13 | 13 | 13 |
| **DYS533** | 13 | 13 | 13 |
| **DYS438** | 12 | 12 | 12 |
| **DYS437** | 15 | 15 | 15 |
| **DYS570** | 17 | 17 | 17 |
| **DYS635** | 23 | 23 | 23 |
| **DYS390** | 24 | 24 | 24 |
| **DYS439** | 12 | 12 | 12 |
| **DYS392** | 13 | 13 | 13 |
| **DYS643** | 10 | 10 | 10 |
| **DYS393** | 13 | 13 | 13 |
| **DYS458** | 17 | 17 | 17 |
| **DYS385** | 11/14 | 11/14 | 11/14 |
| **DYS456** | 16 | 16 | 16 |
| **Y_GATA_H4** | 12 | 12 | 12 |

sample 2627

PowerPlex Fusion 6C

| ***DNA locus*** | 2627  **Blood** | 2627  **Pre vasectomy semen** | 2627  **Post vasectomy seminal fluid** |
| --- | --- | --- | --- |
| **D3S1358** | 18 | 18 | 18 |
| **D1S1656** | 14/15.3 | 14/15.3 | 14/15.3 |
| **D2S441** | 11/12 | 11/12 | 11/12 |
| **D10S1248** | 12/14 | 12/14 | 12/14 |
| **D13S317** | 11/12 | 11/12 | 11/12 |
| **Penta E** | 11/13 | 11 | * |
| **D16S539** | 11/13 | 11/13 | 11/13 |
| **D18S51** | 12/14 | 12/14 | 12/14 |
| **D2S1338** | 18/19 | 18/19 | 18/19 |
| **CSF1PO** | 12 | 12 | 12 |
| **Penta D** | 11/13 | 11/13 | 11/13 |
| **TH01** | 9.3 | 9.3 | 9.3 |
| **vWA** | 16/17 | 16/17 | 16/17 |
| **D21S11** | 30/32.2 | 30/32.2 | 30/32.2 |
| **D7S820** | 11 | 11 | 11 |
| **D5S818** | 9/10 | 9/10 | 9/10 |
| **TPOX** | 8/9 | * | 8 |
| **D8S1179** | 13/16 | 13/16 | 13/16 |
| **D12S391** | 17/18 | 17/18 | 17/18 |
| **D19S433** | 13/15 | 13/15 | 13/15 |
| **SE33** | 15/27.2 | 15/27.2 | 15/27.2 |
| **D22S1045** | 11/16 | * | * |
| **DYS391** | 11 | 11 | 11 |
| **FGA** | 21 | 21 | 21 |
| **DYS576** | 20 | 20 | 20 |
| **DYS570** | 16 | 16 | 16 |
| **Amelogenina** | XY  (male) | XY  (male) | XY  (male) |

PowerPlex Y23 System

| ***DNA locus* Y STR** | 2627  **Blood** | 2627  **Pre vasectomy semen** | 2627  **Post vasectomy seminal fluid** |
| --- | --- | --- | --- |
| **DYS576** | 20 | * | 20 |
| **DYS389I** | 14 | * | 14 |
| **DYS448** | 19 | * | 19 |
| **DYS389II** | 30 | * |  |
| **DYS19** | 14 | * |  |
| **DYS391** | 11 | * | 11 |
| **DYS481** | 22 | * | 22 |
| **DYS549** | 13 | * | 13 |
| **DYS533** | 12 | * | 12 |
| **DYS438** | 12 | * | 12 |
| **DYS437** | 15 | * |  |
| **DYS570** | 16 | * | 16 |
| **DYS635** | 23 | * | 23 |
| **DYS390** | 24 | * | 24 |
| **DYS439** | 12 | * | 12 |
| **DYS392** | 13 | * |  |
| **DYS643** | 10 | * |  |
| **DYS393** | 13 | * | 13 |
| **DYS458** | 17 | * | 17 |
| **DYS385** | 11/14 | * | 11/14 |
| **DYS456** | 15 | * | 15 |
| **Y_GATA_H4** | 11 | * | 11 |

sample 2629

PowerPlex Fusion 6C

| ***DNA locus*** | 2629  **Blood** | 2629  **Pre vasectomy semen** | 2629  **Post vasectomy seminal fluid** |
| --- | --- | --- | --- |
| **D3S1358** | 17/18 | 17/18 | 17/18 |
| **D1S1656** | 11/12 | 11/12 | 11/12 |
| **D2S441** | 11/12 | 11/12 | 11/12 |
| **D10S1248** | 14/16 | 14/16 | 14/16 |
| **D13S317** | 11/12 | 11/12 | 11/12 |
| **Penta E** | 9/19 | 9/19 | 9/19 |
| **D16S539** | 12 | 12 | 12 |
| **D18S51** | 12/18 | 12/18 | 12/18 |
| **D2S1338** | 19/23 | 19/23 | 19/23 |
| **CSF1PO** | 10/13 | 10/13 | 10/13 |
| **Penta D** | 10/13 | 10/13 | 10 |
| **TH01** | 6/9.3 | 6/9.3 | 6/9.3 |
| **vWA** | 17/18 | 17/18 | 17/18 |
| **D21S11** | 32.2 | 32.2 | 32.2 |
| **D7S820** | 9/11 | 9/11 | 9/11 |
| **D5S818** | 11/13 | 11/13 | 11/13 |
| **TPOX** | 8/11 | 8/11 | * |
| **D8S1179** | 14/16 | 14/16 | 14/16 |
| **D12S391** | 15/19 | 15/19 | 15/19 |
| **D19S433** | 11/14 | 11/14 | 11/14 |
| **SE33** | 15/30.2 | 15/30.2 | 15/30.2 |
| **D22S1045** | 11/16 | * | 11/16 |
| **DYS391** | 10 | 10 | 10 |
| **FGA** | 21/25 | 21/25 | 21/25 |
| **DYS576** | 16 | 16 | 16 |
| **DYS570** | 19 | 19 | 19 |
| **Amelogenina** | XY  (male) | XY  (male) | XY  (male) |

PowerPlex Y23 System

| ***DNA locus* Y STR** | 2629  **Blood** | 2629  **Pre vasectomy semen** | 2629  **Post vasectomy seminal fluid** |
| --- | --- | --- | --- |
| **DYS576** | 16 | 16 | 16 |
| **DYS389I** | 12 | 12 | 12 |
| **DYS448** | 21 | 21 | 21 |
| **DYS389II** | 29 | 29 | 29 |
| **DYS19** | 15 | 15 |  |
| **DYS391** | 10 | 10 | 10 |
| **DYS481** | 23 | 23 | 23 |
| **DYS549** | 13 | 13 | 13 |
| **DYS533** | 10 | 10 | 10 |
| **DYS438** | 10 | 10 | 10 |
| **DYS437** | 16 |  | 16 |
| **DYS570** | 19 | 19 | 19 |
| **DYS635** | 22 | 22 | 22 |
| **DYS390** | 23 | 23 | 23 |
| **DYS439** | 13 | 13 | 13 |
| **DYS392** | 11 | 11 | 7 |
| **DYS643** | 11 |  | OL |
| **DYS393** | 14 | 14 | 14 |
| **DYS458** | 15 | 15 | 15 |
| **DYS385** | 14/15 | 14/15 | 14/15 |
| **DYS456** | 15 | 15 | 15 |
| **Y_GATA_H4** | 12 | 12 | 15 |

sample 2630

PowerPlex Fusion 6C

| ***DNA locus*** | 2630  **Blood** | 2630  **Pre vasectomy semen** | 2630  **Post vasectomy seminal fluid** |
| --- | --- | --- | --- |
| **D3S1358** | 15 | 15 | 15 |
| **D1S1656** | 13/16.3 | 13/16.3 | 13/16.3 |
| **D2S441** | 11 | 11 | 11 |
| **D10S1248** | 14/17 | 14/17 | 14/17 |
| **D13S317** | 11 | 11 | 11 |
| **Penta E** | 7/12 | 7/12 | 7/12 |
| **D16S539** | 12/14 | 12/14 | 12/14 |
| **D18S51** | 14/17 | 14/17 | 14/17 |
| **D2S1338** | 17/24 | 17/24 | 17/24 |
| **CSF1PO** | 11 | 11 | 11 |
| **Penta D** | 12/13 | 12/13 | 12/13 |
| **TH01** | 8/9 | 8/9 | 8/9 |
| **vWA** | 16 | 16 | 16 |
| **D21S11** | 28/29 | 28/29 | 28/29 |
| **D7S820** | 12/13 | 12/13 | 12/13 |
| **D5S818** | 11/12 | 11/12 | 11/12 |
| **TPOX** | 8/11 | 8 | 8/11 |
| **D8S1179** | 13 | 13 | 13 |
| **D12S391** | 20/22 | 20/22 | 20/22 |
| **D19S433** | 13 | 13 | 13 |
| **SE33** | 16/17 | 16/17 | 16/17 |
| **D22S1045** | 15 | 15 | 15 |
| **DYS391** | 11 | 11 | 11 |
| **FGA** | 21/23 | 21/23 | 21/23 |
| **DYS576** | 18 | 18 | 18 |
| **DYS570** | 19 | 19 | 19 |
| **Amelogenina** | XY  (male) | XY  (male) | XY  (male) |

PowerPlex Y23 System

| ***DNA locus* Y STR** | 2630  **Blood** | 2630  **Pre vasectomy semen** | 2630  **Post vasectomy seminal fluid** |
| --- | --- | --- | --- |
| **DYS576** | 18 | 18 | 18 |
| **DYS389I** | 13 | 13 | 13 |
| **DYS448** | 18 | 18 | 18 |
| **DYS389II** | 29 | 29 | 29 |
| **DYS19** | 14 | 14 | 14 |
| **DYS391** | 11 | 11 | 11 |
| **DYS481** | 22 | 22 | 22 |
| **DYS549** | 12 | 12 | 12 |
| **DYS533** | 12 | 12 | 12 |
| **DYS438** | 12 | 12 | 12 |
| **DYS437** | 14 | 14 | 14 |
| **DYS570** | 19 | 19 | 19 |
| **DYS635** | 23 | 23 | 23 |
| **DYS390** | 25 | 25 | 25 |
| **DYS439** | 11 | 11 | 11 |
| **DYS392** | 13 | 13 | 13 |
| **DYS643** | 10 | 10 | 10 |
| **DYS393** | 13 | 13 | 13 |
| **DYS458** | 17 | 17 | 17 |
| **DYS385** | 11/12 | 11/12 | 11/12 |
| **DYS456** | 17 | 17 | 17 |
| **Y_GATA_H4** | 11 | 11 | 11 |

sample 2655

PowerPlex Fusion 6C

| ***DNA locus*** | 2655  **Blood** | 2655  **Pre vasectomy semen** | 2655  **Post vasectomy seminal fluid** |
| --- | --- | --- | --- |
| **D3S1358** | 16 | 16 | 16 |
| **D1S1656** | 16/17.3 | 16/17.3 | 16/17.3 |
| **D2S441** | 10/14 | 10/14 | 10/14 |
| **D10S1248** | 12/15 | 12/15 | 12/15 |
| **D13S317** | 10/11 | 10/11 | 10/11 |
| **Penta E** | 13/17 | 13/17 | 13/17 |
| **D16S539** | 9 | 9 | 9 |
| **D18S51** | 10.2/12/16 | 12/16 | 12/16 |
| **D2S1338** | 19/22 | 19/22 | 19/22 |
| **CSF1PO** | 12 | 12 | 12 |
| **Penta D** | 7/11 | 7/11 | 7/11 |
| **TH01** | 7/9.3 | 7/9.3 | 7/9.3 |
| **vWA** | 15 | 15 | 15 |
| **D21S11** | 29/31 | 29/31 | 29/31 |
| **D7S820** | 9/11 | 9/11 | 9/11 |
| **D5S818** | 12/13 | 12/13 | 12/13 |
| **TPOX** | 8/11 | 8/11 | * |
| **D8S1179** | 15 | 13/15 | 13/15 |
| **D12S391** | 17/18 | 17/18 | 17/18 |
| **D19S433** | 13/15 | 13/15 | 13/15 |
| **SE33** | 16/19 | 16/19 | 16/19 |
| **D22S1045** | 14/15 | 14/15 | * |
| **DYS391** | 11 | 11 | 11 |
| **FGA** | 19/29 | 19/29 | 19/29 |
| **DYS576** | 18 | 18 | 18 |
| **DYS570** | 18 | 18 | * |
| **Amelogenina** | XY  (male) | XY  (male) | XY  (male) |

PowerPlex Y23 System

| ***DNA locus* Y STR** | 2655  **Blood** | 2655  **Pre vasectomy semen** | 2655  **Post vasectomy seminal fluid** |
| --- | --- | --- | --- |
| **DYS576** | 18 | 18 | 18 |
| **DYS389I** | 13 | 13 | 13 |
| **DYS448** | 19 | 19 | 19 |
| **DYS389II** | 29 | 29 | 29 |
| **DYS19** | 14 | 14 | 14 |
| **DYS391** | 11 | 11 | 11 |
| **DYS481** | 20 | 20 | 20 |
| **DYS549** | 14 | 14 | 14 |
| **DYS533** | 12 | 12 | 12 |
| **DYS438** | 12 | 12 | 12 |
| **DYS437** | 15 | 15 | 15 |
| **DYS570** | OL | OL | 18 |
| **DYS635** | 23 | 23 | 23 |
| **DYS390** | 24 | 24 | 24 |
| **DYS439** | 12 | 12 | 12 |
| **DYS392** | 13 | 13 | 13 |
| **DYS643** | 10 | 10 | 10 |
| **DYS393** | OL | OL | 12 |
| **DYS458** | 16 | 16 | 16 |
| **DYS385** | 11/14 | 11/14 | 11/14 |
| **DYS456** | 15 | 15 | 15 |
| **Y_GATA_H4** | 13 | 13 | 13 |

sample 2657

PowerPlex Fusion 6C

| ***DNA locus*** | 2657  **Blood** | 2657  **Pre vasectomy semen** | 2657  **Post vasectomy seminal fluid** |
| --- | --- | --- | --- |
| **D3S1358** | 15/16 | 15/16 | 15/16 |
| **D1S1656** | 11/17.3 | 11/17.3 | 11/17.3 |
| **D2S441** | 12/15 | 12/15 | 12/15 |
| **D10S1248** | 13/15 | 13/15 | 13/15 |
| **D13S317** | 8/12 | 8/12 | 8 |
| **Penta E** | 7/8 | 7/8 | * |
| **D16S539** | 12/13 | 12/13 | 12/13 |
| **D18S51** | 13/16 | 13/16 | 13/16 |
| **D2S1338** | 17 | 17 | 17 |
| **CSF1PO** | 12 | 12 | 12 |
| **Penta D** | 9/13 | 9/13 | 9 |
| **TH01** | 7/9.3 | 7/9.3 | 7/9.3 |
| **vWA** | 16/18 | 16/18 | 16/18 |
| **D21S11** | 29/34 | 29/34 | * |
| **D7S820** | 11/13 | 11/13 | 13 |
| **D5S818** | 12 | 12 | * |
| **TPOX** | 8/11 | 8/11 | 8 |
| **D8S1179** | 13 | 13 | 13 |
| **D12S391** | 18/19.3 | 18/19.3 | 18/19.3 |
| **D19S433** | 13.2/14 | 13.2/14 | 13.2/14 |
| **SE33** | 18/21.2 | 18/21.2 | 21.2 |
| **D22S1045** | 15/16 | 15/16 | * |
| **DYS391** | 11 | 11 | 11 |
| **FGA** | 21/25 | 21/25 | 21/25 |
| **DYS576** | 18 | 18 | 18 |
| **DYS570** | 17 | 17 | 17 |
| **Amelogenina** | XY  (male) |  | XY  (male) |

PowerPlex Y23 System

| ***DNA locus* Y STR** | 2657  **Blood** | 2657  **Pre vasectomy semen** | 2657  **Post vasectomy seminal fluid** |
| --- | --- | --- | --- |
| **DYS576** | 18 | 18 | 18 |
| **DYS389I** | 13 | 13 | 13 |
| **DYS448** | 19 | 19 | 19 |
| **DYS389II** | 29 | 29 | 29 |
| **DYS19** | 14 | 14 | 14 |
| **DYS391** | 11 | 11 | 11 |
| **DYS481** | 22 | 22 | 22 |
| **DYS549** | 14 | 14 | 14 |
| **DYS533** | 12 | 12 | 12 |
| **DYS438** | 13 | 13 | 13 |
| **DYS437** | 15 | 15 | 15 |
| **DYS570** | 17 | 17 | 17 |
| **DYS635** | 23 | 23 | 23 |
| **DYS390** | 24 | 24 | 24 |
| **DYS439** | 12 | 12 | 12 |
| **DYS392** | 13 | 13 | 13 |
| **DYS643** | 10 | 10 |  |
| **DYS393** | 13 | 13 | 13 |
| **DYS458** | 17 | 17 | 17 |
| **DYS385** | 11/14 | 11/14 | 11/14 |
| **DYS456** | 17 | 17 | 17 |
| **Y_GATA_H4** | 12 | 12 | 12 |
